# Cold‐Captured Dynamic Hydration Networks in Oxime‐Based Photoswitches: A Theoretical Challenge Uncovered by Rotational Spectroscopy

**DOI:** 10.1002/anie.202513560

**Published:** 2025-09-06

**Authors:** Rita J. C. Roque, Nuno M. Campos, Marcos Gouveia, Sérgio R. Domingos

**Affiliations:** ^1^ CFisUC Department of Physics University of Coimbra 3004‐516 Coimbra Portugal

**Keywords:** Artificial molecular motors, Microsolvation, Noncovalent interactions, Quantum chemistry calculations, Rotational spectroscopy

## Abstract

With the goal of manipulating (bio)chemical processes, photoswitches emerge as important assets in molecular nanotechnology. To guide synthetic strategies toward increasingly more efficient systems, conformational dynamics studies performed with atomic rigor are in demand, particularly if this information can be extracted with control over the size of a perturbing solvation layer. Here, we use jet‐cooled rotational spectroscopy and quantum chemistry calculations to unravel the structure and micro‐hydration dynamics of a prototype photoswitch. Camphorquinone‐oxime has a switching function enabled by the oxime moiety, and a chiral subspace generated by camphor, ensuring motion directionality. Although it may seem a relatively simple molecule, several popular levels of theory disagree on the energy ordering of the two switch states. We find that the oxime moiety integrates cooperatively into linear water chains captured for the dimer and trimer topologies, as well as into more exotic three‐dimensional structures created for the complex with the water tetramer. Evidence for concerted hydration dynamics emerges from a comparison between theory and experimental isotopic information. We evaluate the balance of intermolecular forces at play during the hydration network build up and discuss how a flexible first solvation layer may affect the switching dynamics of this class of systems.

## Graphical abstract

By combining gas‐phase rotational spectroscopy and quantum chemistry calculations, we explore how a prototype molecular photoswitch is affected by microhydration. This study unveils the theory‐breaking structural features of camphorquinone oxime, the concerted dynamics of the surrounding water network, and how the oxime subunit actively steers the geometry of the first hydration shell.

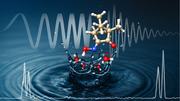

## Introduction

Inspired by many biological processes, new developments in molecular nanotechnology have been focused on novel molecular machines that convert energy into controlled motion with the goal of manipulating macroscopic systems using nanoscale tools.^[^ ^1^, ^2^ ^]^ Examples include responsive drug‐delivery systems,^[^ ^1^ ^]^ molecular computers,^[^ ^3^ ^]^ DNA hybridization,^[^ ^4^ ^]^ and even nano‐cars.^[^ ^5^ ^]^ To guide the synthesis of such complex and targeted systems, it is fundamental to study the intricate working mechanisms that drive these molecular architectures.^[^ ^6^, ^7^, ^8^ ^]^

Some of the simplest synthetic molecular motors are the ones that comprise a C═N bond, where isomerization is achieved either by photo‐induced out‐of‐plane rotation (confirmed only theoretically) or by thermal in‐plane inversion.^[^ ^9^, ^10^, ^11^, ^12^, ^13^ ^]^ It has been proposed that an asymmetrical potential energy surface, such as by the introduction of a nearby chiral center, may imprint a preferred directionality to the photo‐induced C═N bond rotation. If this conjecture is true, the process of photo‐induced isomerization followed by thermal relaxation generates an unidirectional, motor‐like motion that can be triggered by light. The proposal that any chiral molecule containing a C═N bond is a candidate photo‐driven molecular motor extends to all chiral imine‐derived molecular systems, including aldimines, hydrazones, azines, and oximes.^[^ ^13^ ^]^

Many nanotechnology applications rely on molecular motors being introduced in complex environments without loosing their intended functionality. With this in mind, it becomes imperative to evaluate the structure of relevant micro‐solvated species, to ensure that a molecular motor is not “jammed” by the surrounding environment. One of the most concerning cases is the presence of water, which is ubiquitous in biological systems and capable of triggering significant structural changes in molecular systems.^[^ ^14^, ^15^, ^16^, ^17^ ^]^ There is evidence that micro‐solvation can significantly influence the workings of molecular motors; recent findings show that two water molecules are enough to drive an equilibrium‐state reversal of an imine‐based molecular switch.^[^ ^18^ ^]^

Although water‐containing jet‐cooled gas‐phase complexes are usually thought of as static systems, this assessment should be carefully considered as it may provide an incomplete picture; recent studies show that partially unlocked water molecules undergo significant motions even when interacting with relatively rigid molecules. If symmetry and the energetic barriers allow, these rearrangements of the solvating network may occur through quantum tunneling, such as the reorientation^[^ ^19^ ^]^ or the puckering motions of water molecules in the pure water pentamer,^[^ ^20^ ^]^ hexamer,^[^ ^21^, ^22^ ^]^ or in the benzaldehyde–($H_{2}O$)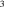 complex.^[^ ^23^ ^]^ In cases where the local symmetry is destroyed by a chiral subspace and the energy barriers between equilibrium states are low enough, observables are pushed toward vibrationally averaged molecular conformations, as observed in several hydrated species like camphorquinone‐imine,^[^ ^18^ ^]^ the trans 1,1,1,3,3,3‐hexafluoro‐2‐propanol,^[^ ^24^ ^]^ and in 2‐fluoroethanol.^[^ ^25^ ^]^

When coupled with computational calculations, broadband molecular rotational resonance (MRR) spectroscopy^[^ ^26^ ^]^ is a powerful tool that can unlock the structure and configurational dynamics of molecular systems, since it directly captures the moments of inertia of the entire molecule. This technique has already been validated for the study of molecular motors^[^ ^18^, ^27^ ^]^ and of weakly‐bound systems in the gas phase, including the study of pure water clusters up to the water decamer^[^ ^21^, ^22^, ^28^, ^29^, ^30^ ^]^ and of several micro‐solvated species.^[^ ^18^, ^19^, ^23^, ^31^, ^32^, ^33^, ^34^, ^35^, ^36^, ^37^, ^38^ ^]^ In cases where the shift in the overall molecular moments of inertia is too subtle, structural information with atomic resolution can be obtained from the satellite rotational spectra of singly‐substituted isotopologues via Kraitchman analysis.^[^ ^39^, ^40^ ^]^ Solving the position of key atoms in a molecular system can often settle disputes on nearly identical conformations, or even unveil underlying dynamics.^[^ ^19^, ^21^, ^22^ ^]^

**Figure 1:**
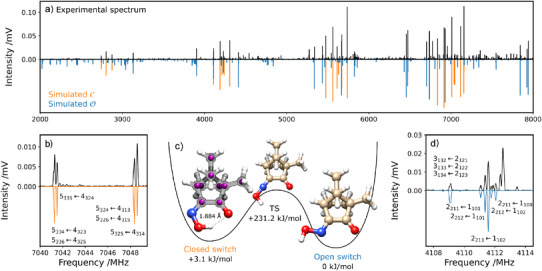
Rotational spectra and structures of the two switch forms. Panel (a) shows the 2–8 GHz experimental spectrum of camphorquinone oxime expanded with Ne after co‐adding 500k FIDs (upper trace, in black), and the fitted rotational transitions for the O (blue) and C (orange) isomers. Portions of the experimental spectrum and fitted rotational transitions for the C (panel b) and O (panel d) isomers are shown below. The rotational transitions are identified by the corresponding rotational quantum numbers as $J_{K_{a}K_{c}F}\leftarrow J_{K_{a}^{\prime }K_{c}^{\prime }F^{\prime }}^{\prime }$, where J is the rotational angular momentum quantum number, $K_{a}$ and $K_{c}$ are the projections of J onto the principal axes at the prolate and oblate symmetric top limits, respectively. F is the total angular momentum quantum number, which includes the nuclear spin, I(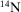) = 1. The molecular structures of C, O, and of the corresponding transition state are illustrated in panel (c), as determined by DLPNO‐SCS‐MP2/def2‐TZVP. The experimental positions of the carbon atoms obtained by the Kraitchman analysis are represented by the purple spheres.

In this work, we use MRR spectroscopy to study camphorquinone oxime and its micro‐hydrated forms in the gas phase. This prototypical molecular switch comprises a rigid camphorquinone structure and a functional oxime group that yields two low energy topologies as observed in Figure 1 panel c: a closed form, C, with an intramolecular NOH⋯O bond, and an open form, O, where the oxime and carbonyl groups are disengaged. This system is thus a good prototype to study the functionality of oxime groups. We found that, although this is a relatively simple molecular system, many levels of theory recommended for organic molecules failed to predict O as the most stable topology, requiring an extensive benchmark study to settle the energy ordering dispute. We also investigated the stepwise growth of the hydrated complexes up to ($H_{2}O$)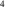. Although quantum chemistry calculations confirm an almost barrierless landscape, isotopic data on 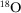 species reveals a scenario that is most compatible with vibrationally averaged structures. A closer look at the water–solute interactions uncovers that the secondary interactions are not sufficient to restrict the dangling of most water molecules in the observed complexes. Our findings support the existence of concerted motions within the water network that solvates the functional site, and we discuss the role of the oxime moiety in defining the interacting water topologies.

## Results and Discussion

**Figure 2:**
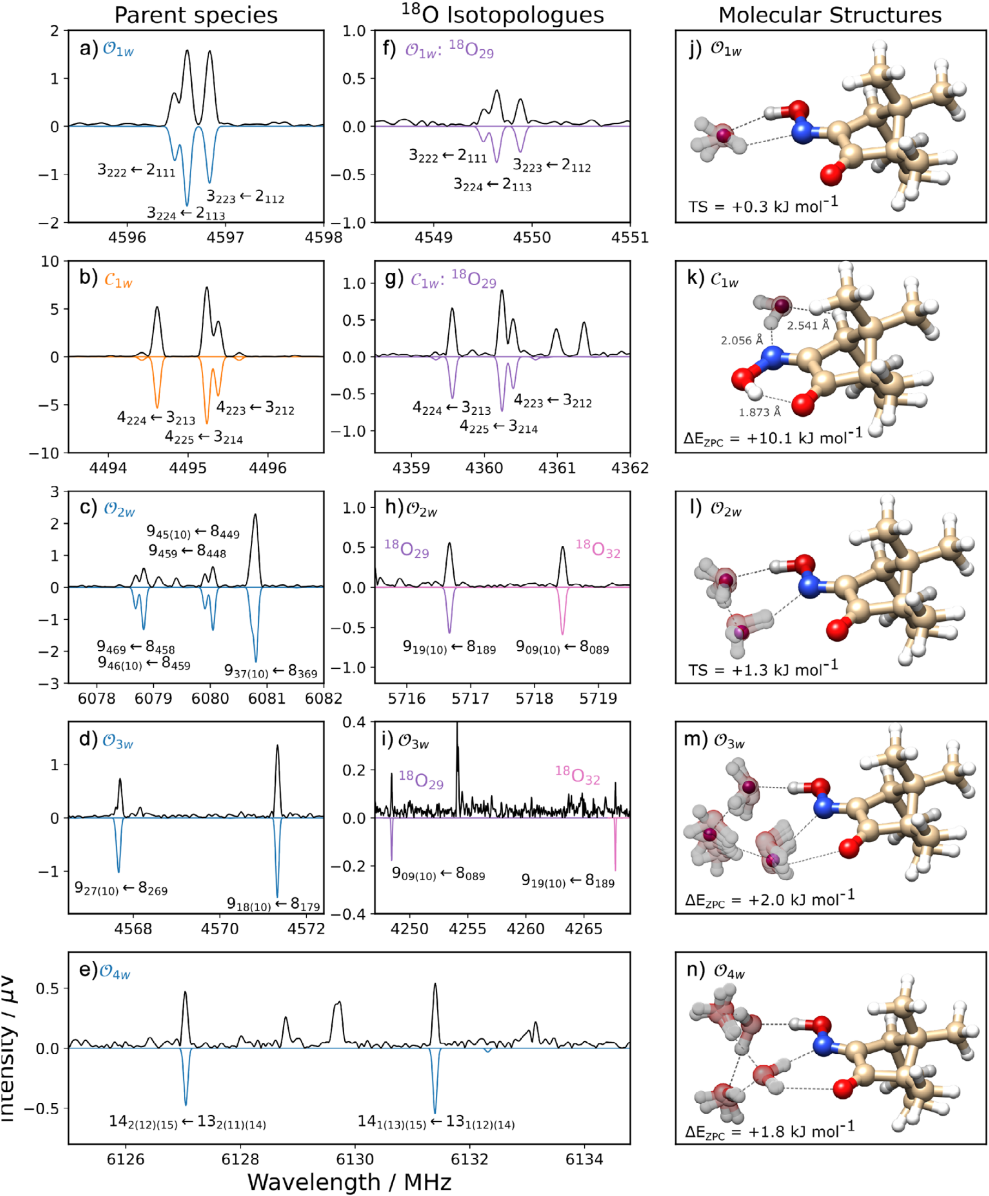
Rotational spectra and structures of the hydrated switch forms. Panels (a)–(e) show portions of the rotational spectrum for the parent 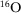 species, whereas panels (f)–(i) correspond to the 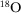 isotopologues. The black upper trace of panels (a)–(i) represents the experimental spectrum, whereas the fitted rotational transitions are shown below. The rotational transitions are identified by the corresponding rotational quantum numbers as $J_{K_{a}K_{c}F}\leftarrow J_{K_{a}^{\prime }K_{c}^{\prime }F^{\prime }}^{\prime }$, where J is the rotational angular momentum quantum number, $K_{a}$ and $K_{c}$ are the projections of J onto the principal axes at the prolate and oblate symmetric top limits, and F is the total angular momentum quantum number, which includes the nuclear spin, I(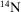) = 1. The molecular structures of these species are shown in panels (j)–(n), as determined by DLPNO‐SCS‐MP2/def2‐TZVP, where the purple spheres represent the position of the oxygen atoms, as determined by the Kraitchman equations. The atomic distances are only shown for $C_{1w}$, since it is the only structure that presents no evidence of dynamic motion of the water network.

The black upper traces in Figure 1 (panels a, b, d) and Figure 2 (panels a–i) show the experimental rotational spectra. The lower traces correspond to the fitted simulated spectra.^[^ ^41^, ^42^ ^]^ The molecular structures of the identified monomers are shown in Figure 1 panel c, whereas the corresponding hydrated species are shown in Figure 2 panels j–n.

Tables 1 and 3 show the experimental rotational constants, quartic centrifugal distortion constants, and the nuclear quadrupole coupling constants for the identified species. The predicted rotational constants, dipole moment components, and the theoretical relative energy are also shown in Table 1.

**Table 1:** Experimental and calculated spectroscopic constants for O and C. The A,B,C parameters are the rotational constants; $\Delta_{J},\Delta_{JK},\Delta_{K},\delta_{J},\delta_{K}$, the quartic centrifugal distortion constants; and $\chi_{aa},\chi_{bb}-\chi_{cc}$, the nuclear quadrupole coupling constants associated to the atom. The predicted dipole moment components, μ, for the a‐, b‐, and c‐type transitions, and the corresponding number of assigned lines is shown in parentheses. The standard error of each fit, σ, and the predicted zero‐point corrected relative energy, $\Delta E_{\text{ZPC}}$ are also shown. All theoretical parameters are expressed in the *principal axis system* (PAS).

	Open switch (O)			Closed switch (C)		
Constants	Experimental	B3LYP‐D3BJ	DLPNO‐SCS‐MP2	Experimental	B3LYP‐D3BJ	DLPNO‐SCS‐MP2
A (MHz)	1150.15866(26)	1152	1153	1239.37010(83)	1245	1241
B (MHz)	762.97776(25)	763	765	735.20116(32)	736	737
C (MHz)	661.07205(26)	661	662	669.62287(34)	671	670
$\Delta_{J}$ (kHz)	0.0259(48)	–	–	–	–	–
$\Delta_{JK}$ (kHz)	–	–	–	0.127(38)	–	–
$\chi_{aa}$ (MHz)	−0.5765(49)	−0.8	−0.9	1.2640(46)	1.2	1.1
$\chi_{bb}-\chi_{cc}$ (MHz)	−2.0674(93)	−2.5	−3.6	−5.5841(85)	−6.7	−8.3
$\mu_{a}$ (D)	yes (36)	0.9	1.0	yes (70)	−4.7	−4.8
$\mu_{b}$ (D)	yes (106)	−3.4	3.2	no	0.2	0.0
$\mu_{c}$ (D)	yes (9)	0.5	0.4	yes (6)	0.5	0.5
σ (kHz)	9.74	–	–	7.48	–	–
$\Delta E_{\text{ZPC}}$ (kJ ${\mathrm{mol}}^{-1}$)	–	+ 2.2	0	–	0	+ 3.1

Besides camphorquinone‐based species, the experimental spectrum also contained the rotational signatures of related synthesis reactants^[^ ^43^ ^]^ and degradation products like camphor,^[^ ^44^ ^]^ and its hydrated forms (1w(I), 1w(II), 2w(I)),^[^ ^45^ ^]^ and of camphorquinone. The rotational constants of camphorquinone are not reported in the literature, but its spectra could be easily fitted due to its strong $\mu_{a}$ dipole component, giving A = 1241.4540(56) MHz, B = 1014.85006(50) MHz, C = 889.87546(41) MHz. Since this species is out of the scope of the present paper, it will not be discussed further, but additional information can be found in Section 5 of the Supporting Information.

### Open and Closed Switch Forms

The experimental rotational spectrum shown in Figure 1 is clearly dominated by two species, corresponding to the open (O) and closed (C) isomers of the camphorquinone oxime switch, for which the experimentally derived abundances reflect a ratio of 72(13)%:28.0(48)% (O:C). As seen in Table 1, both DFT and perturbation theory predict the spectroscopic constants of these species within 1% accuracy of the experimental values. The predicted nuclear quadrupole coupling constants are not as good, but present the typical deviations from the experimental values, being better predicted by the B3LYP‐D3BJ/def2‐TZVP level of theory. The signal‐to‐noise ratio of our spectrum enabled us to obtain the satellite spectra of the 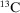 isotopologues in natural abundance (1.1%) for the closed switch topology, allowing us to obtain the experimental positions of the carbon atoms, as shown in Figure 1 Panel c. The corresponding rotational constants and transition frequencies are presented in the Suppporting Information. This analysis could not be performed for the open switch form, since it yields less intense rotational transitions due to weaker dipole moment components than C, preventing the assignment of the 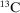 satellite spectra. Other variations of rotations involving the oxime group were considered in our calculations, but had relative energies above 10 kJ ${\mathrm{mol}}^{-1}$, and were not observed in the experimental spectrum.

In the case of the C form, Figure 1 (panel c), the oxime's C═N─O angle (115.9) allows the formation of an intramolecular bond (1.884 Å) between the quinone O atom and the oxime H atom. In the O form, the oxime's C═N─O angle increases to 249.2, separating the O and H atoms and breaking their intramolecular NOH⋯O bond. We were able to observe both isomers in all measurements, including those performed with Ar, suggesting that there is no isomerization through relaxation in the supersonic jet. To calculate the ground‐state energy barrier between the C and O forms, we performed a nudged elastic band (NEB) calculation followed by a transition state optimization. The resulting minimum energy path (MEP) for the ground‐state isomerization is an in‐plane inversion, in accordance with former studies on imine‐ and oxime‐based systems.^[^ ^10^, ^11^, ^12^, ^13^ ^]^ The transition state for the ground‐state isomerization path, shown in Figure 1, yields an energy barrier above 200 kJ ${\mathrm{mol}}^{-1}$, which is compatible with the presence of both isomers in the experimental spectra and with literature values for similar systems.^[^ ^10^, ^11^, ^12^ ^]^

As shown in Table 1, the first calculations made at the popular B3LYP‐D3BJ/def2‐TZVP level of theory indicate that the closed switch topology, C, is more stable than the open form, O, by 2.2 kJ ${\mathrm{mol}}^{-1}$. The experimental relative abundances were obtained from the amplitude of the predicted dipole moments and from the line intensities of all assigned rotational transitions. This analysis revealed a ratio of O/C=2.55, for measurements with Ne. Changing the carrier gas to He and Ar confirmed that the O form was significantly more abundant than C, yielding a similar value for O/C, as shown in Table S39. Confronted by these disagreements between theory and experiment, we performed single‐point energy (SPE) calculations of the optimized C and O geometries using the DLPNO‐CCSD(T)/aug‐cc‐pVTZ^[^ ^46^ ^]^ level of theory. Interestingly, this higher level of theory appointed O as the most stable topology by 0.7 kJ ${\mathrm{mol}}^{-1}$ without zero‐point correction, contradicting the popular B3LYP‐D3BJ^[^ ^47^, ^48^, ^49^, ^50^ ^]^ method, but in agreement with the experimental results.

To further investigate this issue, we repeated the calculation with a larger basis set, aug‐cc‐pVTZ, a larger integration grid, defgrid3, and extreme converge thresholds. Although these modifications decreased the energy difference between both isomers to 0.5 kJ ${\mathrm{mol}}^{-1}$, the energy ordering of the O and C forms remained the same. We also evaluated this system at the B3LYP level of theory using NWChem,^[^ ^51^ ^]^ but the results still indicated C as the most stable switch form by 1.9 kJ ${\mathrm{mol}}^{-1}$, similarly to what is obtained with ORCA.^[^ ^52^, ^53^ ^]^ This suggests that the issues with energy prediction could be intrinsic to the B3LYP level of theory, not with its implementation across different software packages.

Inaccuracies in energy determination with B3LYP have been reported before, but the fact that such a popular method failed to predict the correct energy ordering of these two simple isomers raises some concerns. These unexpected results led us to benchmark several levels of theory, Table 2, with the goal of finding a suitable method to capture the correct energy ordering of the O and C forms. It turns out that B3LYP is not the only level of theory threatened by this simple molecular system; popular functionals thoroughly validated by large benchmarking studies^[^ ^54^, ^55^ ^]^ like B3PW, PBE0,^[^ ^56^, ^57^ ^]^ REVPBE38, TPSSh,^[^ ^58^, ^59^ ^]^ and even the high‐level DLPNO‐MP2^[^ ^60^ ^]^ method fail to predict the correct energy ordering of O and C. M062X^[^ ^61^ ^]^ and PBEh‐3c were successful at predicting the correct energy order of O and C but the zero‐point corrected relative energies are not in complete agreement with CCSD results nor with the experimental observations; whereas M062X slightly underestimates the relative energy by predicting it to be 0.4 kJ ${\mathrm{mol}}^{-1}$, PBEh‐3c clearly overestimates it by predicting a 8.5 kJ ${\mathrm{mol}}^{-1}$ difference. The most reasonable result was obtained by implementing the Grimme's spin‐component scaled (SCS) DLPNO‐MP2,^[^ ^60^, ^62^ ^]^ which yielded an energy difference of +3.1 kJ ${\mathrm{mol}}^{-1}$ of C relatively to O. We have repeated these calculations without dispersion correction to the DFT methods, but the energy ordering was not affected. More details about these benchmarking studies are given in Table S2.

**Table 2:** Zero‐point corrected relative energies, $\Delta E_{\text{ZPC}}$, and relative Gibbs free energy at 383.15 K, $\Delta G_{383.15}$, in kJ ${\mathrm{mol}}^{-1}$ obtained for the Closed (C) and Open (O) switch forms, at different levels of theory. We used the def2‐TZVP basis set for all methods, and the RIJCOSX approximation for all DFT methods.

Energy / kJ ${\mathrm{mol}}^{-1}$ →	$\Delta E_{\text{ZPC}}$		$\Delta G_{383.15}$	
Level of theory ↓	C	O	C	O
TPSSh‐D3BJ	0	+5.4	0	+3.9
B3PW‐D3BJ	0	+4.6	0	+3.3
PBE0‐D3BJ	0	+4.6	0	+3.2
REVPBE38‐D3BJ	0	+2.5	0	+1.2
B3LYP‐D3BJ	0	+2.2	0	+0.8
DLPNO‐MP2	0	+0.9	+0.3	0
M062X	+0.4	0	+1.3	0
DLPNO‐SCS‐MP2	+3.1	0	+4.1	0
PBEh‐3c	+8.5	0	+9.1	0

Given that the C form is considerably more compact than O, the contribution of entropy may play a significant role in their energy ordering. To evaluate this, we calculated the relative Gibbs free energy at 383.15 K, $\Delta G_{383.15}$, the temperature at which the sample was heated inside the nozzle. As shown in Table 2, the energy ordering based on ΔG is maintained for almost all levels of theory. The exception is DLPNO‐MP2, which now predicts O as the most stable switch form, in agreement with experimental observations.

There is a significant amount of methods that do not capture the correct energy ordering of both switch forms with neither ΔG or $\Delta E_{\text{ZPC}}$. Considering that the energetics of a camphorquinone imine molecular switch^[^ ^18^ ^]^ were correctly predicted by theory, we suspect that the energy‐ordering issue lies on modeling the open/closed oxime–quinone topologies. We could not find any helpful information in the literature to guide our benchmarking studies, and had to repeat geometry optimization and frequency calculations at different levels of theory, guided solely by trial‐and‐error. These activities significantly delayed our studies and required a large amount of computational resources. In the end, we elected DLPNO‐SCS‐MP2/def2‐TZVP as the best level of theory to model this molecular system, since it is able to successful capture the molecular structure and energy ordering of both isomers with $\Delta E_{\text{ZPC}}$ and ΔG, especially since the experimental abundance of O:C yields $\Delta G_{383.15}∼$ 3 kJ ${\mathrm{mol}}^{-1}$. Thus, all subsequent calculations of micro‐hydrated structures were refined at this level of theory. Nevertheless, these findings call for future and more detailed theoretical studies focused on the oxime functional group in order to improve the performance of current theory levels. This case study is thus a fresh addition to the short, yet relevant list of examples of molecular systems where high‐resolution spectroscopic techniques arise as fundamental tools to highlight the limitations in state‐of‐the‐art theoretical methods.^[^ ^63^ ^]^

### Concerted Dynamics of Water Molecules

To unveil the stepwise growth of the water network around camphorquinone oxime, we performed a separate measurement where a small amount of $H_{2}^{16}O$ was added to the Ne gas line. In Figure 2 (panels a–e) we show portions of the corresponding rotational spectrum. Although the $O_{1w}$ is the lowest energy form, spectral signatures of a closed monohydrate could also be tracked and assigned to the $C_{1w}$.

The conformational search^[^ ^64^ ^]^ for micro‐hydrated species promptly suggests the existence of dynamics involving the water network at the open switch moiety. After geometry optimization at the DLPNO‐SCS‐MP2/def2‐TZVP level of theory, several energy minima and transition states were found within a few kJ ${\mathrm{mol}}^{-1}$ with very similar rotational constants. For each micro‐hydration order, all of these low‐energy geometries turn out to be very similar, differing only in the water O lone pairs that establish intermolecular O⋯H bonds. A closer look at the experimental spectrum reveals that despite the multiple predicted equilibrium structures, there is a single species present in the spectrum per micro‐hydrated order, as shown in Table 3 and in Table S40.

**Table 3:** Experimental spectroscopic constants for the observed micro‐hydrated species. The A,B,C parameters are the rotational constants; $\Delta_{J},\Delta_{JK},\delta_{J},\delta_{K}$ are the quartic centrifugal distortion constants; and $\chi_{aa},\chi_{bb}-\chi_{cc}$ are the nuclear quadrupole coupling constants associated to the atom. The number of assigned lines for each transition type is shown in parentheses. The standard error for each fit, σ, and the theoretical zero‐point corrected relative energy (DLPNO‐SCS‐MP2/def2‐TZVP), $\Delta E_{\text{ZPC}}$ are also shown.

Constants	$O_{1w}$	$C_{1w}$	$O_{2w}$	$O_{3w}$	$O_{4w}$
A (MHz)	1118.05985(48)	878.82397(26)	960.88481(54)	846.3302(25)	715.902(49)
B (MHz)	455.52043(19)	597.71561(28)	357.10552(30)	263.67614(17)	222.13527(20)
C (MHz)	414.16275(21)	468.60872(26)	316.23806(26)	233.53832(17)	212.28583(19)
$\Delta_{J}$ (kHz)	0.0167(16)	0.0918(27)	0.0213(12)	0.01265(35)	0.01088(40)
$\Delta_{JK}$ (kHz)	0.1489(80)	–	–	0.0161(80)	–
$\delta_{J}$ (kHz)	–	0.0307(15)	0.00470(59)	–	–
$\delta_{K}$ (kHz)	–	–	0.227(57)	–	0.144(75)
$\chi_{aa}$ (MHz)	−2.246(12)	−4.3136(47)	−2.845(25)	−3.097(80)	–
$\chi_{bb}-\chi_{cc}$ (MHz)	−0.828(16)	0.4685(80)	−0.721(21)	0.42(26)	–
$\mu_{a}$ (D)	yes (45)	yes (149)	yes (51)	yes (102)	yes (155)
$\mu_{b}$ (D)	yes (97)	yes (60)	yes (93)	yes (4)	no
$\mu_{c}$ (D)	no	no	no	no	no
σ (kHz)	9.39	10.45	10.34	9.63	11.63
$\Delta E_{\text{ZPC}}$ (kJ ${\mathrm{mol}}^{-1}$)	0	+10.1	0	0	0

Subsequent measurements with $H_{2}^{18}O$, Figure 2 panels f–i, enabled the identification of all singly substituted 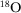 species up to the third order of micro‐hydration. Through the Kraitchman equations,^[^ ^39^ ^]^ we verified that the O atoms of the water molecules were located at intermediate positions between the predicted equilibrium structures, mostly compatible with transition state geometries, as evidenced in Figure 2 panels j–m. Additional information on the 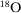 isotopologue structures can be found in the Supporting Information.

The mono‐hydrated open switch, $O_{1w}$, has two very similar equilibrium structures within 0.2 kJ ${\mathrm{mol}}^{-1}$, as seen in Table S40. The difference lies in the orientation of the dangling H atom of the water molecule, which is either pointing upward or downward relative to the plane defined by the oxime group, Figure 2 panel j. Through a NEB analysis, we found a low‐energy (+0.3 kJ ${\mathrm{mol}}^{-1}$) transition state at the point where this H atom crosses the plane of the oxime moiety. Although the H atom is the main actor of this motion, the position of the O atom of the water molecule also shifts between the two equilibrium structures. In fact, the experimental position of this O atom is determined in‐between these minima, as shown in Table S42. We also highlight that the magnitude of the c dipole moment component predicted for the equilibrium structures (1.3 D and ‐1.1 D) should allow the observation of type‐c lines. The absence of these transitions in the spectrum suggests that the observed structure is more compatible with the transition state geometry, which yields $\mu_{c}=$ 0.2 D. All this evidence points to an observable that is actually a vibrationally averaged structure, as a direct consequence of the water's large‐amplitude motion (LAM) allowed by the lack of secondary interactions.

Although the mono‐hydrated closed switch, $C_{1w}$, has an energy of 10.1 kJ ${\mathrm{mol}}^{-1}$ above $O_{1w}$, this species was also present in the experimental spectrum. With the switch closed, the intramolecular NOH⋯O bond prevents the water molecule from binding simultaneously to the oxime H and N atoms, as it does in $O_{1w}$. Facing this limitation, the most favorable docking station for the water molecule is located on the opposite side of the N atom. The water molecule is found on the oxime plane, establishing an intermolecular OH⋯N bond with the closed switch, as seen in Figure 2 panel k. Contrary to the hydrated open switch, we did not find other low‐energy equilibrium states with similar rotational constants for the mono‐hydrated closed topology. Additionally, the experimental position of the O atom was compatible with the geometry of the global minimum, with a maximum deviation of 0.0101 Å, as seen in Table S42. Thus, contrary to $O_{1w}$, and although $C_{1w}$ also has a water molecule that establishes no secondary interactions, we do not find any evidence of micro‐hydration dynamics around the closed switch form. Interestingly, such dynamic effects occur for the closed switch form of a similar molecular system enclosing an imine functional moiety,^[^ ^18^ ^]^ instead of the oxime investigated here. This highlights the sensitivity of micro‐hydration dynamics to minor differences in structural features.

We predicted two low‐energy equilibrium structures for the di‐hydrated open switch form, $O_{2w}$ within 0.5 kJ ${\mathrm{mol}}^{-1}$. In both, the water dimer binds to the OH group and to the N atom of the oxime group, as seen in Figure 2 panel l. The difference between the two equilibrium structures lies with the orientation of the water H atoms, which are found above or below the oxime plane alternately. The O atoms of the two water molecules also shift with this reorientation, but their experimental position is found closer to the midpoint of the two equilibrium structures, as evidenced in Table S51. Although the O coordinates predicted for the equilibrium structures differ as much as 0.813 Å from the experimental ones, the predicted coordinates for the transition state are more in agreement with the experimental observations, with a maximum deviation of 0.0621 Å. This transition state is 1.3 kJ ${\mathrm{mol}}^{-1}$ above the global minimum, low enough to suggest that the observable is a vibrationally averaged structure. Thus, once again, we have reasons to argue in favor of micro‐hydration dynamics around the open switch form but now, with two water molecules at play, the individual dynamics is converted into a concerted LAM.

For the tri‐hydrated open switch, $O_{3w}$, we found six distinct equilibrium structures with similar rotational constants, all within 2.0 kJ ${\mathrm{mol}}^{-1}$, as seen in Table S59. In these geometries, the hydration chain is extended from the oxime group to the O atom on the quinone site, as shown in Figure 2 panel m. The water molecule in the middle of the hydration chain does not interact directly with the oxime switch, being the one that presents a wider set of favorable docking positions; it can be found relatively close to the oxime's plane, or further away in the upward or downward directions, in an almost‐symmetrical way. The position of the other two water molecules also shifts, but within a narrower space, since they are bonded to the H and N atoms of the oxime group. The experimental position of the three O atoms clearly indicates that the water molecules are found in close proximity to the oxime plane. These coordinates are compatible with the global minimum predicted for $O_{3w}$, as seen in Table S60, but also with an intermediate position of the hydration chain for the low‐energy equilibrium geometries. Through a NEB analysis, we mapped the minimum energy paths between the six equilibrium structures, and then optimized the corresponding transition state geometries. The result is, once again an almost‐barrierless landscape, where five transition states are found within 1.4 kJ ${\mathrm{mol}}^{-1}$, as seen in Table S59. Although in this case we do not have evidence to unambiguously claim that we are in the presence of a concerted LAM of the water network, our results are still compatible with the observation of a vibrationally‐averaged structure. This LAM would be a combination of a puckering motion of the water molecules above and below the oxime plane, and of the reorientation of the water H‐atoms. A puckering LAM involving a single water molecule within a larger cluster has been previously reported for the Benzaldehyde‐($H_{2}O$)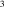 complex, where the oxygen of the middle water molecule shifts as far as 2.02 Å^[^ ^23^ ^]^ via quantum tunnelling through an energy barrier of 9.2 kJ ${\mathrm{mol}}^{-1}$. Contrary to this case, the middle water molecule in the $O_{3w}$ complex is not directly bound to the solute molecule, significantly lowering the puckering energy barrier and allowing the oxygen atom of the middle water molecule to shift as far as 1.67 Å. LAMs involving the reorientation of dangling water H atoms have also been reported in the literature for the hydrated forms of camphorquinone imine switch,^[^ ^18^ ^]^ and for 1‐phenyl‐2,2,2‐trifluoroethanol.^[^ ^19^ ^]^

**Figure 3:**
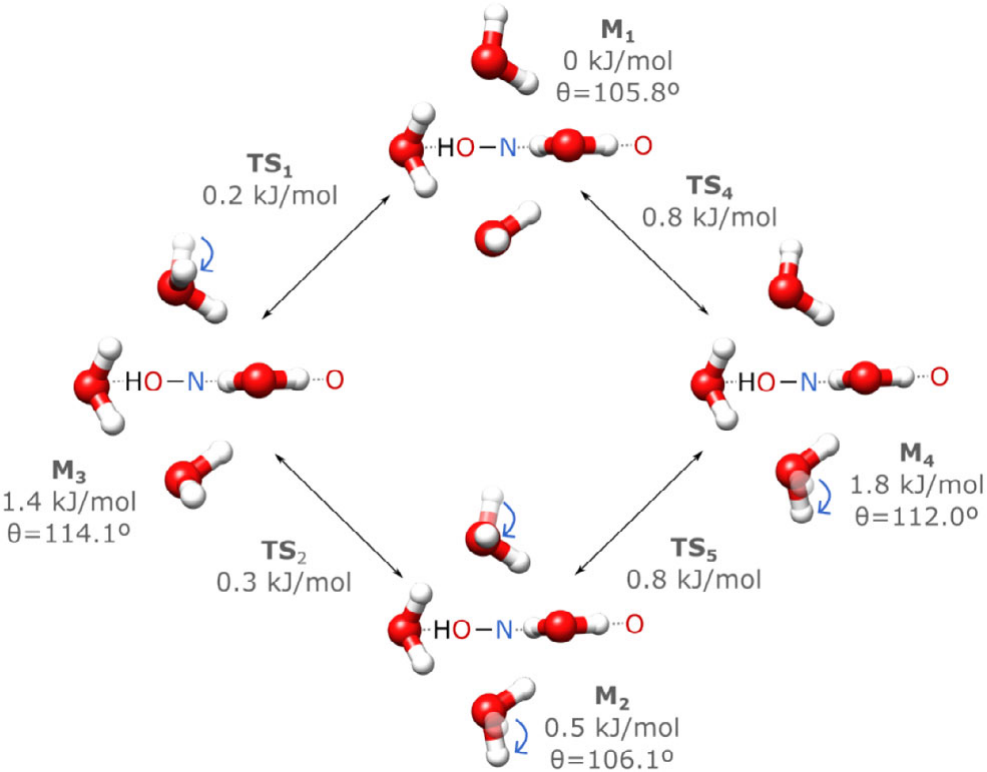
Large amplitude motion of the $O_{4w}$ **complex**. For simplicity, only the water molecules are shown for each minimum structure (M). The arrows and transparencies illustrate the moving water molecules relatively to $M_{1}$ whereas the dotted lines illustrate the docking sites to the oxime group (NOH) and to the oxygen of the carbonyl group. All energies, $\Delta E_{\text{ZPC}}$, are normalized to $M_{1}$, as determined by DLPNO‐SCS‐MP2/def2‐TZVP. The puckering angle of the water complex, θ, is also shown for the four equilibrium structures.

For the complex with four water molecules, $O_{4w}$, we found four equilibrium geometries with similar rotational constants (see Table S76) that are compatible with the experimental observations, all within 1.8 kJ ${\mathrm{mol}}^{-1}$. Panel n of Figure 2 clearly shows that the water tetramer docks perpendicularly to the oxime plane: the two water molecules bound to the oxime switch do not shift position, whereas the other two have a dangling H atom each. The difference between the four equilibrium structures of the $O_{4w}$ complex is further highlighted in Figure 3, where the two dangling water molecules can adopt two favourable orientations each, rendering a total of four possible motifs. Using the same NEB‐based methodology as for $O_{3w}$, we found four distinct transition states within 0.8 kJ ${\mathrm{mol}}^{-1}$ connecting the equilibrium structures that differ in the orientation of a single water molecule. This suggests that these four geometries are connected by independent LAMs of two partially‐unbound water molecules. This independent up ↔ down reorientation is only possible because the two “free” water molecules are separated from one another by the fixed water molecules that interface with the oxime switch.

We successfully resolved the hyperfine splitting for all molecular clusters with the exception of $O_{4w}$. As seen in Table S40, the $\chi_{aa}$ values were much better predicted than the $\chi_{bb}-\chi_{cc}$ constants for all microsolvated structures. This can be a limitation of the level of theory used, or due to the influence of micro‐hydration dynamics on the local electric field gradients as in the case of the 2,2,2‐trifluoroethanol⋯ammonia complex,^[^ ^68^ ^]^ especially since the N atom is a main anchor point for micro‐solvation. Nevertheless, the theoretical quadrupole coupling constants do not seem sensitive to the motion of the water molecules; the magnitudes of $\chi_{aa}$ and $\chi_{bb}-\chi_{cc}$ are very similar for the different equilibrium‐ and transition‐state geometries found per micro‐hydration order. For $O_{4w}$, the line splitting was below the resolution of our spectrometer, preventing us from determining the quadrupole coupling constants.

We note that the B3LYP level of theory also captures the almost barrierless landscape of the micro‐hydrated forms of the open switch, as seen in Table S86. Nevertheless, it still differs from the DLPNO‐SCS‐MP2/def2‐TZVP method when predicting the relative energies, $\Delta E_{\text{ZPC}}$, between $O_{1w}$ and $C_{1w}$. Similarly to the isolated forms, B3LYP is about 5 kJ ${\mathrm{mol}}^{-1}$ off when comparing the relative energies between the open and closed forms of camphorquinone oxime, pointing toward a systematic error in the energy ($\Delta E_{\text{ZPC}}$) calculations between both switch topologies.

### Water–Solute Interactions

**Figure 4:**
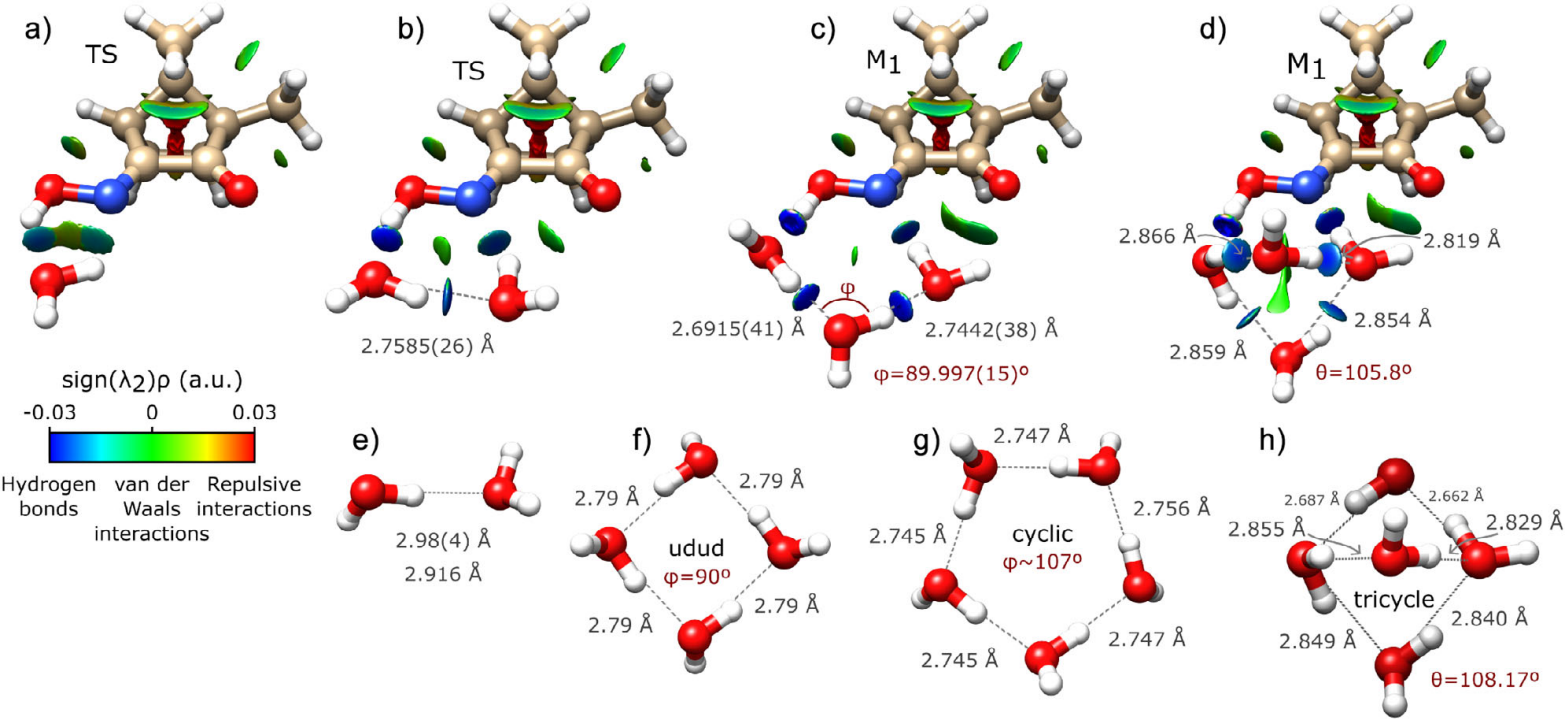
Stepwise growth of the hydration network around the open form of camphorquinone oxime. Panels (a)–(d) show the NCI analysis for the O‐water complexes that are most compatible with the experimental observations. The experimental O⋯O distances obtained through the Kraitchman equations are presented for $O_{1w}$, $O_{2w}$, and $O_{3w}$, whereas for $O_{4w}$ the O⋯O distances where obtained at the DLPNO‐SCS‐MP2/def2‐TZVP level of theory. The angle between consecutive oxygen atoms, ϕ is shown for the $O_{3w}$ complex, whereas the puckering angle θ is shown for $O_{4w}$. Panels (e)–(h) present the pure water complexes reported in the literature.^[^ ^21^, ^28^, ^32^, ^65^, ^66^, ^67^ ^]^

Panels a–d of Figure 4 show the NCI analysis^[^ ^69^, ^70^, ^71^, ^72^, ^73^ ^]^ of the O‐water clusters. Only a single geometry is shown per hydration order, since the results are similar for the different equilibrium structures that compose the LAM. Panels e–h illustrate relevant pure water complexes reported in the literature.^[^ ^21^, ^28^, ^32^, ^65^, ^66^, ^67^ ^]^ By comparison, the influence of camphorquinone oxime in the pure water complexes becomes evident: it affects not only the O⋯O distances, but also the overall arrangement of the water network.

The NCI analysis of the $O_{1w}$ complex already gives some insight to the hydrophilic properties of the oxime group. Figure 4 clearly shows that a single water molecule interacts simultaneously with the OH group and with the N atom through H‐bonds. This is not surprising since the oxime unit comprises a proton donor (O–H end‐group) and a proton acceptor (N atom) site, exhibiting a dual role toward H‐bonds similarly to individual water molecules.

In the $O_{2w}$ complex, the oxime group is well integrated in the hydration network due to its ability to act simultaneously as a proton donor and as an acceptor. To bind to these two complementary neighboring docking stations, the O⋯O cooperativity of the water dimer is significantly increased, as the distance between the water O⋯O atoms is shortened from 2.98(4)^[^ ^28^ ^]^ to 2.7585(26) Å. Additionally, the NCI analysis shown in Figure 4 panel b shows that the water molecule bound to the N atom establishes a weak secondary interaction with the carbonyl group.

In $O_{3w}$, the oxime group is also well integrated in the hydration network, actively participating in the H‐bond chain. As seen in Figure 4 panel c, the overall structure of the resulting oxime–water ring closely resembles the pure cyclic water clusters, but with increased water cooperativity. In fact, the angle between the three consecutive water O atoms is $\phi =89.997{\left(15\right)}^{\circ }$, the same as the pure water tetramer. This is yet another clear indication that the oxime moiety perfectly mimics the behavior of a single water molecule. The interaction between the water network and the carbonyl group is also strengthened relatively to the $O_{2w}$ complex, showing that camphorquinone oxime comprises three hydrophilic docking stations in very close proximity. Nevertheless, these three docking sites are not enough to fully constrict the position of the water molecules, as discussed in the previous section.

With the inclusion of a fourth water molecule, the $O_{4w}$ complex is converted into a 3D structure that differs significantly from the pure cyclic water clusters. As a result, although the O⋯O distances (∼ 2.8–2.9 Å) are similar to the pure water tetramer (2.79 Å^[^ ^65^ ^]^), the planar four‐water ring adapts to the rigid oxime site, bending into a 3‐dimensional book‐like structure with puckering angles ranging from $\phi =105.8^{\circ }$ to $\phi =114.1^{\circ }$, as seen in Figure 3. The oxime group is also responsible for altering the intrinsic proton‐donor/acceptor arrangement of the pure water tetramer; the water molecule bound to the oxime H atom now acts as a double proton donor, whereas the water molecule bound to the oxime N atom acts as a double proton acceptor. This is a significant deviation from the H‐bond arrangement of the most stable pure water clusters, where individual water molecules only start to act as double proton donors or acceptors in complexes with more than six water molecules.^[^ ^21^, ^22^ ^]^ The geometry of the oxime–water complex resembles the tricycle topology of the pure water pentamer, predicted to be 4.6 kJ ${\mathrm{mol}}^{-1}$ above the planar cyclic pentamer.^[^ ^67^ ^]^ Figure 4 showcases the bond distances and puckering angle of the $O_{4w}$, panel d, and of the pure tricycle pentamer, panel h, which are identical and well within the calculation error. This is, once again, a clear indication that the oxime group mimics the behavior of a single water molecule, but because it lacks the necessary flexibility to allow the formation of the most stable water pentamer, the system adopts the geometry of the second most stable pure water complex—the tricycle pentamer. This is not the first system where the water network mimics this tricyclic topology; a similar exotic perturbation to the pure water tetramer was observed for fenchone–($H_{2}O$)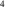 complexes, where a puckered water ring of 127(2) attaches to its carbonyl group.^[^ ^35^ ^]^

Overall, the O–water complexes clearly show that a free N–O–H chain is a highly favorable anchor, performing the role of a proton‐donor at the OH site, and of a proton‐acceptor at the N atom. The close proximity of two key H‐binding sites in the N–O–H group constrains the H‐bonds of the water network, making the open form of camphorquinone oxime a solute that acts as a single water molecule that is constrained in space, significantly reshaping the pure water complexes. The closed switch form, on the contrary, redirects one of the key proton‐donor sites to an intramolecular bond, closing down the OH bridge from the surrounding water molecules. With a single favorable docking site available at the N atom for micro‐solvation, the bigger C–water complexes become energetically unfavorable and thus experimentally unobservable.

In all observed hydrated structures, the water network binds solely with the oxime and carbonyl groups, without interacting with the rest of the camphor moiety. Theory predicts that this behavior extends to larger clusters; even with seven water molecules, the hydration network is constrained to the surroundings of these two docking sites, adopting an independent 3D structure without enveloping the rest of the switch. Theoretical predictions for the $O_{5w}$, $O_{6w}$, and $O_{7w}$ species are provided in Section 4 of the Supporting Information.

## Conclusion

Theoretical predictions on camphorquinone oxime pinpointed significant disagreements between some of the most popular levels of theory commonly used to model simple organic molecules. B3LYP, TPSSh, B3PW, PBE0, and REVPBE38 predict the closed switch form as the most stable one based on both $\Delta E_{\text{ZPC}}$ and ΔG calculations. For this system, M062X, PBEh‐3c, and DLPNO‐SCS‐MP2 are able to consistently predict the open switch form as the most stable one, in agreement with the experimental results obtained by MRR spectroscopy. We hope this preliminary benchmark study triggers more detailed investigations on how different levels of theory are successful at modeling similar molecular systems, and what are the limitations of those that fail. Pinpointing key molecular arrangements that compromise accurate energy predictions using a specific level of theory may significantly aid future investigations,^[^ ^63^ ^]^ especially in cases where experiment cannot easily identify such mispredictions.

The open form of hydrated camphorquinone oxime is also the most energetically favorable since the oxime functional group acts as the main anchor‐point for micro‐solvation. Water molecules establish intermolecular interactions via H‐bonds with the available N and OH sites which act as a proton‐acceptor/donor, respectively, mimicking the behavior of a single water molecule. The rigidity of the NOH group compels the water complexes to adapt to the oxime moiety, significantly reshaping their typical geometry, especially for complexes with more than two water molecules; the oxime–($H_{2}O$)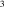 complex resembles the planar water tetramer, whereas the oxime–($H_{2}O$)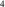 complex adopts the high‐energy topology of the tricycle water pentamer. Since the closed switch form has a single docking site available for micro‐hydration, only the smallest water complex was observed in the MRR spectrum, with a relative energy of 10 kJ ${\mathrm{mol}}^{-1}$ above the analogous open form.

Micro‐hydration dynamics arises from partially‐unbound water molecules around the open switch form, triggering an almost barrierless LAM of the water network; we report concerted LAMs for the two‐ and three‐water complexes, and independent LAMs for the complexes with one and four water molecules. The predicted water reorientation motions above and below the oxime plane resemble those reported for pure water clusters, which have been intensively discussed.^[^ ^65^, ^74^, ^75^, ^76^, ^77^, ^78^, ^79^ ^]^ The lack of tunneling splitting patterns in the rotational spectra is evident, and it supports the broken symmetry of the corresponding potential wells by the presence of the camphorquinone oxime chiral subspace. Moreover, we find that the oxime moiety is also responsible for lowering the energy barriers interconnecting the different isomers due to its highly cooperative integration with the water network. Evidence of flexible water networks have been reported before for other micro‐hydrated systems,^[^ ^18^, ^19^, ^23^ ^]^ suggesting that this may be a more general feature of micro‐hydrated topologies.

Molecular systems that are agile at integrating and rearranging the water network may be strong assets to future molecular nanotechnology applications. For instance, the H‐bonds established by water molecules are known to mediate protein folding,^[^ ^80^, ^81^ ^]^ constrain their structure,^[^ ^82^ ^]^ and activate their functionality.^[^ ^83^, ^84^, ^85^ ^]^ Additionally, several key biological processes rely on “water wires” to speed‐up proton and electron transfer by connecting distant donor and acceptor sites.^[^ ^86^, ^87^ ^]^ Examples are proton pumps in ATP synthesis^[^ ^88^, ^89^, ^90^ ^]^ and information transference between non‐touching biomolecules.^[^ ^83^, ^91^ ^]^ This may open the door to new applications of oxime‐based systems; besides their potential to perform nano‐tasks due to their intrinsic molecular motor properties, they may also be explored to facilitate key biological processes through the induction of water dynamics.

## Conflict of Interests

The authors declare no conflict of interest.

## Supporting information

Supporting Information

## Data Availability

The data that support the findings of this study are available in the supplementary material of this article.

## References

[1] W. Viricel , A. Mbarek , J. Leblond , Angew. Chem. Int. Ed. 2015, 54, 12743–12747. 10.1002/anie.201504661 26189870

[2] L. Zhang , V. Marcos , D. A. Leigh , Proc. Natl. Acad. Sci. USA 2018, 115, 9397–9404. 29483259 10.1073/pnas.1712788115 PMC6156679

[3] F. M. Raymo , Adv. Mater. 2002, 14, 401–414.

[4] A. S. Lubbe , Q. Liu , S. J. Smith , J. W. De Vries , J. C. Kistemaker , A. H. De Vries , I. Faustino , Z. Meng , W. Szymanski , A. Herrmann , B. L. Feringa , J. Am. Chem. Soc. 2018, 140, 5069–5076. 29551069 10.1021/jacs.7b09476 PMC5909178

[5] T. Kudernac , N. Ruangsupapichat , M. Parschau , B. Maciá , N. Katsonis , S. R. Harutyunyan , K.‐H. Ernst , B. L. Feringa , Nature 2011, 479, 208–211. 22071765 10.1038/nature10587

[6] L. Greb , A. Eichhöfer , J.‐M. Lehn , Angew. Chem. 2015, 127, 14553–14556. 10.1002/anie.201506691 26449964

[7] S. Kassem , T. van Leeuwen , A. S. Lubbe , M. R. Wilson , B. L. Feringa , D. A. Leigh , Chem. Soc. Rev. 2017, 46, 2592–2621. 28426052 10.1039/c7cs00245a

[8] C. H. Pollok , T. Riesebeck , C. Merten , Angew. Chem. Int. Ed. 2017, 56, 1925–1928. 10.1002/anie.201610918 28078764

[9] L. Greb , J.‐M. Lehn , J. Am. Chem. Soc. 2014, 136, 13114–13117. 25211628 10.1021/ja506034n

[10] F. Blanco , I. Alkorta , J. Elguero , Croat. Chem. Acta 2009, 82, 173–183.

[11] K. Weiss , C. H. Warren , G. Wettermark , J. Am. Chem. Soc. 1971, 93, 4658–4663.

[12] V. V. Matveevskaya , D. I. Pavlov , A. R. Kovrizhina , T. S. Sukhikh , E. H. Sadykov , P. V. Dorovatovskii , V. A. Lazarenko , A. I. Khlebnikov , A. S. Potapov , Pharmaceutics 2023, 15, 1802. 37513989 10.3390/pharmaceutics15071802 PMC10383563

[13] J.‐M. Lehn , Chem. ‐ Eur. J. 2006, 12, 5910–5915. 16800010 10.1002/chem.200600489

[14] M. Li , W. Li , C. Pérez , A. Lesarri , J.‐U. Grabow , Angew. Chem. Int. Ed. 2024, 63, e202404447. 10.1002/anie.202404447 38717939

[15] M. J. Tubergen , C. R. Torok , R. J. Lavrich , J. Chem. Phys. 2003, 119, 8397–8403.

[16] T. A. LeGreve , W. H. James III , T. S. Zwier , J. Phys. Chem. A 2009, 113, 399–410. 19099446 10.1021/jp807031y

[17] C. Pérez , J. C. López , S. Blanco , M. Schnell , J. Phys. Chem. Lett. 2016, 7, 4053–4058. 27676358 10.1021/acs.jpclett.6b01939

[18] N. M. Campos , R. J. C. Roque , P. Pinacho , C. H. Pollok , C. Merten , P. S. P. Silva , M. R. Silva , M. Schnell , S. R. Domingos , Angew. Chem. Int. Ed. 2025, 64, e202506531. 10.1002/anie.202506531 PMC12304875 40619920

[19] C. D. Carlson , J. Ma , M. H. Al‐Jabiri , A. Insausti , Y. Xu , Phys. Chem. Chem. Phys. 2024, 26, 18067–18075. 38895791 10.1039/d4cp01516a

[20] K. Liu , M. Brown , J. Cruzan , R. Saykally , Science 1996, 271, 62–64. 10.1126/science.271.5245.59 11536731

[21] C. Pérez , M. T. Muckle , D. P. Zaleski , N. A. Seifert , B. Temelso , G. C. Shields , Z. Kisiel , B. H. Pate , Science 2012, 336, 897–901. 22605772 10.1126/science.1220574

[22] J. O. Richardson , C. Pérez , S. Lobsiger , A. A. Reid , B. Temelso , G. C. Shields , Z. Kisiel , D. J. Wales , B. H. Pate , S. C. Althorpe , Science 2016, 351, 1310–1313. 26989250 10.1126/science.aae0012

[23] W. Li , C. Pérez , A. L. Steber , M. Schnell , D. Lv , G. Wang , X. Zeng , M. Zhou , J. Am. Chem. Soc. 2023, 145, 4119–4128. 10.1021/jacs.2c11732 36762446

[24] B. Wu , A. S. Hazrah , N. A. Seifert , S. Oswald , W. Jäger , Y. Xu , J. Phys. Chem. A 2021, 125, 10401–10409. 34846154 10.1021/acs.jpca.1c09058

[25] W. Huang , J. Thomas , W. Jäger , Y. Xu , Phys. Chem. Chem. Phys. 2017, 19, 12221–12228. 28451670 10.1039/c7cp01666b

[26] G. G. Brown , B. C. Dian , K. O. Douglass , S. M. Geyer , S. T. Shipman , B. H. Pate , Rev. Sci. Instrum. 2008, 79, 5. 10.1063/1.2919120 18513057

[27] S. R. Domingos , A. Cnossen , W. J. Buma , W. R. Browne , B. L. Feringa , M. Schnell , Angew. Chem. Int. Ed. 2017, 56, 11209–11212. 10.1002/anie.201704221 PMC5599986 28556402

[28] T. R. Dyke , J. Muenter , J. Chem. Phys. 1974, 60, 2929–2930.

[29] C. Pérez , S. Lobsiger , N. A. Seifert , D. P. Zaleski , B. Temelso , G. C. Shields , Z. Kisiel , B. H. Pate , Chem. Phys. Lett. 2013, 571, 1–15.

[30] C. Pérez , D. P. Zaleski , N. A. Seifert , B. Temelso , G. C. Shields , Z. Kisiel , B. H. Pate , Angew. Chem. Int. Ed. 2014, 53, 14368–14372. 10.1002/anie.201407447 25348841

[31] C. Pérez , J. L. Neill , M. T. Muckle , D. P. Zaleski , I. Peña , J. C. Lopez , J. L. Alonso , B. H. Pate , Angew. Chem. 2015, 127, 993–996. 10.1002/anie.201409057 25413278

[32] A. L. Steber , C. Pérez , B. Temelso , G. C. Shields , A. M. Rijs , B. H. Pate , Z. Kisiel , M. Schnell , J. Phys. Chem. Lett. 2017, 8, 5744–5750. 29112436 10.1021/acs.jpclett.7b02695

[33] W. Sun , M. Schnell , Angew. Chem. 2022, 134, e202210819. 10.1002/anie.202210819 PMC10099544 36250281

[34] S. Gruet , C. Pérez , A. L. Steber , M. Schnell , Phys. Chem. Chem. Phys. 2018, 20, 5545–5552. 29204595 10.1039/c7cp06518c

[35] E. Burevschi , M. Chrayteh , S. I. Murugachandran , D. Loru , P. Dréan , M. E. Sanz , J. Am. Chem. Soc. 2024, 146, 10925–10933. 38588470 10.1021/jacs.4c01891 PMC11027134

[36] C. Calabrese , W. Li , G. Prampolini , L. Evangelisti , I. Uriarte , I. Cacelli , S. Melandri , E. J. Cocinero , Angew. Chem. Int. Ed. 2019, 58, 8437–8442. 10.1002/anie.201902753 30997948

[37] A. S. Hazrah , A. Insausti , J. Ma , M. H. Al‐Jabiri , W. Jäger , Y. Xu , Angew. Chem. 2023, 135, e202310610. 10.1002/anie.202310610 37697450

[38] A. L. Steber , B. Temelso , Z. Kisiel , M. Schnell , C. Pérez , Proc. Natl. Acad. Sci. USA 2023, 120, e2214970120. 36802430 10.1073/pnas.2214970120 PMC9992814

[39] J. Kraitchman , Am. J. Phys. 1953, 21, 17–24.

[40] Z. Kisiel , (Eds.: J. Demaison et al.), Spectroscopy from Space, Kluwer Academic Publishers, Dordrecht, 2001, pp. 91–106.

[41] J. K. G. Watson , J. Chem. Phys. 1967, 46, 1935–1949.

[42] C. Western , J. Quant. Spectrosc. Radiat. Transfer 2016, 186, 221–242.

[43] B. E. Love , E. G. Jones , Synth. Commun. 1999, 29, 2831–2840.

[44] Z. Kisiel , O. Desyatnyk , E. Białkowska‐Jaworska , L. Pszczółkowski , Phys. Chem. Chem. Phys. 2003, 5, 820–826.

[45] C. Perez , A. Krin , A. L. Steber , J. C. Lopez , Z. Kisiel , M. Schnell , J. Phys. Chem. Lett. 2016, 7, 154–160. 26689110 10.1021/acs.jpclett.5b02541

[46] G. E. Scuseria , A. C. Scheiner , T. J. Lee , J. E. Rice , H. F. Schaefer , J. Chem. Phys. 1987, 86, 2881–2890.

[47] A. D. Becke , J. Chem. Phys. 1993, 98, 5648–5652.

[48] C. Lee , W. Yang , R. G. Parr , Phys. Rev. B 1988, 37, 785. 10.1103/physrevb.37.785 9944570

[49] S. H. Vosko , L. Wilk , M. Nusair , Can. J. Phys. 1980, 58, 1200–1211.

[50] P. J. Stephens , F. J. Devlin , C. F. Chabalowski , M. J. Frisch , J. Phys. Chem. 1994, 98, 11623–11627.

[51] M. Valiev , E. J. Bylaska , N. Govind , K. Kowalski , T. P. Straatsma , H. J. J. Van Dam , D. Wang , J. Nieplocha , E. Aprà , T. L. Windus , W. A. de Jong , Comput. Phys. Commun. 2010, 181, 1477–1489.

[52] F. Neese , Wiley Interdiscip. Rev.: Comput. Mol. Sci. 2012, 2, 73–78.

[53] F. Neese , Wiley Interdiscip. Rev.: Comput. Mol. Sci. 2022, 12, e1606.

[54] L. Goerigk , A. Hansen , C. Bauer , S. Ehrlich , A. Najibi , S. Grimme , Phys. Chem. Chem. Phys. 2017, 19, 32184–32215. 29110012 10.1039/c7cp04913g

[55] L. Goerigk , S. Grimme , Phys. Chem. Chem. Phys. 2011, 13, 6670–6688. 21384027 10.1039/c0cp02984j

[56] J. P. Perdew , K. Burke , M. Ernzerhof , Phys. Rev. Lett. 1996, 77, 3865. 10062328 10.1103/PhysRevLett.77.3865

[57] S. Grimme , J. G. Brandenburg , C. Bannwarth , A. Hansen , J. Chem. Phys. 2015, 143, 5. 10.1063/1.4927476 26254642

[58] J. Tao , J. P. Perdew , V. N. Staroverov , G. E. Scuseria , Phys. Rev. Lett. 2003, 91, 146401. 14611541 10.1103/PhysRevLett.91.146401

[59] V. N. Staroverov , G. E. Scuseria , J. Tao , J. P. Perdew , J. Chem. Phys. 2003, 119, 12129–12137.

[60] C. Møller , M. S. Plesset , Phys. Rev. 1934, 46, 618.

[61] Y. Zhao , D. G. Truhlar , Theor. Chem. Acc. 2008, 120, 215–241.

[62] S. Grimme , L. Goerigk , R. F. Fink , Wiley Interdiscip. Rev.: Comput. Mol. Sci. 2012, 2, 886–906.

[63] I. Uriarte , A. Insausti , E. J. Cocinero , A. Jabri , I. Kleiner , H. Mouhib , I. Alkorta , J. Phys. Chem. Lett. 2018, 9, 5906–5914. 30234988 10.1021/acs.jpclett.8b02339

[64] S. Grimme , J. Chem. Theory Comput. 2019, 15, 2847–2862. 30943025 10.1021/acs.jctc.9b00143

[65] F. N. Keutsch , R. J. Saykally , Proc. Natl. Acad. Sci. USA 2001, 98, 10533–10540. 11535820 10.1073/pnas.191266498 PMC58500

[66] E. Miliordos , E. Aprà , S. S. Xantheas , J. Chem. Phys. 2013, 139, 11. 10.1063/1.4820448 24070285

[67] M. Mandziuk , J. Mol. Struct. 2019, 1177, 168–176.

[68] J. Thomas , I. Peña , C. D. Carlson , Y. Yang , W. Jäger , Y. Xu , Phys. Chem. Chem. Phys. 2020, 22, 23019–23027. 33043940 10.1039/d0cp03329d

[69] E. R. Johnson , S. Keinan , P. Mori‐Sánchez , J. Contreras‐García , A. J. Cohen , W. Yang , J. Am. Chem. Soc. 2010, 132, 6498–6506. 20394428 10.1021/ja100936w PMC2864795

[70] T. Lu , Q. Chen , Comprehensive Comput. Chem. 2024, 2, 240–264.

[71] T. Lu , F. Chen , J. Comput. Chem. 2012, 33, 580–592. 22162017 10.1002/jcc.22885

[72] T. Lu , J. Chem. Phys. 2024, 161, 8. 10.1063/5.0216272 39189657

[73] E. F. Pettersen , T. D. Goddard , C. C. Huang , G. S. Couch , D. M. Greenblatt , E. C. Meng , T. E. Ferrin , J. Comput. Chem. 2004, 25, 1605–1612. 15264254 10.1002/jcc.20084

[74] M. Mandziuk , Chem. Phys. Lett. 2016, 661, 263–268.

[75] M. Eraković , M. T. Cvitaš , Phys. Chem. Chem. Phys. 2024, 26, 12965–12981. 38634688 10.1039/d4cp00013g

[76] C. Vaillant , D. Wales , S. Althorpe , J. Phys. Chem. Lett. 2019, 10, 7300–7304. 31682130 10.1021/acs.jpclett.9b02951

[77] C. L. Vaillant , M. T. Cvitaš , Phys. Chem. Chem. Phys. 2018, 20, 26809–26813. 30328431 10.1039/c8cp04991b

[78] X. Meng , J. Guo , J. Peng , J. Chen , Z. Wang , J.‐R. Shi , X.‐Z. Li , E.‐G. Wang , Y. Jiang , Nat. Phys. 2015, 11, 235–239.

[79] J. K. Gregory , D. C. Clary , J. Chem. Phys. 1996, 105, 6626–6633.

[80] M. S. Cheung , A. E. García , J. N. Onuchic , Proc. Natl. Acad. Sci. USA 2002, 99, 685–690. 11805324 10.1073/pnas.022387699 PMC117366

[81] G. A. Papoian , J. Ulander , M. P. Eastwood , Z. Luthey‐Schulten , P. G. Wolynes , Proc. Natl. Acad. Sci. USA 2004, 101, 3352–3357. 14988499 10.1073/pnas.0307851100 PMC373465

[82] B. Halle , Philos. Trans. R. Soc. Lond. B Biol. Sci. 2004, 359, 1207–1224. 15306377 10.1098/rstb.2004.1499 PMC1693401

[83] M. Chaplin , Nat. Rev. Mol. Cell Biol. 2006, 7, 861–866. 16955076 10.1038/nrm2021

[84] R. V. Dunn , R. M. Daniel , Philos. Trans. R. Soc. Lond. B Biol. Sci. 2004, 359, 1309–1320. 15306385 10.1098/rstb.2004.1494 PMC1693412

[85] N. Smolin , A. Oleinikova , I. Brovchenko , A. Geiger , R. Winter , J. Phys. Chem. B 2005, 109, 10995–11005. 16852340 10.1021/jp050153e

[86] S. Cukierman , Biochim. Biophys. Acta, Biomembr. 2006, 1757, 876–885. 10.1016/j.bbabio.2005.12.001 16414007

[87] J. Lin , I. A. Balabin , D. N. Beratan , Science 2005, 310, 1311–1313. 16311331 10.1126/science.1118316 PMC3613566

[88] M. J. Buch‐Pedersen , B. P. Pedersen , B. Veierskov , P. Nissen , M. Palmgren , Pflugers Arch. 2009, 457, 573–579. 18458946 10.1007/s00424-008-0503-8

[89] D. P. Briskin , J. B. Hanson , J. Exp. Bot. 1992, 43, 269–289.

[90] R. A. Copeland , S. I. Chan , Annu. Rev. Phys. Chem. 1989, 40, 671–698. 2557045 10.1146/annurev.pc.40.100189.003323

[91] J. Völker , H. H. Klump , K. J. Breslauer , Proc. Natl. Acad. Sci. USA 2001, 98, 7694–7699. 11438725 10.1073/pnas.141221298 PMC35404

