# Supplementary material for: Cold‐Captured Dynamic Hydration Networks in Oxime‐Based Photoswitches: A Theoretical Challenge Uncovered by Rotational Spectroscopy
Source: Angew Chem Int Ed Engl. 2025 Sep 6;64(44):e202513560. doi: 10.1002/anie.202513560 (PMC12559473; doi:10.1002/anie.202513560)
Supplement: Supplementary file 1 — Supporting Information [file ANIE-64-e202513560-s001.pdf]

Supporting Information for: “Cold-captured dynamic hydration networks in oxime-based photoswitches: a theoretical challenge uncovered by rotational spectroscopy”

Rita J.C. Roque[1,*], **Nuno M. Campos**[1], **Marcos Gouveia**[1], **and Sérgio R. Domingos**[1,*]

[1]CFisUC, Department of Physics, University of Coimbra, Rua Larga, 3004-516 Coimbra, Portugal
[*]ritaroque@uc.pt, sergio.domingos@uc.pt

Contents

1 Methods **2**
1.1 Computational Methods 2
1.2 Experimental Methods 2
2 The open and closed switch forms **3**
2.1 Singly-substituted ^{13}C isotopologues 3
2.2 Benchmark results 4
B3LYP D3BJ OPT FREQ def2-TZVP RIJCOSX • B3LYP D3BJ OPT FREQ aug-cc-pVTZ Defgrid3 ExtremeSCF • B3PW D3BJ OPT FREQ def2-TZVP RIJCOSX • PBE0 D3BJ OPT FREQ def2-TZVP RIJCOSX • REVPBE38 D3BJ OPT FREQ def2-TZVP RIJCOSX • TPSSh D3BJ OPT FREQ def2-TZVP RIJCOSX • DLPNO-MP2 def2-TZVP def2-TZVP/C OPT NUMFREQ • M062X OPT FREQ def2-TZVP RIJCOSX • PBEh-3c OPT FREQ def2-TZVP RIJCOSX • DLPNO-SCS-MP2 def2-TZVP def2-TZVP/C OPT NUMFREQ
2.3 Transition frequencies 29
2.4 Relative abundances 39
3 The dynamics of micro-hydrated species **40**
3.1 Singly-substituted ^{18}O isopotologues 41
3.2 Clusters with 1 water molecule 41
Equilibrium and transition state geometries • Transition frequencies
3.3 Clusters with 2 water molecules 58
Equilibrium and transition state geometries • Transition frequencies
3.4 Clusters with 3 water molecules 71
Equilibrium and transition state geometries • Transition frequencies
3.5 Clusters with 4 water molecules 89
Equilibrium and transition state geometries • Transition frequencies
3.6 Benchmark – B3LYP calculations for water clusters 101
4 Large water clusters **102**
5 Camphorquinone **107**
References **108**

1 Methods

1.1 Computational Methods

The starting geometry of possible isomers was directly optimized through quantum chemistry calculations with ORCA 5.0[1]; we evaluated different levels of theory including density functional theory (DFT), exploring the B3LYP[2–5], TPSSh[6,7], B3PW, PBE[8,9] and M06[10] functionals; MP2 perturbation theory[11] with and without the Grimme's spin-component scaled (SCS)[12]; and Coupled Cluster single and double excitation (CCSD)[13] calculations.

Conformational searches on water clusters were carried out using the "NCI mode" of the Conformer Rotamer Ensemble Sampling Tool (CREST)[14], using the GFN2-xTB level of theory and 6 kcal mol^{-1} energy threshold. For each order of hydration, n, the conformational search started from two trial structures, adding n water molecules to the open ($\mathscr{O}$) and closed ($\mathscr{C}$) switch topologies, separately. The lower energy conformers were further refined through DFT calculations, at the B3LYP-D3BJ/def2-TZVP level of theory with the RIJCOSX approximation, followed by an DLPNO-SCS-MP2/def2-TZVP optimization and numerical frequency calculation.

The minimum energy pathway (MEP) between structures of interest was obtained with the nudged-elastic band (NEB-CI) method implemented in ORCA 5.0, at the B3LYP-D3BJ/def2-TZVP level of theory with the RIJCOSX approximation. The corresponding transition states, starting from the "Climbing Image" (CI), were optimized at the same level of theory, followed by an DLPNO-SCS-MP2/def2-TZVP optimization and numerical frequency calculation.

The analysis of Non-Covalent-Interactions (NCI)[15,16] was carried out with MultiWFN[17,18]. Molecular graphics and analyses were performed with UCSF Chimera[19].

The recurring fits to the experimental rotational spectra used the Watson's A-reduced semirigid rotor Hamiltonian[20] in the I^r representation and were conducted using PGOPHER[21]. In order to determine the experimental value of the quartic centrifugal distortion constants, we included them in the fit, one by one and then in combinations. In the end, we only maintained the ones determined with enough significance (taking into account their value and uncertainty), and contributed to the fit by lowering the global standard error for the corresponding fit (σ). The value of the insignificant quartic centrifugal distortion constants was fixed at zero.

The rotational transitions are identified throughout the paper by the corresponding rotational quantum numbers as $J_{K_aK_cF} \leftarrow J'_{K'_aK'_cF'}$, where J is the rotational angular momentum quantum number, K_a and K_c are the projections of J onto the principal axes at the prolate and oblate symmetric top limits, and F is the total angular momentum quantum number, which includes the nuclear spin, I(^{14}N) = 1. The non-diagonal elements of the quadrupole coupling tensor were not determined in our analysis.

The rotational spectra of the singly-substituted isotopic species was estimated using CORSCL. The positions of the water oxygen atoms and of the closed switch carbon atoms were determined using the KRA software, which uses the experimental constants obtained for the corresponding singly-substituted ^{18}O and ^{13}C species, respectively. Both software packages are available at the Programs for ROtational SPEctroscopy (PROSPE) database[22].

The relative abundance of species present in the rotational spectra was obtained with the BrightSpec Broadband Analysis (BBA) Software, which uses the experimental spectrum, fitted spectroscopic constants, and the predicted dipole moments as the input variables. This software matches the simulated spectrum with all experimental rotational lines above a given intensity threshold and then weights their contributions to determine the final abundance of a species. To perform this analysis, we chose a threshold 3x above the noise baseline to maximize the number of rotational lines included in this calculation, while minimizing contributions from the noise. We calculated the uncertainty associated with the population ratio as $3\sigma/\sqrt{n}$, where σ is the standard deviation of the signal intensity (provided by the BBA software), and n is the number of rotational transitions used for the calculation of this quantity. The factor 3 was added to consider the 99.7% confidence interval of the BBA estimate.

The experimental relative abundances $[\mathscr{C}]$ and $[\mathscr{O}]$ were used to calculate the experimental free Gibbs energy using

$$\Delta G = -RT \ln\left(\frac{[\mathscr{C}]}{[\mathscr{O}]}\right) \quad (1)$$

1.2 Experimental Methods

All rotational spectra presented here were obtained with a MRR spectrometer by BrightSpec, which is a Chirped-Pulse Fourier Transform Microwave (CP-FTMW) spectrometer operating with three pulsed valves in the 2 – 8 GHz frequency range. Racemic camphorquinone oxime (>95% purity, purchased from TCI) was introduced into three home-made nozzles and heated to 110ºC to induce the necessary vapour-pressure without sample degradation. The carrier gas (either He, Ne or Ar) flowing through the nozzle had a backing pressure of ∼1 bar before being expanded through a 1 mm diameter orifice into the vacuum chamber, which was kept at $\sim 10^{-4}$ mbar. The supersonic jet was controlled by three independent pulsed solenoid valves (General Valve Series 9) operating at a repetition rate of 5 Hz and an opening time of 1200 μs.

Chirped pulses covering the entire 2–8 GHz frequency range, each with a duration of 4 μs, are produced by an arbitrary waveform generator (Keysight M9502A AWG by Keysight) and then amplified by a travelling wave tube (TWT Model 167S/C by Applied Systems Engineering, Inc., with typical power output of $\sim$ 250 W and a rated gain of $\sim$ 40 dB) amplifier. Approximately 1300 μs after each molecular beam, the horn antenna emits eight of these chirped pulses into the vacuum chamber to rotationally-excite the ultra-cold molecules. The corresponding free induction decay (FID) is collected by a second horn antenna (40 μs acquisition time), amplified by a low-noise amplifier, and finally Fourier-transformed and digitized by an oscilloscope (DPO 70804C 8 GHz, 25 GS/s by Tektronix) to obtain the final rotational spectrum in the frequency domain. Based on the analysis of the rotational spectrum, the sample achieved a rotational temperature of $\sim$1 K prior for all species to being interrogated with the 2–8 GHz microwave pulse.

To induce the formation of clusters, water vapour was added to the carrier gas before reaching the nozzles. To measure ^{18}O singly-substituted species, a mixture of $2(H_2^{16}O)$:$1(H_2^{18}O)$ was used. The ^{18}O enriched water (97% purity) used in these measurements was purchased from Eurisotop.

We note that capturing the MRR spectrum of camphorquinone oxime was not trivial, since this sample requires a more rigorous temperature control compared to typical compounds. Whereas a minimum temperature of $\sim$80 ºC was required to raise the monomer signal above the noise threshold, we witnessed significant sample degradation at $\sim$110 ºC, even below its melting point (154 ºC – 156 ºC). This leaves only a 30º C margin to optimize the signal of the monomers and of the hydrated clusters.

2 The open and closed switch forms

2.1 Singly-substituted ^{13}C isotopologues

Table S1. Experimental spectroscopic constants for the observed singly-substituted ^{13}C species of the closed switch. The A,B,C parameters are the rotational constants; $\Delta_J, \Delta_{JK}, \Delta_K, \delta_J, \delta_K$, the quartic centrifugal distortion constants; and $\chi_{aa}, \chi_{bb} - \chi_{cc}$, the nuclear quadrupole coupling constants associated to the ^{14}N atom. The corresponding number of assigned lines is shown in parentheses. The standard error of each fit, σ is also shown. Fixed parameters are represented within brackets, []. ^{a} coordinates originally calculated as imaginary (due to close proximity with the corresponding axis) that were zeroed out.

Constants	^{13}C(1)	^{13}C(2)	^{13}C(3)	^{13}C(4)	^{13}C(5)
A /MHz	1226.868(59)	1230.612(42)	1237.378(36)	1237.336(45)	1238.349(56)
B /MHz	732.1100(12)	730.97102(84)	734.35020(86)	734.77871(94)	734.0021(11)
C /MHz	667.8168(12)	668.60343(86)	668.42279(98)	668.6582(11)	668.3280(14)
Δ_{JK} /KHz	0.45(15)	0.158(79)	[0.13]	[0.13]	0.10(10)
χ_{aa} /MHz	[1.264]	[1.264]	[1.264]	[1.264]	[1.264]
$\chi_{bb} - \chi_{cc}$ /MHz	[-5.584]	[-5.584]	[-5.584]	[-5.584]	[-5.584]
μ_a /D	yes(22)	yes(23)	yes(19)	yes(23)	yes(24)
μ_b /D	no	no	no	no	no
μ_c /D	no	no	no	no	no
σ /KHz	12.64	8.73	7.93	10.29	11.32
a /Å	0.624(6)	1.052(4)	0.865(5)	0.638(6)	1.061(4)
b /Å	1.262(3)	0.19(2)	0.781(5)	0.827(5)	0.583(7)
c /Å	1.606(3)	1.698(2)	0.22(2)	0^a	0^a
Constants	^{13}C(6)	^{13}C(7)	^{13}C(8)	^{13}C(9)	^{13}C(10)
A /MHz	1233.533(67)	1226.426(44)	1237.629(53)	1225.970(25)	1235.956(65)
B /MHz	735.1753(16)	732.5147(11)	733.27637(98)	729.74604(51)	727.4502(13)
C /MHz	667.8738(20)	663.5768(13)	668.1657(11)	668.88577(58)	662.6810(13)
Δ_{JK} /KHz	[0.13]	[0.13]	0.36(13)	[0.13]	0.16(13)
χ_{aa} /MHz	[1.264]	[1.264]	[1.264]	[1.264]	[1.264]
$\chi_{bb} - \chi_{cc}$ /MHz	[-5.584]	[-5.584]	[-5.584]	[-5.584]	[-5.584]
μ_a /D	yes(15)	yes(17)	yes(23)	yes(18)	yes(22)
μ_b /D	no	no	no	no	no
μ_c /D	no	no	no	no	no
σ /KHz	12.44	9.11	10.97	5.58	12.77
a /Å	0.19(3)	1.585(3)	1.200(4)	0.865(5)	2.653(2)
b /Å	1.395(3)	2.093(2)	0.46(1)	0.27(2)	0.935(5)
c /Å	0^a	0^a	0.608(7)	2.099(2)	0.531(8)

2.2 Benchmark results

Table S2. Summary table of benchmarking studies. The A,B,C parameters are the rotational constants, and $\chi_{aa}, \chi_{bb} - \chi_{cc}$, the nuclear quadrupole coupling constants associated to the ^{14}N atom. The predicted dipole moment components, μ, for the a-, b-, and c-type transitions and zero-point corrected relative energy, ΔE_{ZPC} are also shown. All theoretical parameters are expressed in the *principal axis system* (PAS).

Theory level	Species	A MHz	B MHz	C MHz	μ_a D	μ_b D	μ_c D	χ_{aa} MHz	$\chi_{bb} - \chi_{cc}$ MHz	ΔE_{ZPC} kJ/mol	ΔG kJ/mol
B3LYP	$\mathcal{O}$	1152	763	661	0.9	-3.4	0.5	-0.8	-2.6	+2.2	+0.8
def2-TZVP	$\mathcal{C}$	1245	736	671	-4.7	0.2	0.5	1.2	-6.7	0	0
2.2.1	TS	1214	683	618	-0.7	1.5	0.2	5.3	-13.9	+212.9	+211.9
B3LYP	$\mathcal{O}$	1151	763	662	1.1	-3.4	0.5	-0.7	-2.4	+0.5	
2.2.2	$\mathcal{C}$	1244	737	672	-4.8	0.2	0.5	1.3	-6.5	0	
B3PW	$\mathcal{O}$	1162	769	667	0.8	-3.4	0.4	-0.6	-2.8	+4.6	+3.3
2.2.3	$\mathcal{C}$	1256	743	677	-4.6	0.2	0.5	1.2	-6.7	0	0
PBE0	$\mathcal{O}$	1164	771	668	0.8	-3.4	0.4	-0.6	-3.0	+4.6	+3.2
2.2.4	$\mathcal{C}$	1258	745	679	-4.6	0.2	0.5	1.2	-6.9	0	0
REVPBE38	$\mathcal{O}$	1171	773	671	0.9	-3.4	0.4	-0.6	-3.3	+2.5	+1.2
2.2.5	$\mathcal{C}$	1262	747	681	-4.6	0.2	0.5	1.2	-7.2	0	0
TPSSh	$\mathcal{O}$	1153	765	662	0.8	-3.4	0.4	-0.7	-2.3	+5.4	+3.8
2.2.6	$\mathcal{C}$	1249	738.2	672.8	-4.6	0.2	0.5	1.2	-6.3	0	0
MP2	$\mathcal{O}$	1160	770	666	0.9	-3.2	0.4	-0.9	-3.7	+0.9	0
2.2.7	$\mathcal{C}$	1251	742	675	-4.7	0.0	0.5	1.0	-8.5	0	0.3
MO62X	$\mathcal{O}$	1161	768	666	1.0	-3.3	0.4	-0.8	-3.3	0	0
2.2.8	$\mathcal{C}$	1249	740	674	-4.7	0.1	0.5	1.1	-7.2	+0.4	+1.3
	TS	1223	686	621	-0.9	1.4	0.2	5.0	-13.9	+209.2	+209.7
PBEh-3c	$\mathcal{O}$	1172	769.3	669.6	0.9	-3.4	0.4	-0.6	-3.7	0	0
2.2.9	$\mathcal{C}$	1256.4	743.2	677.2	-4.5	0.2	0.5	1.4	-7.6	+8.5	+9.1
	TS	1233	692	627	-0.8	1.5	0.2	5.0	-14.3	+205.4	+205.7
SCS-MP2	$\mathcal{O}$	1153	765	662	1.0	-3.2	0.4	-0.9	-3.6	0	0
2.2.10	$\mathcal{C}$	1241	737	670	-4.8	0.0	0.5	1.1	-8.3	+3.1	+4.1
	TS	1214	682	617	-0.9	1.2	0.2	4.7	-16.1	+231.2	+231.3

Energy / kJ mol^{-1} → Level of theory ↓	ΔE_{ZPC} with D3BJ correction $\mathcal{C}$	$\mathcal{O}$	ΔE_{ZPC} without D3BJ correction $\mathcal{C}$	$\mathcal{O}$
TPSSh	0	+5.4	0	+4.31
B3PW	0	+4.6	0	+2.96
PBE0	0	+4.6	0	+3.76
REVPBE38	0	+2.5	0	+0.26
B3LYP	0	+2.2	0	+0.48

2.2.1 B3LYP D3BJ OPT FREQ def2-TZVP RIJCOSX

Table S3. Equilibrium geometry of camphorquinone oxime 𝒞 isomer.

Atom	*x* /Å	*y* /Å	*z* /Å
C	-3.25778531060612	-0.21014700436242	1.85586462215577
C	-3.08994197338072	-1.31312221179245	0.77462146606214
C	-2.42550303901681	-0.58190716992770	-0.43800001992448
C	-1.00965505907172	-0.32061729875738	0.04926347819137
C	-1.14241561395862	0.72591884000089	1.09917025076089
C	-2.61513837281264	1.02841161690354	1.18500006772828
C	-2.51948118200062	-1.30636895535010	-1.75936303556972
C	-3.03380402193362	0.86335969525093	-0.31036953603186
C	-2.36794165228438	1.88264465697084	-1.23808913868234
C	-4.54286090455521	0.92086842727372	-0.53906093529755
O	0.02852537525562	-0.84910293435353	-0.30935074990711
N	-0.22332406704573	1.26664240897175	1.80420438678750
O	1.05668643501582	0.84096661174939	1.57598887660524
H	-4.30665603800313	-0.02904163654102	2.08723607950336
H	-2.75575158661027	-0.45833200649194	2.78969265882488
H	-2.47955241711092	-2.14999485497285	1.11471056158533
H	-4.04782856354890	-1.72541554445867	0.45821112152569
H	-2.84982258202662	1.98379383403355	1.64847903727012
H	-2.04331366111647	-0.73723789927594	-2.55897575007931
H	-2.01570375940666	-2.27242402951807	-1.70317108503810
H	-3.56109786431586	-1.48175973198470	-2.03270275181148
H	-1.28103202184290	1.89722042605393	-1.14879954846633
H	-2.61216397567728	1.66944169847988	-2.28022127603176
H	-2.72751922596184	2.88860693315609	-1.01299548803607
H	-5.10122161803232	0.19522741425126	0.04817269526659
H	-4.91753634710318	1.91424662793591	-0.28208362803840
H	-4.77560265942308	0.74606302497822	-1.59106688835752
H	1.03814170657422	0.14832906177685	0.87652452900487

Table S4. Equilibrium geometry of camphorquinone oxime $\mathscr{O}$ isomer.

Atom	x /Å	y /Å	z /Å
C	-3.22765450569235	-0.13537880021941	1.86355718677228
C	-3.07163103388937	-1.26143414418651	0.80440415745699
C	-2.43290506367034	-0.55346562530553	-0.43028842841079
C	-0.99448124508197	-0.27456596253290	0.01818493814008
C	-1.14249475885417	0.80743940720356	1.06375363488749
C	-2.61733032948456	1.09675612856434	1.14821667567221
C	-2.53895988225188	-1.31128612662381	-1.73158956142734
C	-3.04800788159037	0.88457977664465	-0.33491877923507
C	-2.39770448359497	1.88580280462938	-1.29287840304476
C	-4.56080887318064	0.93179812872147	-0.54157269493781
O	0.01275273113563	-0.80920317472797	-0.36405952770069
N	-0.14521813614487	1.27120050702913	1.69571509780066
O	-0.52180943023364	2.23953992847623	2.62983011527500
H	-4.27255729839566	0.04398203386188	2.11387423756205
H	-2.70423458452217	-0.35657688752898	2.79255592246734
H	-2.44782597647372	-2.08451403692475	1.15367965098115
H	-4.03342547952477	-1.68807612329545	0.51961522888946
H	-2.86569493795892	2.05823710551998	1.58880918267961
H	-2.08672877758735	-0.75411622645073	-2.55321070637947
H	-2.01384375387815	-2.26476224782136	-1.66034810161247
H	-3.58190548396204	-1.51330830196936	-1.98210471058118
H	-1.30993140698600	1.90599708113758	-1.21265751749876
H	-2.64850194934930	1.64607715333614	-2.32764642777822
H	-2.76204890648270	2.89485832776371	-1.08915718067750
H	-5.10585418110598	0.21651765540020	0.07074703909587
H	-4.93716970049244	1.92898432826971	-0.30164424413862
H	-4.80825760398642	0.73052529754085	-1.58549333916030
H	0.32345293323915	2.50346199348794	3.01293655490277

Table S5. Transition state geometry of camphorquinone oxime between the $\mathscr{O}$ and $\mathscr{C}$ isomers.

Atom	x /Å	y /Å	z /Å
C	-0.70707839552806	-0.45249000723853	1.80837872984029
C	-0.65596986039809	-1.55153439163266	0.71098808876120
C	-0.03072547080905	-0.84270962692468	-0.53011888442351
C	1.43512968899321	-0.67067325459938	-0.14125360242248
C	1.43039687758406	0.39504957828785	0.96394446458455
C	-0.05159857884369	0.76104758851661	1.11107727141248
C	-0.25299387380425	-1.54320637401876	-1.84958973571327
C	-0.54878782353273	0.63001991543080	-0.35639855659082
C	0.12564579533912	1.62283476906616	-1.30717768141574
C	-2.06231728830946	0.76940948242597	-0.51348586598058
O	2.39756569865636	-1.25344563192870	-0.57303763682450
N	2.42786369385056	0.78723688979387	1.58145194078497
O	3.46771611971721	1.23174303533198	2.24740618605469
H	-1.73168458009345	-0.23151344725778	2.10704344139694
H	-0.16224132976086	-0.73590154296791	2.70894753975973
H	-0.06537649850227	-2.41835401321430	1.00970214825539
H	-1.65046492338655	-1.91764300246576	0.45481546294500
H	-0.21854588467876	1.72216378906138	1.59409558030691
H	0.18827399633246	-0.98285643394933	-2.67535426177783
H	0.21738381636268	-2.52760353642182	-1.83949956862546
H	-1.31770450236610	-1.67450006041185	-2.05047091241045
H	1.21481194886001	1.57746806324190	-1.26725732439061
H	-0.17537553726841	1.43312096466847	-2.33923367829233
H	-0.17059105564231	2.64430243252837	-1.05867298696214
H	-2.62701720952155	0.07280263557763	0.10301323051912
H	-2.37389764271341	1.78074200154448	-0.24140574712096
H	-2.35543978403162	0.60491149169637	-1.55240347614220
H	4.25302260349499	0.76957868585962	1.89449583447161

2.2.2 B3LYP D3BJ OPT FREQ aug-cc-pVTZ Defgrid3 ExtremeSCF

Table S6. Equilibrium geometry of camphorquinone oxime $\mathscr{C}$ isomer.

Atom	x /Å	y /Å	z /Å
C	-3.26732965772666	-0.20860882192368	1.85472268442164
C	-3.08037348096045	-1.31776168861248	0.78292315763820
C	-2.42241044643897	-0.58727117154498	-0.43354176819297
C	-1.00816558878626	-0.31812283258086	0.05150124863114
C	-1.14650140099864	0.72778058582128	1.10109646656201
C	-2.61968398206172	1.02704955079482	1.18417970587921
C	-2.51782112352543	-1.31466563163182	-1.75359335653467
C	-3.03414182990737	0.85651362342431	-0.31215605300044
C	-2.36677002098296	1.87807639804542	-1.23732201378269
C	-4.54356686238622	0.91131973747883	-0.54011835376202
O	0.03542305355347	-0.83156385553408	-0.31523341889580
N	-0.22900645525237	1.28715686456836	1.79288063401911
O	1.05752999505191	0.87579257656968	1.55608469976687
H	-4.31994804468869	-0.02681756943124	2.06105938253782
H	-2.78476811667649	-0.44910035614507	2.79906632751919
H	-2.45566555524602	-2.13956071306391	1.12961801354540
H	-4.02960412457059	-1.74978074492768	0.47119211250819
H	-2.85435804427730	1.98236351708640	1.64449606631516
H	-1.91986795912788	-0.82380920033902	-2.52082695220278
H	-2.15047494267209	-2.33616787477319	-1.65830318051004
H	-3.55195869701952	-1.35805079009614	-2.09536621154567
H	-1.27878057870738	1.82150379014373	-1.22600641411221
H	-2.69301170763661	1.73221989626990	-2.26701021273144
H	-2.64257049011647	2.89111469754221	-0.94185376342875
H	-5.09106858351917	0.12808385510679	-0.02257516540166
H	-4.93477334113932	1.87114345202537	-0.19858866112379
H	-4.77026422180278	0.82538606827136	-1.60269912471409
H	1.04063220762220	0.18204663745568	0.85926415059508

Table S7. Equilibrium geometry of camphorquinone oxime $\mathscr{O}$ isomer.

Atom	x /Å	y /Å	z /Å
C	-3.22862424512296	-0.14806003139443	1.86565997873282
C	-3.07744039215896	-1.26793311070759	0.79932739434458
C	-2.43380677628875	-0.55460642202659	-0.42930870913754
C	-0.99721906774571	-0.28206893711468	0.02640334846739
C	-1.14477733368253	0.80331520146175	1.06919545983009
C	-2.61974638050740	1.08879340828437	1.15618189629894
C	-2.53762471845151	-1.30552618057617	-1.73478690868095
C	-3.04746627127990	0.88334697603294	-0.32890123450411
C	-2.39458365007553	1.88709121975672	-1.28274137409825
C	-4.55952609188710	0.93231208810649	-0.54031895428284
O	0.00957544894860	-0.82598802393478	-0.34633920490995
N	-0.14411921455314	1.28418114889439	1.68203576324758
O	-0.51884007815082	2.26369752900919	2.61042320048630
H	-4.27109364776886	0.03032209587760	2.12191706138536
H	-2.70084543170783	-0.37542448019398	2.78946260287593
H	-2.45933471175212	-2.09629668151883	1.14275557690116
H	-4.04041066609769	-1.68630445453908	0.50999519756245
H	-2.86904039024249	2.04666223520565	1.60137770790176
H	-2.06100051484194	-0.75719053895771	-2.54726422835608
H	-2.03626530950598	-2.27026889496766	-1.65884752417490
H	-3.58044196112568	-1.48224915515785	-2.00033865038694
H	-1.30794379988397	1.90837313476245	-1.20062027303905
H	-2.64306251632128	1.64932605686365	-2.31750512262556
H	-2.75975220887792	2.89458901747021	-1.07796877922294
H	-5.10882087432240	0.22897003631233	0.08036060849965
H	-4.93322132909298	1.93352401889932	-0.31840306166920
H	-4.80273848551519	0.71569283438927	-1.58118072678603
H	0.32739061801209	2.54078990976300	2.97773895534034

2.2.3 B3PW D3BJ OPT FREQ def2-TZVP RIJCOSX

Table S8. Equilibrium geometry of camphorquinone oxime $\mathscr{C}$ isomer.

Atom	x /Å	y /Å	z /Å
C	-3.25617553878852	-0.20474409161847	1.84826868413714
C	-3.08353158785412	-1.30328764867467	0.77314163585426
C	-2.42529187389142	-0.57588481842790	-0.43609561332736
C	-1.01524384291779	-0.31403212719433	0.05001744730356
C	-1.14972859849551	0.72646941660780	1.09570651164053
C	-2.61647703528311	1.02898844101080	1.18142526073186
C	-2.52079222289814	-1.29696769324877	-1.75327498936622
C	-3.03095257534209	0.86204717298599	-0.30765501323143
C	-2.36412369605169	1.87507051131140	-1.23099919285816
C	-4.53391714697003	0.91281621431359	-0.53577550830003
O	0.02434245092969	-0.84106675312723	-0.30523715355272
N	-0.22406786558941	1.26072894071016	1.79813231400097
O	1.03781885864232	0.82836266391150	1.56133681284626
H	-4.30724328766143	-0.02469742788124	2.07527573159965
H	-2.75773858243698	-0.45084734724486	2.78544906655590
H	-2.47139792803165	-2.13949536627509	1.11427574879170
H	-4.04043814496541	-1.71970899898812	0.45525097618654
H	-2.85149888075424	1.98562653395659	1.64390650125545
H	-2.04922501636641	-0.72487226949587	-2.55399407738611
H	-2.01231578894915	-2.26115113325523	-1.69947703729930
H	-3.56303500854029	-1.47692709709172	-2.02292759731797
H	-1.27702027975056	1.89006578182774	-1.13211620561724
H	-2.60012296174012	1.65740077052763	-2.27459905503178
H	-2.72608014716407	2.88204367758200	-1.01256678514983
H	-5.08708073454287	0.17980227733624	0.04907374547812
H	-4.91523131926961	1.90282538567917	-0.27413805146139
H	-4.76558438982806	0.74090591144878	-1.58895762616647
H	1.00285314451075	0.13679907331408	0.85944346968403

Table S9. Equilibrium geometry of camphorquinone oxime $\mathscr{O}$ isomer.

Atom	x /Å	y /Å	z /Å
C	-3.22793868867505	-0.13158730237867	1.85568320313985
C	-3.06850674877952	-1.25270387043379	0.80186616320031
C	-2.43508668434866	-0.54794343971289	-0.42838376027015
C	-1.00144551977619	-0.26967970955847	0.01792459259777
C	-1.15098372488503	0.80740045813461	1.06013849532540
C	-2.62067092686325	1.09562422086729	1.14464026368028
C	-2.54193487143552	-1.30145679956889	-1.72608839565976
C	-3.04729997289258	0.88305958162822	-0.33203175226445
C	-2.39471373081370	1.87791191407276	-1.28492440712855
C	-4.55390495827997	0.92479264753445	-0.53809855256781
O	0.00431091664849	-0.80529176424919	-0.36303791472077
N	-0.15376502588400	1.27195492111524	1.69177176450911
O	-0.52180033446889	2.23060536415498	2.61728874964038
H	-4.27471396729365	0.04716613062641	2.10320877559696
H	-2.70735705926684	-0.35140875000986	2.78753681839920
H	-2.44330306804400	-2.07570278472022	1.15142769539303
H	-4.02961830852672	-1.68287049974254	0.51571451161005
H	-2.86899073677295	2.05849777123635	1.58478606442391
H	-2.08846738718874	-0.74347625606499	-2.54699323773611
H	-2.01691806399226	-2.25558931923498	-1.65609145805847
H	-3.58540534805353	-1.50297699936969	-1.97669464684661
H	-1.30655912842036	1.89700053176187	-1.19526004707162
H	-2.63753085863584	1.63465095172036	-2.32128033550282
H	-2.75988319848855	2.88834139749461	-1.08746326292647
H	-5.09422048869400	0.20313739582100	0.07272436421104
H	-4.93623787966932	1.91920678382683	-0.29444428208801
H	-4.80056059438295	0.72549673595252	-1.58307315857455
H	0.32272635788365	2.49491068909669	2.99746374968880

2.2.4 PBE0 D3BJ OPT FREQ def2-TZVP RIJCOSX

Table S10. Equilibrium geometry of camphorquinone oxime $\mathscr{C}$ isomer.

Atom	x /Å	y /Å	z /Å
C	-3.25518399209599	-0.20378901691814	1.84592852099486
C	-3.08148713108600	-1.30093542728559	0.77204596844888
C	-2.42561718673114	-0.57455706357797	-0.43601747096012
C	-1.01703060996224	-0.31402701725832	0.04914499194226
C	-1.15089553179689	0.72549364530418	1.09505383085898
C	-2.61606815927184	1.02814472966058	1.18027164107002
C	-2.51926550319730	-1.29725300205304	-1.75132026592084
C	-3.02980692261959	0.86161984393024	-0.30686440332602
C	-2.36536967335789	1.87467167080531	-1.22963871600579
C	-4.53171283187994	0.91340196892560	-0.53433645211397
O	0.02054341021662	-0.84007994147462	-0.30667883953740
N	-0.22471988344812	1.25690639105627	1.79585629209305
O	1.03118703945883	0.82483558233927	1.56031736662218
H	-4.30707270218969	-0.02424240187278	2.07232096742493
H	-2.75758361456221	-0.44957756920480	2.78425815227831
H	-2.46861177071844	-2.13746279560779	1.11334162957458
H	-4.03853014034909	-1.71906213036381	0.45437289494308
H	-2.85041720656751	1.98570677685914	1.64315691278114
H	-2.04750648989436	-0.72644369492435	-2.55359182050945
H	-2.00871972577645	-2.26074723592135	-1.69510280295142
H	-3.56128466960543	-1.48024344698125	-2.02190943011951
H	-1.27758001147874	1.89296405202264	-1.13171283912515
H	-2.60179195726937	1.65696108061276	-2.27373567513893
H	-2.72983696618038	2.88131309946680	-1.01133213174972
H	-5.08652824352376	0.18087232243616	0.05069256655490
H	-4.91196616361927	1.90439170754360	-0.27272396494139
H	-4.76421547881148	0.74239899910496	-1.58807546653191
H	0.99777211631775	0.13500887337629	0.85916854334441

Table S11. Equilibrium geometry of camphorquinone oxime $\mathscr{O}$ isomer.

Atom	x /Å	y /Å	z /Å
C	-3.22768859395380	-0.12985501071553	1.85356482220415
C	-3.06688891693145	-1.24976531312012	0.80125967639901
C	-2.43534273877546	-0.54630050147655	-0.42792002844721
C	-1.00397676252618	-0.26828938937638	0.01753002204785
C	-1.15294719026793	0.80650261314539	1.05936001983818
C	-2.62094486534097	1.09568378693952	1.14364991489234
C	-2.54012219560719	-1.30166753141685	-1.72352338183194
C	-3.04666748690370	0.88313754951529	-0.33127325359674
C	-2.39635008977200	1.87769347401323	-1.28377925111568
C	-4.55213501125773	0.92530342773963	-0.53696103311092
O	0.00077453891605	-0.80199500080568	-0.36379831678853
N	-0.15667700140469	1.26906930464674	1.69136056437413
O	-0.52150200438588	2.22245334295899	2.61298569134758
H	-4.27537901582857	0.04821653913875	2.10024702382198
H	-2.70819117807590	-0.34917443046127	2.78681783804801
H	-2.44087831584090	-2.07288272031000	1.15121545015746
H	-4.02799086252236	-1.68176482833587	0.51523346340197
H	-2.86855457194899	2.05961082100521	1.58415240413954
H	-2.08653861127230	-0.74508402076535	-2.54602849158914
H	-2.01245540467380	-2.25467599049542	-1.65085836468360
H	-3.58318410854111	-1.50680536828059	-1.97509072741687
H	-1.30756031782337	1.90011667737789	-1.19468645258063
H	-2.63902081577413	1.63373475835152	-2.32060525301970
H	-2.76432936655586	2.88780998034311	-1.08720889034176
H	-5.09396576481454	0.20413716303366	0.07418313918661
H	-4.93384472053523	1.92063149777021	-0.29381385601845
H	-4.79938515394364	0.72639872644431	-1.58245667373981
H	0.32096652636168	2.48683044313613	2.99475394442211

2.2.5 REVPBE38 D3BJ OPT FREQ def2-TZVP RIJCOSX

Table S12. Equilibrium geometry of camphorquinone oxime $\mathscr{C}$ isomer.

Atom	x /Å	y /Å	z /Å
C	-3.25405705876968	-0.20458797687442	1.84233074468178
C	-3.08109759333725	-1.30048420198221	0.76972611397309
C	-2.42837964912730	-0.57445936792340	-0.43541958619701
C	-1.01875799123244	-0.31821475314182	0.04442635690783
C	-1.15191293526333	0.72448337279749	1.09386423289240
C	-2.61682232105057	1.02475471803263	1.17692779315301
C	-2.52662572456132	-1.29469365643720	-1.75020435405458
C	-3.02812730138325	0.85748749518521	-0.30631874356709
C	-2.36047494909491	1.86761559143447	-1.22691616894612
C	-4.52783432341472	0.91187596747147	-0.53400783947948
O	0.01015750835390	-0.84154398466192	-0.31576616005657
N	-0.23453297508555	1.26115153489339	1.78972763564993
O	1.02413759406029	0.84124711405971	1.56434606795832
H	-4.30239604846059	-0.02590649790457	2.06817149685431
H	-2.75560036134637	-0.44991586918417	2.77631841526169
H	-2.46760108993386	-2.13244692301127	1.10984592139805
H	-4.03490071251994	-1.71784292048116	0.45477352978256
H	-2.85074097635461	1.97937768255124	1.63687428944495
H	-2.05094486521234	-0.72739351885266	-2.54782403969904
H	-2.02634427626746	-2.25961265043118	-1.69289968917518
H	-3.56751035604405	-1.46625816000334	-2.01881030586976
H	-1.27597247325101	1.87777747461642	-1.12974444987785
H	-2.59931027095806	1.65274888535415	-2.26732838089825
H	-2.71777391534128	2.87235720513391	-1.00497542794566
H	-5.07946810635925	0.17693565390923	0.04405901918381
H	-4.90549312753051	1.89817766657259	-0.26512949557516
H	-4.75767445758466	0.74815260160627	-1.58581583255281
H	1.00675875707024	0.15548751727112	0.87265885675280

Table S13. Equilibrium geometry of camphorquinone oxime $\mathscr{O}$ isomer.

Atom	x /Å	y /Å	z /Å
C	-3.22706813932334	-0.12794373705333	1.85072457654033
C	-3.06420395188912	-1.24705820588323	0.80060631400731
C	-2.43567802139776	-0.54411211965625	-0.42625939472030
C	-1.00679838112297	-0.26648281072824	0.01656866816816
C	-1.15557543283677	0.80830634019306	1.05903091187846
C	-2.62303271629023	1.09556819988131	1.14118022539380
C	-2.54341787056735	-1.29811469081213	-1.72086082151172
C	-3.04546990966916	0.88076431098969	-0.33025914478723
C	-2.39406455444178	1.87343727706709	-1.28095257053219
C	-4.54873690028570	0.92200383680867	-0.53711814485810
O	-0.00481783049976	-0.79282278817309	-0.36615790136992
N	-0.16404292175899	1.26894289864118	1.68922281074428
O	-0.52350379110269	2.21909123885883	2.60789485690110
H	-4.27181683029896	0.04764885924654	2.09538246981717
H	-2.70729133938709	-0.34522613362324	2.78022135985529
H	-2.43658349267057	-2.06429566815410	1.15043264947922
H	-4.02108830848802	-1.67983856615235	0.51656368831587
H	-2.87154944229844	2.05638123492424	1.57782351782737
H	-2.08481392351901	-0.74549262841654	-2.53853551974423
H	-2.02634452497809	-2.25278605488897	-1.64545560771374
H	-3.58518442979965	-1.49197067341479	-1.97193016129978
H	-1.30868469403580	1.89089435185061	-1.19193737270664
H	-2.63814274640565	1.63097526659405	-2.31406009916216
H	-2.75782176096241	2.88087856005977	-1.08155447985056
H	-5.08622326711634	0.19699942852505	0.06664646116952
H	-4.93009154881745	1.91196886538982	-0.28680642087860
H	-4.79212787737947	0.73008257251932	-1.58113053860805
H	0.31339460734264	2.48127083540704	2.98902966764531

2.2.6 TPSSh D3BJ OPT FREQ def2-TZVP RIJCOSX

Table S14. Equilibrium geometry of camphorquinone oxime $\mathscr{C}$ isomer.

Atom	x /Å	y /Å	z /Å
C	-3.26136998328691	-0.20366748210395	1.85385069245518
C	-3.08724098174940	-1.30605057268646	0.77507926004139
C	-2.42199846554435	-0.57620860381321	-0.43770453412778
C	-1.01154681089886	-0.31047041098525	0.05253645245977
C	-1.14612268150721	0.72789770836677	1.09726252349717
C	-2.61600670275064	1.03399049771679	1.18595540552680
C	-2.51636783216344	-1.30308262125112	-1.75972757427338
C	-3.03238108575679	0.86607353074300	-0.30793888053995
C	-2.36474929006695	1.88464698005451	-1.23475710478341
C	-4.54187066178271	0.91229000080447	-0.53565536377364
O	0.03677137599610	-0.84039626202761	-0.30144213883596
N	-0.21580613716463	1.26220298978705	1.80703555515856
O	1.05319786300740	0.81844405160898	1.55768034982697
H	-4.31414538409267	-0.02158843609731	2.07469454074456
H	-2.76478745097551	-0.45166745597244	2.79201685374102
H	-2.47570856045847	-2.14247962579589	1.11785756313386
H	-4.04599608558366	-1.71767260117690	0.45396722119355
H	-2.85082581320215	1.99052607102572	1.64987373482896
H	-2.04918000586423	-0.72792629164973	-2.56184042392825
H	-2.00161110717751	-2.26448940711537	-1.70315754286435
H	-3.55934779634464	-1.48859416224164	-2.02591281209828
H	-1.27713985730794	1.89800826718935	-1.13077407317345
H	-2.59949813825407	1.66463579786545	-2.27903814154508
H	-2.73017914951372	2.89105500417136	-1.01563142660959
H	-5.08924207645297	0.17902178713819	0.05642764271956
H	-4.92450267720115	1.90380012100462	-0.27852473591670
H	-4.77239568003765	0.73244257211045	-1.58861052595976
H	1.00075117613483	0.12552855333016	0.84936748310222

Table S15. Equilibrium geometry of camphorquinone oxime $\mathscr{O}$ isomer.

Atom	x /Å	y /Å	z /Å
C	-3.26136998328691	-0.20366748210395	1.85385069245518
C	-3.08724098174940	-1.30605057268646	0.77507926004139
C	-2.42199846554435	-0.57620860381321	-0.43770453412778
C	-1.01154681089886	-0.31047041098525	0.05253645245977
C	-1.14612268150721	0.72789770836677	1.09726252349717
C	-2.61600670275064	1.03399049771679	1.18595540552680
C	-2.51636783216344	-1.30308262125112	-1.75972757427338
C	-3.03238108575679	0.86607353074300	-0.30793888053995
C	-2.36474929006695	1.88464698005451	-1.23475710478341
C	-4.54187066178271	0.91229000080447	-0.53565536377364
O	0.03677137599610	-0.84039626202761	-0.30144213883596
N	-0.21580613716463	1.26220298978705	1.80703555515856
O	1.05319786300740	0.81844405160898	1.55768034982697
H	-4.31414538409267	-0.02158843609731	2.07469454074456
H	-2.76478745097551	-0.45166745597244	2.79201685374102
H	-2.47570856045847	-2.14247962579589	1.11785756313386
H	-4.04599608558366	-1.71767260117690	0.45396722119355
H	-2.85082581320215	1.99052607102572	1.64987373482896
H	-2.04918000586423	-0.72792629164973	-2.56184042392825
H	-2.00161110717751	-2.26448940711537	-1.70315754286435
H	-3.55934779634464	-1.48859416224164	-2.02591281209828
H	-1.27713985730794	1.89800826718935	-1.13077407317345
H	-2.59949813825407	1.66463579786545	-2.27903814154508
H	-2.73017914951372	2.89105500417136	-1.01563142660959
H	-5.08924207645297	0.17902178713819	0.05642764271956
H	-4.92450267720115	1.90380012100462	-0.27852473591670
H	-4.77239568003765	0.73244257211045	-1.58861052595976
H	1.00075117613483	0.12552855333016	0.84936748310222

2.2.7 DLPNO-MP2 def2-TZVP def2-TZVP/C OPT NUMFREQ

Table S16. Equilibrium geometry of camphorquinone oxime $\mathscr{C}$ isomer.

Atom	x /Å	y /Å	z /Å
C	-3.25711126868339	-0.20530715047640	1.85123742256229
C	-3.08166124346278	-1.30573208680158	0.77278161269538
C	-2.42964339030789	-0.57526922034385	-0.43697608491130
C	-1.02150395952881	-0.31863019783656	0.04386527385466
C	-1.15477256975267	0.72283836400591	1.09415314292850
C	-2.61884558167215	1.02952162541696	1.18207463515702
C	-2.52614834032974	-1.29801827684249	-1.75556325014820
C	-3.02928442476345	0.85904293710370	-0.30581356785163
C	-2.35901632340399	1.87367480920227	-1.22740973728291
C	-4.53311786479970	0.91196300287653	-0.53750150773811
O	0.02195691871137	-0.85071981512026	-0.32405125239723
N	-0.22921508492190	1.27496465402692	1.80771027264589
O	1.04586107822866	0.83884511958614	1.56367906487308
H	-4.30954285395635	-0.02811326234375	2.07795770546450
H	-2.75437895818783	-0.44942039693316	2.78823646953737
H	-2.46095501853451	-2.13893012314104	1.11180387257642
H	-4.03893050179851	-1.72703009613193	0.45751217351702
H	-2.85074922323813	1.99074893954188	1.64279776705769
H	-2.04208365809039	-0.73258659255871	-2.55459400073636
H	-2.02807982176511	-2.26806907693754	-1.69381123835275
H	-3.56980737707006	-1.46667001574319	-2.02920736577571
H	-1.27079275705340	1.88943440715176	-1.12834795173005
H	-2.59705029013658	1.65836907992142	-2.27203078761380
H	-2.72270395875454	2.88032248400464	-1.00465945371689
H	-5.08915884068751	0.17867401982625	0.04577626220858
H	-4.91188513887429	1.90408253475560	-0.27620796095461
H	-4.75972114740750	0.74136711268793	-1.59288690112364
H	0.99904160024126	0.14691722110253	0.86236538525474

Table S17. Equilibrium geometry of camphorquinone oxime $\mathscr{O}$ isomer.

Atom	x /Å	y /Å	z /Å
C	-3.22405978389213	-0.12261231968750	1.85929626665954
C	-3.05902311249461	-1.24925252515740	0.80621171354135
C	-2.43658281156771	-0.54537299078478	-0.42999906991825
C	-1.00560222555796	-0.26485545973787	0.00421499786830
C	-1.15448114022099	0.80543110359732	1.05259858374091
C	-2.61933361819668	1.10386425175295	1.14127854872428
C	-2.54978373817932	-1.30431192364762	-1.72636495690072
C	-3.04520978958297	0.88231253449569	-0.33278899923423
C	-2.39315753167405	1.87711880396277	-1.28819521889389
C	-4.55299950311468	0.92202082400462	-0.53991901451117
O	0.01058640768863	-0.79306783364592	-0.39526027514347
N	-0.14514474196691	1.25919017498989	1.69900841952005
O	-0.54450911320630	2.21839357854038	2.63256521512859
H	-4.27280869645203	0.05224179986805	2.10491643956426
H	-2.69916157892317	-0.33584296389634	2.79193959097797
H	-2.42168936104378	-2.06506236732109	1.15654112304282
H	-4.01889217779416	-1.68971569098761	0.52631987012859
H	-2.86380496370200	2.07250449961624	1.57739429113901
H	-2.08607659910553	-0.75535348686700	-2.54860623322957
H	-2.03556176930130	-2.26448992174970	-1.64717654320497
H	-3.59579389856289	-1.49464313225705	-1.97718268228896
H	-1.30372124660254	1.89851683219507	-1.20062332412633
H	-2.64006771390018	1.63294304819555	-2.32437671460748
H	-2.76118079820025	2.88720837238135	-1.08817492573507
H	-5.09360675317791	0.20075878993785	0.07254030133904
H	-4.93459074691955	1.91848395613411	-0.29926370172255
H	-4.79612101134850	0.72036268758537	-1.58615718010274
H	0.30159801699950	2.47229935848263	3.02757347824466

2.2.8 M062X OPT FREQ def2-TZVP RIJCOSX

Table S18. Equilibrium geometry of camphorquinone oxime $\mathscr{C}$ isomer.

Atom	x /Å	y /Å	z /Å
C	-3.25782110326059	-0.20769948382591	1.85186150272782
C	-3.08915215754611	-1.30878670621161	0.77177565160883
C	-2.43035248409727	-0.57874530964935	-0.43618936220484
C	-1.01480556350231	-0.32736033123318	0.04408087105105
C	-1.14368859972451	0.72487319835935	1.10336035187119
C	-2.61561772945244	1.02720993172910	1.18354576413082
C	-2.52324146213636	-1.29862349277189	-1.75787748384497
C	-3.02908663388809	0.85973651723789	-0.30655248993154
C	-2.36431870139772	1.87313667158766	-1.23640480235223
C	-4.53583556917992	0.91465291972241	-0.53448411398927
O	0.00960941931197	-0.85542467907926	-0.32476954031071
N	-0.22905379748959	1.27083516586080	1.79631182985768
O	1.04474256971049	0.85423641979100	1.57742443225675
H	-4.30692254264768	-0.02550192544509	2.07990454978596
H	-2.75595599375437	-0.45579761820165	2.78531416811480
H	-2.47665683417701	-2.14506208854254	1.11034754342560
H	-4.04680837639887	-1.72147946871986	0.45429421844021
H	-2.84732748456287	1.98527134692138	1.64370171686487
H	-2.03331916045158	-0.73352255047241	-2.55202545592883
H	-2.02950671877549	-2.26932546955544	-1.69451927580933
H	-3.56581302300369	-1.46284777620935	-2.03496734219186
H	-1.27600886396715	1.88167677155225	-1.15464621256499
H	-2.61880202757694	1.65902520444124	-2.27597007719503
H	-2.71899066884008	2.87993666386050	-1.00827469300867
H	-5.09121498130389	0.17627731599617	0.04050937656130
H	-4.91298837659527	1.90265303541190	-0.26166368529600
H	-4.76411577304373	0.75549547182430	-1.59006882132084
H	1.04375263775118	0.16143026562155	0.88887137925222

Table S19. Equilibrium geometry of camphorquinone oxime $\mathscr{O}$ isomer.

Atom	x /Å	y /Å	z /Å
C	-3.22535042496735	-0.12349045008420	1.86036098374590
C	-3.06431264967727	-1.25093785458675	0.80607215188318
C	-2.43489303570900	-0.54680443296301	-0.42861075979313
C	-1.00128507700214	-0.26797560135269	0.01072524670810
C	-1.14572259743555	0.81041206408498	1.06271391995397
C	-2.61854272824745	1.10505119444139	1.14390332936865
C	-2.54004785154652	-1.30439869329339	-1.72779076776982
C	-3.04566442695895	0.88457339584147	-0.33358211009745
C	-2.40062486177796	1.87889467608410	-1.29708791620964
C	-4.55626815731173	0.92294323920809	-0.53835439898769
O	0.00271450979844	-0.79219363543651	-0.37659336196977
N	-0.15257962910017	1.25866567437503	1.70142679434994
O	-0.52455832755275	2.21021890361740	2.63032973388619
H	-4.27136525650692	0.05380148221777	2.10617025705180
H	-2.70142673280040	-0.33921553471232	2.79000080023676
H	-2.43378466357036	-2.06869044293782	1.15648304317730
H	-4.02426296488478	-1.68361140499396	0.52352476793940
H	-2.86439076177003	2.07060590859543	1.57869229308951
H	-2.06972950245011	-0.75504635474001	-2.54446590689311
H	-2.02764748874330	-2.26382016238124	-1.64485790392957
H	-3.58388573129512	-1.49360062559355	-1.98469160779924
H	-1.31152640004261	1.89727524798104	-1.22410783740730
H	-2.66072450431702	1.63401878308445	-2.32843252998828
H	-2.76410085105296	2.88822991102901	-1.09412508781550
H	-5.09555805571862	0.19523658405109	0.06519046140318
H	-4.93826268204182	1.91480713461164	-0.28641988133149
H	-4.79909236264637	0.73202570397609	-1.58545872541149
H	0.31211321532888	2.47209528987645	3.02729501260958

Table S20. Transition state geometry of camphorquinone oxime between $\mathscr{O}$ and $\mathscr{C}$ isomers.

Atom	x /Å	y /Å	z /Å
C	-0.70594981005322	-0.44679457722006	1.80554678632483
C	-0.65138221957947	-1.54447065934390	0.70958100717591
C	-0.03235479886620	-0.83607969490631	-0.52746709517094
C	1.42760556472360	-0.66527637735420	-0.14110048426605
C	1.42615236452329	0.39993373790732	0.96890975474804
C	-0.05438116891530	0.76616950286015	1.10993498576773
C	-0.24846163712603	-1.53192110935354	-1.84747648809818
C	-0.54680659952724	0.62802949242007	-0.35348984325778
C	0.12203386466870	1.61700505964053	-1.30684887242993
C	-2.05807464778092	0.75881054140494	-0.50939339443362
O	2.38725552973540	-1.23954581899338	-0.57429449596773
N	2.42209174890811	0.78220696839946	1.58324363498335
O	3.46636026263441	1.21165375913084	2.24233853271404
H	-1.73110783716416	-0.22749849760323	2.10164672043033
H	-0.15945361141843	-0.72842155932116	2.70498602098510
H	-0.05642462720040	-2.40884637057101	1.00725060502987
H	-1.64493864134444	-1.91289314476754	0.45277167987009
H	-0.22414556518829	1.72970421942963	1.58716586882161
H	0.21462395130025	-0.97731835950407	-2.66519643757313
H	0.20906013205762	-2.52208632732480	-1.82747008795141
H	-1.31278754908598	-1.64800214234134	-2.05920617767262
H	1.21208647758890	1.56613319950463	-1.27784788947920
H	-0.19228328722409	1.42848450399574	-2.33525540756103
H	-0.16891334338410	2.63842082222169	-1.05328096908759
H	-2.61866184412956	0.05860982680566	0.10753437330225
H	-2.37327410754101	1.76885501545012	-0.23806164304741
H	-2.34732335111736	0.59180549460861	-1.54916623138010
H	4.23945475050591	0.74333249482515	1.88464554722358

2.2.9 PBEh-3c OPT FREQ def2-TZVP RIJCOSX

Table S21. Equilibrium geometry of camphorquinone oxime $\mathscr{C}$ isomer.

Atom	x /Å	y /Å	z /Å
C	-3.24518827483912	-0.21181371419103	1.84257155590192
C	-3.08231744816891	-1.30596173815891	0.76564960662222
C	-2.42663464291956	-0.57981810859036	-0.43798216240754
C	-1.01350217143284	-0.33174661952944	0.03950412570152
C	-1.14291403548348	0.71633829654915	1.09050275819047
C	-2.60669828927346	1.01652405832892	1.17594450525571
C	-2.54001727873855	-1.29765280910246	-1.75442862143282
C	-3.02166342353716	0.85396773224355	-0.30634370337572
C	-2.36227969405345	1.86934250806292	-1.23176014616059
C	-4.52362702621223	0.91540820549334	-0.53565769443445
O	0.00408623975622	-0.86364770394581	-0.32009564600217
N	-0.24244430426725	1.26358758244989	1.79095510117149
O	1.02578752051395	0.88467896053855	1.60776357681213
H	-4.29173965960914	-0.03205321226085	2.07876870361452
H	-2.75016837461082	-0.46293531267766	2.77747632219963
H	-2.48359381966309	-2.15066767431557	1.10411186434797
H	-4.04098451403149	-1.71956156474997	0.45567529815892
H	-2.84007438622905	1.97105575041045	1.64148005856408
H	-2.07494654724393	-0.73830399789072	-2.56383208069162
H	-2.05221328854733	-2.26923845087222	-1.71359630732568
H	-3.58386189587374	-1.46398749207027	-2.01455758983564
H	-1.27671792178225	1.90036581404822	-1.15484266383391
H	-2.60619728818494	1.66006315914623	-2.27208394948405
H	-2.72281059369720	2.87318705684216	-1.01201343626347
H	-5.09297051804378	0.19945223929165	0.04965462575559
H	-4.90051514300563	1.90719659229788	-0.28736182636924
H	-4.76077951711636	0.73886815970054	-1.58377333334041
H	1.07568629629469	0.19362228295183	0.93116105866114

Table S22. Equilibrium geometry of camphorquinone oxime $\mathscr{O}$ isomer.

Atom	x /Å	y /Å	z /Å
C	-3.22997056899763	-0.13164185735343	1.85371719627498
C	-3.06285245255563	-1.24900563597732	0.80135877570041
C	-2.42758482578428	-0.54395207986190	-0.42140720891283
C	-1.00256826578084	-0.26208549920258	0.02929813690528
C	-1.15744914048564	0.81271172846731	1.06910216219553
C	-2.62603498461448	1.09513930821134	1.14812607306110
C	-2.54042342606326	-1.29600389193604	-1.71801378738567
C	-3.04144045908966	0.88078773945793	-0.32626768568549
C	-2.39902729907140	1.87962346628773	-1.28087373073706
C	-4.54584969643580	0.91937304669119	-0.54468872118476
O	0.00229692739872	-0.78128815381243	-0.34226080479635
N	-0.16311738278668	1.26727758106375	1.69103408193908
O	-0.48685739018886	2.21246637065830	2.60820811464040
H	-4.27563157600925	0.03951303103379	2.10054909405433
H	-2.72086768983620	-0.35330220785805	2.78876832232998
H	-2.44756170901485	-2.07504260638605	1.15552499647588
H	-4.01956153395760	-1.68401004133201	0.51500258823607
H	-2.89233302638863	2.05421176363730	1.58450814697074
H	-2.09208405553356	-0.74888119540566	-2.54498506172170
H	-2.03111729427676	-2.25551788889441	-1.65743719362726
H	-3.58274163338480	-1.49080125767891	-1.96591894497794
H	-1.31431823602487	1.93168401408002	-1.19990456591248
H	-2.63283350030302	1.63041864452136	-2.31468733708322
H	-2.78112859990483	2.88302989334522	-1.09719249762541
H	-5.10136134287180	0.21173421074523	0.06381917210581
H	-4.93388756233746	1.91264478460960	-0.31998661320984
H	-4.78791555168392	0.71061865421581	-1.58572060802213
H	0.35344227598304	2.46936807867286	2.97863789999253

Table S23. Transition state geometry of camphorquinone oxime between $\mathscr{O}$ and $\mathscr{C}$ isomers.

Atom	x /Å	y /Å	z /Å
C	-0.69677993190497	-0.44632866875438	1.79764088142039
C	-0.64028766482175	-1.53898704173553	0.70869131692130
C	-0.02311902453181	-0.83532694269320	-0.52501706154759
C	1.43188707879018	-0.65903622647207	-0.13973679861173
C	1.42308301543931	0.39665723908163	0.96118226047734
C	-0.04780928830492	0.75954529356482	1.10434077461773
C	-0.25502643697285	-1.53372579903962	-1.83705076083857
C	-0.53773247118491	0.62291655800261	-0.35322263070200
C	0.12616796778544	1.61451835697092	-1.30168398714688
C	-2.04428548608510	0.75905202553549	-0.51059783600546
O	2.39094328210013	-1.22706430608560	-0.56500481187607
N	2.41223637524428	0.77801211695691	1.57746998454179
O	3.43831367466226	1.20258531223860	2.23483354760761
H	-1.72065961207026	-0.22982593162962	2.09680393143790
H	-0.16131020987571	-0.72733711834703	2.70244424017107
H	-0.05663239293887	-2.40789339193510	1.01098957165039
H	-1.63173555612785	-1.91304667513263	0.45623234541857
H	-0.22315532183300	1.72071881923915	1.58310885663797
H	0.18204991712221	-0.98627589184019	-2.67014151044794
H	0.19581829377806	-2.52395148441953	-1.83509571478746
H	-1.31913116905769	-1.65480206724609	-2.03370659661888
H	1.21433818157689	1.59327288945640	-1.26974653185625
H	-0.16914347835391	1.42459054786075	-2.33264502496501
H	-0.17987454877275	2.63214696720684	-1.06209029099514
H	-2.61658406659415	0.06201277606395	0.09510504930456
H	-2.36329457759534	1.76374970815542	-0.23364447273399
H	-2.34212537122157	0.60586905208356	-1.54727647168386
H	4.21384882174866	0.74795388291352	1.88781774061019

2.2.10 DLPNO-SCS-MP2 def2-TZVP def2-TZVP/C OPT NUMFREQ

Table S24. Equilibrium geometry of camphorquinone oxime $\mathscr{C}$ isomer.

Atom	x /Å	y /Å	z /Å
C	-3.25662327898804	-0.21007826027747	1.85548041570889
C	-3.08531477580240	-1.31383209249984	0.77319745391801
C	-2.43119986005277	-0.58088062490603	-0.43901596477014
C	-1.01437421936437	-0.32548406515266	0.03899491280880
C	-1.14610078214375	0.72330135514061	1.09714466131666
C	-2.61729975460371	1.02739484559665	1.18318402954040
C	-2.52929242865791	-1.30857204017720	-1.76141103210895
C	-3.03056457536857	0.85871519710493	-0.30855881813741
C	-2.35955098222553	1.87907874054284	-1.23328716353607
C	-4.54006131311206	0.92014839217142	-0.54041608783987
O	0.02233823128093	-0.85778822608645	-0.33324149012179
N	-0.23194411423078	1.28205678464942	1.81368528584494
O	1.05834719574758	0.85680984426059	1.58254884033333
H	-4.30885534676721	-0.03223850343904	2.08597934013819
H	-2.75136528895594	-0.45667957863118	2.79155284320547
H	-2.46405579441361	-2.14752723459299	1.11280126110322
H	-4.04473815887017	-1.73497123323167	0.46268840630314
H	-2.84877409415890	1.98775093974939	1.64666974331003
H	-2.04132047934465	-0.74694117652865	-2.56198818495593
H	-2.03682412407842	-2.28229398718752	-1.69659696760673
H	-3.57444610608692	-1.47196927474914	-2.03652263674776
H	-1.26978068046777	1.89110887991162	-1.14318170131222
H	-2.60597378529350	1.67009159267246	-2.27814138941997
H	-2.71809824702993	2.88671401123326	-1.00218546120147
H	-5.10090117182908	0.18676103939674	0.03951709873736
H	-4.91404978491732	1.91380401563839	-0.27400131507933
H	-4.76820019909404	0.75548325951849	-1.59736032036175
H	1.02972391882894	0.16630739987302	0.88535424093089

Table S25. Equilibrium geometry of camphorquinone oxime $\mathscr{O}$ isomer.

Atom	x /Å	y /Å	z /Å
C	-3.22527590144414	-0.12710526364193	1.86437891451902
C	-3.06155924521752	-1.25684819595718	0.80767237023630
C	-2.43507688355827	-0.55004018347081	-0.43058934203139
C	-0.99918178019388	-0.26806062689077	0.00571476312833
C	-1.14691477533436	0.80706612844936	1.05771827646873
C	-2.61904245789360	1.10277432110363	1.14493417998710
C	-2.54739689607658	-1.31412956179926	-1.73097931071176
C	-3.04594280889077	0.88271385298234	-0.33409210218787
C	-2.39426946832627	1.88467286815207	-1.29209838678771
C	-4.55938095789955	0.92839477046352	-0.54378901107497
O	0.01648167264332	-0.79375467719167	-0.39180863279839
N	-0.13823516585125	1.26261554635195	1.70071931671588
O	-0.53855827878064	2.22848693632070	2.63666611729636
H	-4.27443802003604	0.04716533351673	2.11179638192491
H	-2.69906876482914	-0.34264749876877	2.79686806464136
H	-2.42389399208646	-2.07295947935654	1.15952023085207
H	-4.02279107929224	-1.69721588745223	0.53016357988496
H	-2.86509134825930	2.07036024433605	1.58292116753290
H	-2.07963222952069	-0.76816296887593	-2.55416861463630
H	-2.03790479359989	-2.27758932701582	-1.64806767112327
H	-3.59458687961408	-1.50038114155504	-1.98451019889387
H	-1.30363796460789	1.90584237456462	-1.21115711911253
H	-2.64701742192901	1.64537229500458	-2.32896623863819
H	-2.76087677034687	2.89495280167928	-1.08507462814785
H	-5.10472518374549	0.20584588606516	0.06427728414692
H	-4.93795699880562	1.92595479733028	-0.29832049752456
H	-4.80211428192475	0.73282323025986	-1.59227023353583
H	0.30730867542103	2.48292342539578	3.03085133986960

Table S26. Transition state geometry of camphorquinone oxime between $\mathscr{O}$ and $\mathscr{C}$ isomers.

Atom	*x* /Å	*y* /Å	*z* /Å
C	-0.70813768577397	-0.45353292060720	1.80885481731493
C	-0.65352539828973	-1.55321860882058	0.71014736372525
C	-0.03488378237297	-0.84238205893043	-0.52907582798074
C	1.42788724384157	-0.66835446623387	-0.14567580644551
C	1.42468871233179	0.39836114926898	0.96719493587748
C	-0.05775793377261	0.76173659309714	1.11107625317313
C	-0.25769219856419	-1.54571863762128	-1.85003551887411
C	-0.54744078534140	0.62439449252709	-0.35458007229777
C	0.13360796218363	1.61754111761061	-1.30193135848284
C	-2.06112433327264	0.76850505415180	-0.51447592881507
O	2.39881472557752	-1.24198126266250	-0.59001320935893
N	2.42450810322102	0.79682406525488	1.58533745727153
O	3.47510123389946	1.24721739452173	2.24765154873391
H	-1.73621549535127	-0.23607111791437	2.10714051409769
H	-0.15965124717586	-0.73506335018425	2.71133396462912
H	-0.05394221987649	-2.41843624464961	1.00802234762962
H	-1.64973823469977	-1.92606009552605	0.45749967067192
H	-0.22768276608570	1.72754564551938	1.59172707954976
H	0.20974795444126	-1.00062492965814	-2.67419150047904
H	0.18784348414705	-2.54372981357511	-1.82580086963311
H	-1.32495531954016	-1.65218221615125	-2.06283876510795
H	1.22542129815610	1.56423895948386	-1.26391729259385
H	-0.17245973185569	1.43628641866437	-2.33636361837354
H	-0.15610027980498	2.64118063280726	-1.04429965546447
H	-2.62953208109552	0.05980232949825	0.08917204344204
H	-2.37011205873798	1.77776199596915	-0.22332908746069
H	-2.35046900831006	0.62412812031801	-1.55963740983293
H	4.24379984212164	0.77183175384213	1.88100792508416

2.3 Transition frequencies

Table S27. Transition frequencies for the open $\mathscr{O}$ switch form. J is the rotational angular momentum quantum number, K_a and K_c are the projections of J onto the principal axes at the prolate and oblate symmetric top limits, and F is the total angular momentum quantum number, which includes the nuclear spin, I(^{14}N) = 1.

Observed Frequency /MHz	Calculated Frequency /MHz	Difference / MHz	J	K_a	K_c	F	J'	K'_a	K'_c	F'
2022.1284	2022.1108	0.0176	4	3	1	5	4	2	2	5
2125.8359	2125.8251	0.0108	5	2	4	6	5	1	5	6
2126.2715	2126.2764	-0.0049	5	2	4	5	5	1	5	5
2244.7276	2244.7377	-0.0101	4	3	2	5	4	2	3	5
2244.9567	2244.9527	0.0040	4	3	2	4	4	2	3	4
2326.7578	2326.7483	0.0095	5	3	3	6	5	2	4	6
2326.9605	2326.9599	0.0006	5	3	3	5	5	2	4	5
2642.4028	2642.3819	0.0209	7	3	5	8	7	2	6	8
2642.6247	2642.6241	0.0006	7	3	5	7	7	2	6	7
2746.2541	2746.2502	0.0039	2	1	2	3	1	1	1	2
2768.6681	2768.6846	-0.0165	7	4	3	8	7	3	4	8
2818.0915	2818.1192	-0.0277	7	2	6	8	7	1	7	8
2818.5163	2818.5314	-0.0151	7	2	6	7	7	1	7	7
2830.5253	2830.5272	-0.0019	2	0	2	3	1	0	1	2
2830.8551	2830.8432	0.0119	2	0	2	1	1	0	1	1
2885.1161	2885.1108	0.0053	8	3	6	8	8	2	7	8
2904.8525	2904.8558	-0.0033	6	4	2	7	6	3	3	7
2949.4837	2949.4877	-0.0040	2	1	1	1	1	1	0	1
2949.8588	2949.8604	-0.0016	2	1	1	2	1	1	0	1
2950.0166	2950.0174	-0.0008	2	1	1	3	1	1	0	2
2950.4653	2950.4792	-0.0139	2	1	1	1	1	1	0	0
3036.3820	3036.3642	0.0178	5	4	2	6	5	3	3	6
3036.4866	3036.4852	0.0014	5	4	2	5	5	3	3	5
3041.6262	3041.6077	0.0185	6	4	3	7	6	3	4	7
3041.7185	3041.7077	0.0108	6	4	3	6	6	3	4	6
3067.0650	3067.0726	-0.0076	7	4	4	8	7	3	5	8
3067.1663	3067.1719	-0.0056	7	4	4	7	7	3	5	7
3125.3665	3125.3775	-0.0110	8	4	5	9	8	3	6	9
3125.4914	3125.4887	0.0027	8	4	5	8	8	3	6	8
3133.0126	3133.0146	-0.0020	2	1	2	2	1	0	1	2
3133.1968	3133.1876	0.0092	2	1	2	2	1	0	1	1
3133.4364	3133.4396	-0.0032	2	1	2	3	1	0	1	2
3133.8523	3133.8486	0.0037	2	1	2	1	1	0	1	1
3216.4140	3216.4412	-0.0272	8	2	7	9	8	1	8	9
3216.8070	3216.8301	-0.0231	8	2	7	8	8	1	8	8
3228.3832	3228.3975	-0.0143	9	4	6	10	9	3	7	10
3228.5353	3228.5278	0.0075	9	4	6	9	9	3	7	9
3251.3285	3251.3342	-0.0057	3	1	2	4	2	2	1	3
3385.1028	3385.0941	0.0087	10	4	7	11	10	3	8	11
3546.8664	3546.8817	-0.0153	9	1	8	10	9	0	9	10
3675.4654	3675.4870	-0.0216	9	5	4	10	9	4	5	10
3717.4840	3717.4799	0.0041	4	2	2	4	3	3	1	3
3746.5849	3746.5967	-0.0118	4	1	4	4	3	2	1	3
3747.3276	3747.3398	-0.0122	4	1	4	5	3	2	1	4
3785.2746	3785.2819	-0.0073	8	5	3	9	8	4	4	9
3847.7256	3847.7362	-0.0106	9	5	5	10	9	4	6	10
3850.1212	3850.1162	0.0050	7	5	2	8	7	4	3	8

3853.9033	3853.9143	-0.0110	10	5	6	11	10	4	7	11
3858.6889	3858.6889	0.0000	8	5	4	9	8	4	5	9
3876.3641	3876.3587	0.0054	7	5	3	8	7	4	4	8
3901.7188	3901.7201	-0.0013	3	0	3	3	2	1	2	3
3901.9682	3901.9786	-0.0104	3	0	3	2	2	1	2	1
3902.1038	3902.0865	0.0173	3	0	3	4	2	1	2	3
3902.6393	3902.6397	-0.0004	3	0	3	2	2	1	2	2
4108.6070	4108.6195	-0.0125	3	1	3	3	2	1	2	3
4109.1082	4109.1292	-0.0210	3	1	3	4	2	1	2	3
4111.0798	4111.0726	0.0072	2	2	1	1	1	1	0	1
4111.3685	4111.3608	0.0077	2	2	1	2	1	1	0	1
4111.5763	4111.5721	0.0042	2	2	1	3	1	1	0	2
4111.7561	4111.7574	-0.0013	2	2	1	2	1	1	0	2
4112.0644	4112.0641	0.0003	2	2	1	1	1	1	0	0
4204.6314	4204.6325	-0.0011	3	0	3	3	2	0	2	3
4204.8788	4204.8899	-0.0111	3	0	3	3	2	0	2	2
4204.9977	4204.9989	-0.0012	3	0	3	4	2	0	2	3
4230.4744	4230.4800	-0.0056	2	2	0	1	1	1	1	0
4230.9699	4230.9584	0.0115	2	2	0	3	1	1	1	2
4231.2256	4231.2159	0.0097	2	2	0	2	1	1	1	2
4231.4416	4231.4396	0.0020	2	2	0	2	1	1	1	1
4271.9972	4272.0025	-0.0053	3	2	2	3	2	2	1	2
4272.1832	4272.1878	-0.0046	3	2	2	4	2	2	1	3
4272.3011	4272.2907	0.0104	3	2	2	2	2	2	1	1
4339.2629	4339.2592	0.0037	3	2	1	3	2	2	0	2
4339.3502	4339.3424	0.0078	3	2	1	4	2	2	0	3
4411.5333	4411.5319	0.0014	3	1	3	3	2	0	2	3
4411.7884	4411.7893	-0.0009	3	1	3	3	2	0	2	2
4412.0247	4412.0416	-0.0169	3	1	3	4	2	0	2	3
4412.4712	4412.4774	-0.0062	3	1	3	2	2	0	2	2
4412.8806	4412.8889	-0.0083	3	1	2	4	2	1	1	3
4822.8879	4822.9012	-0.0133	5	2	4	5	4	3	1	4
4823.2137	4823.2112	0.0025	5	2	4	6	4	3	1	5
4837.6483	4837.6498	-0.0015	4	1	3	5	3	2	2	4
4837.7640	4837.7602	0.0038	4	1	3	4	3	2	2	3
5021.3781	5021.3708	0.0073	3	1	2	4	2	0	2	3
5021.8015	5021.8016	-0.0001	3	1	2	3	2	0	2	2
5328.5044	5328.4998	0.0046	5	2	3	6	4	3	2	5
5328.5044	5328.4895	0.0149	5	2	3	4	4	3	2	3
5337.3153	5337.3152	0.0001	4	0	4	4	3	1	3	4
5338.4195	5338.4248	-0.0053	4	0	4	3	3	1	3	3
5433.5068	5433.5029	0.0039	3	2	2	3	2	1	1	2
5433.7435	5433.7426	0.0009	3	2	2	4	2	1	1	3
5433.8797	5433.8757	0.0040	3	2	2	2	2	1	1	1
5462.1892	5462.2020	-0.0128	4	1	4	4	3	1	3	3
5462.2659	5462.2612	0.0047	4	1	4	5	3	1	3	4
5544.7135	5544.7243	-0.0108	4	0	4	4	3	0	3	3
5544.8323	5544.8351	-0.0028	4	0	4	5	3	0	3	4
5668.7301	5668.7350	-0.0049	4	1	4	4	3	0	3	4
5669.0979	5669.1014	-0.0035	4	1	4	4	3	0	3	3
5669.3026	5669.3039	-0.0013	4	1	4	5	3	0	3	4
5669.8085	5669.8167	-0.0082	4	1	4	3	3	0	3	3
5682.3237	5682.3315	-0.0078	4	2	3	4	3	2	2	3
5682.4341	5682.4282	0.0059	4	2	3	5	3	2	2	4
5726.2179	5726.2276	-0.0097	4	3	2	4	3	3	1	3

5737.7350	5737.7356	-0.0006	4	3	1	5	3	3	0	4
5823.7608	5823.7536	0.0072	3	2	1	2	2	1	2	1
5824.0573	5824.0506	0.0067	3	2	1	4	2	1	2	3
5824.2343	5824.2248	0.0095	3	2	1	3	2	1	2	3
5824.4186	5824.4146	0.0040	3	2	1	2	2	1	2	2
5824.6530	5824.6498	0.0032	3	2	1	3	2	1	2	2
5833.3782	5833.3930	-0.0148	4	2	2	3	3	2	1	3
5833.6192	5833.6144	0.0048	4	2	2	5	3	2	1	4
5858.2986	5858.3017	-0.0031	4	1	3	3	3	1	2	3
6088.2696	6088.2834	-0.0138	6	2	5	6	5	3	2	5
6088.6293	6088.6199	0.0094	6	2	5	7	5	3	2	6
6432.4722	6432.4594	0.0128	5	1	4	6	4	2	3	5
6432.5907	6432.5892	0.0015	5	1	4	5	4	2	3	4
6455.0564	6455.0504	0.0060	3	3	1	2	2	2	0	2
6455.3995	6455.3992	0.0003	3	3	1	4	2	2	0	3
6455.6645	6455.6604	0.0041	3	3	1	3	2	2	0	3
6457.3381	6457.3320	0.0061	3	3	0	4	2	2	0	3
6474.6489	6474.6371	0.0118	3	3	0	2	2	2	1	2
6475.1891	6475.1832	0.0059	3	3	0	3	2	2	1	3
6674.8798	6674.8753	0.0045	4	1	3	5	3	0	3	4
6703.0095	6703.0118	-0.0023	4	2	3	4	3	1	2	3
6703.1298	6703.1334	-0.0036	4	2	3	3	3	1	2	3
6703.2889	6703.2819	0.0070	4	2	3	5	3	1	2	4
6737.7498	6737.7480	0.0018	5	0	5	5	4	1	4	5
6738.3210	6738.3166	0.0044	5	0	5	6	4	1	4	5
6738.9978	6739.0013	-0.0035	5	0	5	4	4	1	4	4
6805.7823	6805.7715	0.0108	5	1	5	6	4	1	4	5
6929.6187	6929.6227	-0.0040	5	1	5	5	4	0	4	5
6930.0936	6930.0999	-0.0063	5	1	5	5	4	0	4	4
6930.2364	6930.2403	-0.0039	5	1	5	6	4	0	4	5
6930.8311	6930.8433	-0.0122	5	1	5	4	4	0	4	4
6939.2141	6939.2066	0.0075	4	2	2	5	3	1	2	4
6973.8493	6973.8566	-0.0073	7	3	5	7	6	4	2	6
6974.0029	6974.0044	-0.0015	7	3	5	8	6	4	2	7
6995.3201	6995.3251	-0.0050	6	2	4	7	5	3	3	6
6995.4834	6995.4779	0.0055	6	2	4	6	5	3	3	5
7081.2319	7081.2467	-0.0148	5	2	4	6	4	2	3	5
7159.2705	7159.2778	-0.0073	5	4	2	6	4	4	1	5
7163.2409	7163.2573	-0.0164	5	3	3	6	4	3	2	5
7201.3138	7201.2942	0.0196	5	3	2	6	4	3	1	5
7236.0739	7236.0718	0.0021	7	2	6	7	6	3	3	6
7236.4816	7236.4783	0.0033	7	2	6	8	6	3	3	7
7277.2216	7277.2378	-0.0162	5	1	4	6	4	1	3	5
7307.2421	7307.2262	0.0159	7	3	4	8	6	4	3	7
7312.6210	7312.6111	0.0099	4	2	3	5	3	1	3	4
7337.3188	7337.3128	0.0060	5	2	3	6	4	2	2	5
7548.3172	7548.3102	0.0070	4	2	2	3	3	1	3	2
7548.5447	7548.5358	0.0089	4	2	2	5	3	1	3	4
7548.7289	7548.7193	0.0096	4	2	2	4	3	1	3	4
7549.0051	7548.9983	0.0068	4	2	2	3	3	1	3	3
7549.2335	7549.2290	0.0045	4	2	2	4	3	1	3	3
7842.2192	7842.2227	-0.0035	4	3	2	3	3	2	1	3
7842.4150	7842.4274	-0.0124	4	3	2	5	3	2	1	4
7855.7198	7855.7252	-0.0054	4	3	1	5	3	2	1	4
7925.7426	7925.7485	-0.0059	5	2	4	5	4	1	3	4

7926.0225	7926.0252	-0.0027	5	2	4	6	4	1	3	5
7927.1727	7927.1659	0.0068	4	3	2	5	3	2	2	4
7927.2842	7927.2842	0.0000	4	3	2	4	3	2	2	3
7940.4880	7940.4637	0.0243	4	3	1	5	3	2	2	4
7940.6144	7940.6075	0.0069	4	3	1	4	3	2	2	3

Table S28. Transition frequencies for the closed $\mathscr{C}$ switch form. J is the rotational angular momentum quantum number, K_a and K_c are the projections of J onto the principal axes at the prolate and oblate symmetric top limits, and F is the total angular momentum quantum number, which includes the nuclear spin, I(^{14}N) = 1.

Observed Frequency /MHz	Calculated Frequency /MHz	Difference / MHz	J	K_a	K_c	F	J'	K_a'	K_c'	F'
2743.3586	2743.3582	0.0004	2	1	2	2	1	1	1	2
2744.0570	2744.0524	0.0046	2	1	2	3	1	1	1	2
2744.3847	2744.3853	-0.0006	2	1	2	2	1	1	1	1
2745.4662	2745.4658	0.0004	2	1	2	1	1	1	1	1
2803.1195	2803.1127	0.0068	2	0	2	1	1	0	1	1
2803.5607	2803.5713	-0.0106	2	0	2	2	1	0	1	1
2803.6585	2803.6556	0.0029	2	0	2	3	1	0	1	2
2803.9511	2803.9503	0.0008	2	0	2	2	1	0	1	2
2804.0680	2804.0602	0.0078	2	0	2	1	1	0	1	0
2873.8307	2873.8303	0.0004	2	1	1	1	1	1	0	1
2875.0916	2875.0893	0.0023	2	1	1	3	1	1	0	2
2875.4377	2875.4499	-0.0122	2	1	1	1	1	1	0	0
2875.5463	2875.5420	0.0043	2	1	1	2	1	1	0	1
2876.1855	2876.1898	-0.0043	2	1	1	2	1	1	0	2
4111.8777	4111.8763	0.0014	3	1	3	3	2	1	2	3
4112.3671	4112.3657	0.0014	3	1	3	2	2	1	2	1
4112.5270	4112.5248	0.0022	3	1	3	4	2	1	2	3
4113.4526	4113.4463	0.0063	3	1	3	2	2	1	2	2
4190.5336	4190.5244	0.0092	3	0	3	2	2	0	2	2
4190.7018	4190.7118	-0.0100	3	0	3	3	2	0	2	2
4190.8681	4190.8676	0.0005	3	0	3	4	2	0	2	3
4190.9896	4190.9830	0.0066	3	0	3	2	2	0	2	1
4214.1637	4214.1529	0.0108	3	2	2	2	2	2	1	1
4214.3868	4214.3788	0.0080	3	2	2	4	2	2	1	3
4214.7960	4214.7850	0.0110	3	2	2	3	2	2	1	2
4237.6881	4237.7053	-0.0172	3	2	1	2	2	2	0	1
4237.9708	4237.9682	0.0026	3	2	1	4	2	2	0	3
4238.1532	4238.1645	-0.0113	3	2	1	2	2	2	0	2
4238.5374	4238.5454	-0.0080	3	2	1	3	2	2	0	2
4307.3309	4307.3276	0.0033	3	1	2	2	2	1	1	2
4308.8511	4308.8507	0.0004	3	1	2	4	2	1	1	3
4308.9674	4308.9580	0.0094	3	1	2	3	2	1	1	2
4310.0598	4310.0584	0.0014	3	1	2	3	2	1	1	3
4392.9401	4392.9517	-0.0116	2	2	0	2	1	1	0	1
4393.8992	4393.8941	0.0051	2	2	0	3	1	1	0	2
4453.2184	4453.2277	-0.0093	2	2	1	3	1	1	1	2
4453.8303	4453.8486	-0.0183	2	2	1	2	1	1	1	1
5476.4668	5476.4695	-0.0027	4	1	4	4	3	1	3	4
5477.1399	5477.1382	0.0017	4	1	4	5	3	1	3	4
5477.9626	5477.9589	0.0037	4	1	4	3	3	1	3	3

5562.0300	5562.0308	-0.0008	4	0	4	4	3	0	3	3
5562.2491	5562.2342	0.0149	4	0	4	5	3	0	3	4
5614.7352	5614.7296	0.0056	4	2	3	4	3	2	2	3
5632.0790	5632.0859	-0.0069	4	3	1	3	3	3	0	2
5632.2541	5632.2448	0.0093	4	3	1	5	3	3	0	4
5671.1752	5671.1741	0.0011	4	2	2	3	3	2	1	3
5671.6115	5671.6209	-0.0094	4	2	2	5	3	2	1	4
5671.9802	5671.9784	0.0018	4	2	2	4	3	2	1	3
5672.2588	5672.2609	-0.0021	4	2	2	4	3	2	1	4
5735.8460	5735.8429	0.0031	4	1	3	3	3	1	2	3
5737.3655	5737.3626	0.0029	4	1	3	5	3	1	2	4
5737.4838	5737.4733	0.0105	4	1	3	3	3	1	2	2
5738.5802	5738.5761	0.0041	4	1	3	4	3	1	2	4
5755.9643	5755.9551	0.0092	3	2	1	3	2	1	1	2
5756.7761	5756.7730	0.0031	3	2	1	4	2	1	1	3
5757.2831	5757.2859	-0.0028	3	2	1	2	2	1	1	1
5923.1784	5923.1681	0.0103	3	2	2	2	2	1	2	1
5923.5503	5923.5541	-0.0038	3	2	2	4	2	1	2	3
5924.2496	5924.2483	0.0013	3	2	2	3	2	1	2	2
6836.6102	6836.6195	-0.0093	5	1	5	5	4	1	4	5
6838.1432	6838.1481	-0.0049	5	1	5	4	4	1	4	4
6916.7731	6916.7789	-0.0058	5	0	5	5	4	0	4	4
7010.6052	7010.6156	-0.0104	5	2	4	4	4	2	3	4
7010.7886	7010.7989	-0.0103	5	2	4	6	4	2	3	5
7011.0014	7010.9896	0.0118	5	2	4	5	4	2	3	5
7041.2041	7041.2179	-0.0138	5	3	3	4	4	3	2	3
7041.5077	7041.4987	0.0090	5	3	3	5	4	3	2	4
7048.3531	7048.3530	0.0001	5	3	2	6	4	3	1	5
7048.6149	7048.6188	-0.0039	5	3	2	5	4	3	1	4
7116.9604	7116.9591	0.0013	5	2	3	4	4	2	2	4
7117.7831	7117.7844	-0.0013	5	2	3	6	4	2	2	5
7118.0572	7118.0550	0.0022	5	2	3	5	4	2	2	4
7118.6877	7118.6950	-0.0073	5	2	3	5	4	2	2	5
7156.6627	7156.6605	0.0022	5	1	4	4	4	1	3	4
7159.2705	7159.2724	-0.0019	5	1	4	5	4	1	3	5
7226.1071	7226.1118	-0.0047	6	1	5	7	5	2	3	6
7226.2616	7226.2650	-0.0034	6	1	5	6	5	2	3	5
7425.6147	7425.6142	0.0005	4	2	3	5	3	1	3	4
7426.4202	7426.4075	0.0127	4	2	3	4	3	1	3	3
7878.5431	7878.5465	-0.0034	4	2	2	3	3	0	3	2

Table S29. Transition frequencies for the closed $\mathscr{C}$ switch form: ^{13}C(1). J is the rotational angular momentum quantum number, K_a and K_c are the projections of J onto the principal axes at the prolate and oblate symmetric top limits, and F is the total angular momentum quantum number, which includes the nuclear spin, I(^{14}N) = 1.

Observed Frequency /MHz	Calculated Frequency /MHz	Difference / MHz	J	K_a	K_c	F	J'	K'_a	K'_c	F'
4099.6624	4099.6730	-0.0106	3	1	3	2	2	1	2	1
4099.8448	4099.8320	0.0128	3	1	3	4	2	1	2	3
4176.5087	4176.5031	0.0056	3	0	3	3	2	0	2	2
4176.6491	4176.6588	-0.0097	3	0	3	4	2	0	2	3
4292.3037	4292.3101	-0.0064	3	1	2	4	2	1	1	3

4292.4384	4292.4174	0.0210	3	1	2	3	2	1	1	2
5460.3408	5460.3413	-0.0005	4	1	4	5	3	1	3	4
5543.6113	5543.6055	0.0058	4	0	4	4	3	0	3	3
5543.8238	5543.8088	0.0150	4	0	4	5	3	0	3	4
5595.2385	5595.2259	0.0126	4	2	3	4	3	2	2	3
5650.9369	5650.9541	-0.0172	4	2	2	5	3	2	1	4
5715.4698	5715.4691	0.0007	4	1	3	5	3	1	2	4
5715.5859	5715.5798	0.0061	4	1	3	3	3	1	2	2
6816.5381	6816.5216	0.0165	5	1	5	6	4	1	4	5
6894.4567	6894.4659	-0.0092	5	0	5	5	4	0	4	4
6894.6637	6894.6849	-0.0212	5	0	5	6	4	0	4	5
6986.5583	6986.5718	-0.0135	5	2	4	6	4	2	3	5
7023.3512	7023.3376	0.0136	5	3	2	6	4	3	1	5
7023.5980	7023.6033	-0.0053	5	3	2	5	4	3	1	4
7091.3828	7091.3871	-0.0043	5	2	3	6	4	2	2	5
7091.6536	7091.6577	-0.0041	5	2	3	5	4	2	2	4
7130.9744	7130.9725	0.0019	5	1	4	5	4	1	3	4

Table S30. Transition frequencies for the closed $\mathscr{C}$ switch form: ^{13}C(2). J is the rotational angular momentum quantum number, K_a and K_c are the projections of J onto the principal axes at the prolate and oblate symmetric top limits, and F is the total angular momentum quantum number, which includes the nuclear spin, I(^{14}N) = 1.

Observed Frequency /MHz	Calculated Frequency /MHz	Difference / MHz	J	K_a	K_c	F	J'	K'_a	K'_c	F'
4101.7341	4101.7316	0.0025	3	1	3	2	2	1	2	1
4101.8847	4101.8911	-0.0064	3	1	3	4	2	1	2	3
4176.9604	4176.9522	0.0082	3	0	3	3	2	0	2	2
4177.1018	4177.1019	-0.0001	3	0	3	4	2	0	2	3
4288.6286	4288.6260	0.0026	3	1	2	4	2	1	1	3
5463.4591	5463.4644	-0.0053	4	1	4	5	3	1	3	4
5545.7034	5545.7091	-0.0057	4	0	4	4	3	0	3	3
5545.9007	5545.9062	-0.0055	4	0	4	5	3	0	3	4
5594.1303	5594.1280	0.0023	4	2	3	4	3	2	2	3
5646.3388	5646.3504	-0.0116	4	2	2	5	3	2	1	4
5711.0906	5711.0906	0.0000	4	1	3	5	3	1	2	4
5711.2042	5711.2007	0.0035	4	1	3	3	3	1	2	2
6820.9573	6820.9450	0.0123	5	1	5	6	4	1	4	5
6898.8940	6898.9071	-0.0131	5	0	5	5	4	0	4	4
6899.1750	6899.1632	0.0118	5	0	5	4	4	0	4	3
6985.6734	6985.6777	-0.0043	5	2	4	6	4	2	3	5
7013.6724	7013.6645	0.0079	5	3	3	6	4	3	2	5
7013.8969	7013.8939	0.0030	5	3	3	5	4	3	2	4
7019.8482	7019.8520	-0.0038	5	3	2	4	4	3	1	3
7020.1658	7020.1706	-0.0048	5	3	2	5	4	3	1	4
7084.2978	7084.3112	-0.0134	5	2	3	6	4	2	2	5
7084.5973	7084.5795	0.0178	5	2	3	5	4	2	2	4
7126.4625	7126.4620	0.0005	5	1	4	5	4	1	3	4

Table S31. Transition frequencies for the closed $\mathscr{C}$ switch form: ^{13}C(3). J is the rotational angular momentum quantum number, K_a and K_c are the projections of J onto the principal axes at the prolate and oblate symmetric top limits, and F is the total angular momentum quantum number, which includes the nuclear spin, I(^{14}N) = 1.

Observed Frequency /MHz	Calculated Frequency /MHz	Difference / MHz	J	K_a	K_c	F	J'	K'_a	K'_c	F'
4184.4148	4184.4250	-0.0102	3	0	3	4	2	0	2	3
4184.5259	4184.5406	-0.0147	3	0	3	2	2	0	2	1
4303.1720	4303.1709	0.0011	3	1	2	4	2	1	1	3
5468.1132	5468.1051	0.0081	4	1	4	5	3	1	3	4
5553.1586	5553.1515	0.0071	4	0	4	4	3	0	3	3
5553.3726	5553.3560	0.0166	4	0	4	5	3	0	3	4
5606.4699	5606.4670	0.0029	4	2	3	4	3	2	2	3
5664.0284	5664.0322	-0.0038	4	2	2	5	3	2	1	4
5664.3974	5664.3905	0.0069	4	2	2	4	3	2	1	3
5729.6832	5729.6858	-0.0026	4	1	3	5	3	1	2	4
5729.8028	5729.7966	0.0062	4	1	3	3	3	1	2	2
6905.3382	6905.3399	-0.0017	5	0	5	5	4	0	4	4
6905.5516	6905.5597	-0.0081	5	0	5	6	4	0	4	5
7000.3694	7000.3783	-0.0089	5	2	4	6	4	2	3	5
7038.4250	7038.4278	-0.0028	5	3	2	6	4	3	1	5
7038.6972	7038.6942	0.0030	5	3	2	5	4	3	1	4
7108.5404	7108.5433	-0.0029	5	2	3	6	4	2	2	5
7108.8144	7108.8144	0.0000	5	2	3	5	4	2	2	4
7148.2778	7148.2752	0.0026	5	1	4	5	4	1	3	4

Table S32. Transition frequencies for the closed $\mathscr{C}$ switch form: ^{13}C(4). J is the rotational angular momentum quantum number, K_a and K_c are the projections of J onto the principal axes at the prolate and oblate symmetric top limits, and F is the total angular momentum quantum number, which includes the nuclear spin, I(^{14}N) = 1.

Observed Frequency /MHz	Calculated Frequency /MHz	Difference / MHz	J	K_a	K_c	F	J'	K'_a	K'_c	F'
4107.3277	4107.3245	0.0032	3	1	3	2	2	1	2	1
4107.4743	4107.4834	-0.0091	3	1	3	4	2	1	2	3
4186.1108	4186.1052	0.0056	3	0	3	3	2	0	2	2
4186.2482	4186.2627	-0.0145	3	0	3	4	2	0	2	3
4186.3680	4186.3785	-0.0105	3	0	3	2	2	0	2	1
4305.4171	4305.4256	-0.0085	3	1	2	4	2	1	1	3
4305.5353	4305.5324	0.0029	3	1	2	3	2	1	1	2
4305.6204	4305.6144	0.0060	3	1	2	2	2	1	1	1
5470.3104	5470.3052	0.0052	4	1	4	5	3	1	3	4
5555.6590	5555.6529	0.0061	4	0	4	5	3	0	3	4
5555.6728	5555.6707	0.0021	4	0	4	3	3	0	3	4
5608.9585	5608.9473	0.0112	4	2	3	5	3	2	2	4
5609.1114	5609.0917	0.0197	4	2	3	4	3	2	2	3
5667.0091	5667.0157	-0.0066	4	2	2	5	3	2	1	4
5667.3889	5667.3746	0.0143	4	2	2	4	3	2	1	3
5732.6306	5732.6369	-0.0063	4	1	3	5	3	1	2	4
5732.7390	5732.7478	-0.0088	4	1	3	3	3	1	2	2
7003.5961	7003.6100	-0.0139	5	2	4	6	4	2	3	5
7034.6127	7034.6276	-0.0149	5	3	3	6	4	3	2	5

7034.8599	7034.8580	0.0019	5	3	3	5	4	3	2	4
7112.4000	7112.4041	-0.0041	5	2	3	6	4	2	2	5
7112.6796	7112.6754	0.0042	5	2	3	5	4	2	2	4
7151.9299	7151.9174	0.0125	5	1	4	6	4	1	3	5

Table S33. Transition frequencies for the closed $\mathscr{C}$ switch form: ^{13}C(5). J is the rotational angular momentum quantum number, K_a and K_c are the projections of J onto the principal axes at the prolate and oblate symmetric top limits, and F is the total angular momentum quantum number, which includes the nuclear spin, I(^{14}N) = 1.

Observed Frequency /MHz	Calculated Frequency /MHz	Difference / MHz	J	K_a	K_c	F	J'	K'_a	K'_c	F'
4104.9036	4104.8905	0.0131	3	1	3	4	2	1	2	3
4183.1653	4183.1714	-0.0061	3	0	3	3	2	0	2	2
4183.3250	4183.3274	-0.0024	3	0	3	4	2	0	2	3
4183.4299	4183.4428	-0.0129	3	0	3	2	2	0	2	1
4301.5130	4301.5027	0.0103	3	1	2	4	2	1	1	3
4301.6143	4301.6099	0.0044	3	1	2	3	2	1	1	2
5466.9482	5466.9443	0.0039	4	1	4	5	3	1	3	4
5551.9150	5551.9176	-0.0026	4	0	4	4	3	0	3	3
5552.1339	5552.1211	0.0128	4	0	4	5	3	0	3	4
5604.7564	5604.7428	0.0136	4	2	3	4	3	2	2	3
5661.7619	5661.7713	-0.0094	4	2	2	5	3	2	1	4
5662.1309	5662.1289	0.0020	4	2	2	4	3	2	1	3
5727.5471	5727.5448	0.0023	4	1	3	5	3	1	2	4
5727.6700	5727.6555	0.0145	4	1	3	3	3	1	2	2
6904.0699	6904.0634	0.0065	5	0	5	5	4	0	4	4
6904.2653	6904.2826	-0.0173	5	0	5	6	4	0	4	5
6998.2898	6998.2970	-0.0072	5	2	4	6	4	2	3	5
7028.8507	7028.8407	0.0100	5	3	3	6	4	3	2	5
7029.0885	7029.0710	0.0175	5	3	3	5	4	3	2	4
7035.9321	7035.9501	-0.0180	5	3	2	6	4	3	1	5
7036.2069	7036.2160	-0.0091	5	3	2	5	4	3	1	4
7105.5248	7105.5274	-0.0026	5	2	3	6	4	2	2	5
7105.7975	7105.7981	-0.0006	5	2	3	5	4	2	2	4
7145.7373	7145.7500	-0.0127	5	1	4	5	4	1	3	4

Table S34. Transition frequencies for the closed $\mathscr{C}$ switch form: ^{13}C(6). J is the rotational angular momentum quantum number, K_a and K_c are the projections of J onto the principal axes at the prolate and oblate symmetric top limits, and F is the total angular momentum quantum number, which includes the nuclear spin, I(^{14}N) = 1.

Observed Frequency /MHz	Calculated Frequency /MHz	Difference / MHz	J	K_a	K_c	F	J'	K'_a	K'_c	F'
4184.0791	4184.0837	-0.0046	3	0	3	4	2	0	2	3
5551.5455	5551.5359	0.0096	4	0	4	4	3	0	3	3
5551.7633	5551.7450	0.0183	4	0	4	5	3	0	3	4
5607.2117	5607.1912	0.0205	4	2	3	5	3	2	2	4
5607.3441	5607.3349	0.0092	4	2	3	4	3	2	2	3
5667.5986	5667.6143	-0.0157	4	2	2	5	3	2	1	4

5667.9838	5667.9764	0.0074	4	2	2	4	3	2	1	3
5732.8402	5732.8454	-0.0052	4	1	3	5	3	1	2	4
5732.9511	5732.9567	-0.0056	4	1	3	3	3	1	2	2
6901.9662	6901.9716	-0.0054	5	0	5	5	4	0	4	4
6902.1752	6902.1943	-0.0191	5	0	5	6	4	0	4	5
7001.0821	7001.0895	-0.0074	5	2	4	6	4	2	3	5
7113.9793	7113.9856	-0.0063	5	2	3	6	4	2	2	5
7114.2613	7114.2582	0.0031	5	2	3	5	4	2	2	4
7151.5247	7151.5179	0.0068	5	1	4	6	4	1	3	5

Table S35. Transition frequencies for the closed $\mathscr{C}$ switch form: ^{13}C(7). J is the rotational angular momentum quantum number, K_a and K_c are the projections of J onto the principal axes at the prolate and oblate symmetric top limits, and F is the total angular momentum quantum number, which includes the nuclear spin, I(^{14}N) = 1.

Observed Frequency /MHz	Calculated Frequency /MHz	Difference / MHz	J	K_a	K_c	F	J'	K'_a	K'_c	F'
4161.6677	4161.6588	0.0089	3	0	3	3	2	0	2	2
4161.8274	4161.8255	0.0019	3	0	3	4	2	0	2	3
4287.1966	4287.1958	0.0008	3	1	2	4	2	1	1	3
4287.3008	4287.3002	0.0006	3	1	2	3	2	1	1	2
5520.4985	5520.4934	0.0051	4	0	4	4	3	0	3	3
5520.7224	5520.7076	0.0148	4	0	4	5	3	0	3	4
5642.7083	5642.7095	-0.0012	4	2	2	5	3	2	1	4
5643.0697	5643.0758	-0.0061	4	2	2	4	3	2	1	3
5707.4545	5707.4636	-0.0091	4	1	3	5	3	1	2	4
5707.5699	5707.5754	-0.0055	4	1	3	3	3	1	2	2
6861.6046	6861.5999	0.0047	5	0	5	5	4	0	4	4
6861.8066	6861.8257	-0.0191	5	0	5	6	4	0	4	5
6965.5096	6965.5081	0.0015	5	2	4	6	4	2	3	5
7007.9512	7007.9581	-0.0069	5	3	2	6	4	3	1	5
7008.2243	7008.2302	-0.0059	5	3	2	5	4	3	1	4
7083.9791	7083.9667	0.0124	5	2	3	6	4	2	2	5
7118.8927	7118.8852	0.0075	5	1	4	6	4	1	3	5

Table S36. Transition frequencies for the closed $\mathscr{C}$ switch form: ^{13}C(8). J is the rotational angular momentum quantum number, K_a and K_c are the projections of J onto the principal axes at the prolate and oblate symmetric top limits, and F is the total angular momentum quantum number, which includes the nuclear spin, I(^{14}N) = 1.

Observed Frequency /MHz	Calculated Frequency /MHz	Difference / MHz	J	K_a	K_c	F	J'	K'_a	K'_c	F'
4103.1361	4103.1285	0.0076	3	1	3	4	2	1	2	3
4181.0404	4181.0499	-0.0095	3	0	3	4	2	0	2	3
4204.6339	4204.6336	0.0003	3	2	2	3	2	2	1	2
4298.0620	4298.0587	0.0033	3	1	2	4	2	1	1	3
4298.1625	4298.1663	-0.0038	3	1	2	3	2	1	1	2
4298.2602	4298.2473	0.0129	3	1	2	2	2	1	1	1
5464.6817	5464.6926	-0.0109	4	1	4	5	3	1	3	4
5549.2639	5549.2721	-0.0082	4	0	4	4	3	0	3	3

5549.4737	5549.4744	-0.0007	4	0	4	5	3	0	3	4
5601.1297	5601.1154	0.0143	4	2	3	5	3	2	2	4
5601.2737	5601.2603	0.0134	4	2	3	4	3	2	2	3
5657.3682	5657.3840	-0.0158	4	2	2	5	3	2	1	4
5657.7487	5657.7405	0.0082	4	2	2	4	3	2	1	3
5723.0896	5723.0894	0.0002	4	1	3	5	3	1	2	4
5723.2114	5723.1999	0.0115	4	1	3	3	3	1	2	2
6821.8946	6821.8910	0.0036	5	1	5	6	4	1	4	5
6901.5026	6901.4916	0.0110	5	0	5	4	4	0	4	3
6994.0504	6994.0667	-0.0163	5	2	4	6	4	2	3	5
7031.0632	7031.0542	0.0090	5	3	2	6	4	3	1	5
7031.3088	7031.3193	-0.0105	5	3	2	5	4	3	1	4
7099.6897	7099.6938	-0.0041	5	2	3	6	4	2	2	5
7099.9715	7099.9641	0.0074	5	2	3	5	4	2	2	4
7140.4099	7140.4261	-0.0162	5	1	4	5	4	1	3	4

Table S37. Transition frequencies for the closed $\mathscr{C}$ switch form: ^{13}C(9). J is the rotational angular momentum quantum number, K_a and K_c are the projections of J onto the principal axes at the prolate and oblate symmetric top limits, and F is the total angular momentum quantum number, which includes the nuclear spin, I(^{14}N) = 1.

Observed Frequency /MHz	Calculated Frequency /MHz	Difference / MHz	J	K_a	K_c	F	J'	K'_a	K'_c	F'
4101.4606	4101.4541	0.0065	3	1	3	4	2	1	2	3
4283.6870	4283.6840	0.0030	3	1	2	4	2	1	1	3
4283.7855	4283.7935	-0.0080	3	1	2	3	2	1	1	2
4283.8693	4283.8719	-0.0026	3	1	2	2	2	1	1	1
5640.7094	5640.7185	-0.0091	4	2	2	5	3	2	1	4
5641.0745	5641.0685	0.0060	4	2	2	4	3	2	1	3
5704.7973	5704.7994	-0.0021	4	1	3	5	3	1	2	4
5704.9125	5704.9093	0.0032	4	1	3	3	3	1	2	2
6820.7614	6820.7580	0.0034	5	1	5	5	4	1	4	4
6897.8705	6897.8740	-0.0035	5	0	5	5	4	0	4	4
6898.0820	6898.0870	-0.0050	5	0	5	6	4	0	4	5
6981.4527	6981.4539	-0.0012	5	2	4	6	4	2	3	5
7008.5949	7008.5900	0.0049	5	3	3	5	4	3	2	4
7014.2532	7014.2580	-0.0048	5	3	2	6	4	3	1	5
7014.5198	7014.5190	0.0008	5	3	2	5	4	3	1	4
7076.4136	7076.4170	-0.0034	5	2	3	6	4	2	2	5
7076.6852	7076.6842	0.0010	5	2	3	5	4	2	2	4
7119.1856	7119.1756	0.0100	5	1	4	6	4	1	3	5

Table S38. Transition frequencies for the closed $\mathscr{C}$ switch form: ^{13}C(10). J is the rotational angular momentum quantum number, K_a and K_c are the projections of J onto the principal axes at the prolate and oblate symmetric top limits, and F is the total angular momentum quantum number, which includes the nuclear spin, I(^{14}N) = 1.

Observed Frequency /MHz	Calculated Frequency /MHz	Difference / MHz	J	K_a	K_c	F	J'	K'_a	K'_c	F'
4069.7791	4069.7696	0.0095	3	1	3	4	2	1	2	3
4147.3651	4147.3683	-0.0032	3	0	3	3	2	0	2	2
4147.5357	4147.5210	0.0147	3	0	3	4	2	0	2	3
4170.3070	4170.2993	0.0077	3	2	2	4	2	2	1	3
4263.6786	4263.6857	-0.0071	3	1	2	4	2	1	1	3
5420.3007	5420.3144	-0.0137	4	1	4	5	3	1	3	4
5504.9536	5504.9649	-0.0113	4	0	4	4	3	0	3	3
5555.9707	5555.9595	0.0112	4	2	3	5	3	2	2	4
5556.1232	5556.1048	0.0184	4	2	3	4	3	2	2	3
5611.2794	5611.2943	-0.0149	4	2	2	5	3	2	1	4
5611.6539	5611.6491	0.0048	4	2	2	4	3	2	1	3
5677.4141	5677.4055	0.0086	4	1	3	5	3	1	2	4
5677.5273	5677.5159	0.0114	4	1	3	3	3	1	2	2
6766.5700	6766.5534	0.0166	5	1	5	6	4	1	4	5
6846.2995	6846.2975	0.0020	5	0	5	5	4	0	4	4
6846.4996	6846.5145	-0.0149	5	0	5	6	4	0	4	5
6937.7319	6937.7516	-0.0197	5	2	4	6	4	2	3	5
6967.3058	6967.3173	-0.0115	5	3	3	6	4	3	2	5
6967.5604	6967.5471	0.0133	5	3	3	5	4	3	2	4
6974.0483	6974.0486	-0.0003	5	3	2	6	4	3	1	5
7041.7559	7041.7651	-0.0092	5	2	3	6	4	2	2	5
7083.5881	7083.5889	-0.0008	5	1	4	5	4	1	3	4

2.4 Relative abundances

Table S39. Relative abundance of the $\mathscr{C}$ and $\mathscr{O}$ switch forms, obtained with different carrier gases.

Carrier gas	$\mathscr{O}$	$\mathscr{C}$	$\mathscr{O}/\mathscr{C}$
Helium	71(13)%	29.0(50)%	2.40
Neon	72(13)%	28.0(48)%	2.55
Argon	74(14)%	26.0(44)%	2.90

3 The dynamics of micro-hydrated species

Table S40. Experimental and calculated (DLPNO-SCS-MP2/def2-TZVP) spectroscopic constants for the observed micro-hydrated species. The theoretical constants are presented for minimum equilibrium structures (M), or for transition-state geometries (TS). The A,B,C parameters are the rotational constants; $\Delta_J, \Delta_{JK}, \delta_J, \delta_K$ are the quartic centrifugal distortion constants; and $\chi_{aa}, \chi_{bb} - \chi_{cc}$ are the nuclear quadrupole coupling constants associated to the ^{14}N atom. The predicted dipole moment components, μ, for the a-, b-, and c-type transitions, and the corresponding number of assigned lines is shown in parentheses. The standard error for each fit, σ, and the theoretical zero-point corrected relative energy, ΔE_{ZPC} are also shown. All theoretical parameters are expressed in the PAS.

	$\mathcal{O}_{1w}$		$\mathcal{C}_{1w}$	
Constants	Experimental	Theory (M$_1$ / M$_2$ / TS)	Experimental	Theory (M)
A /MHz	1118.05985(48)	1120 / 1123 / 1120	878.82397(26)	878
B /MHz	455.52043(19)	454 / 454 / 457	597.71561(28)	600
C /MHz	414.16275(21)	414 / 413 / 415	468.60872(26)	468
Δ_J /kHz	0.0167(16)	–	0.0918(27)	–
Δ_{JK} /kHz	0.1489(80)	–	–	–
δ_J / kHz	–	–	0.0307(15)	–
χ_{aa} /MHz	-2.246(12)	-2.7 / -2.7 / -2.8	-4.3136(47)	-5.1
$\chi_{bb} - \chi_{cc}$ /MHz	-0.828(16)	-2.6 / -2.5 / -2.4	0.4685(80)	-1.2
μ_a /D	yes (45)	-1.6 / -1.8 / -1.3	yes (149)	-3.0
μ_b /D	yes (97)	1.4 / 1.5 / 1.0	yes (60)	1.2
μ_c /D	no	1.3 / -1.1 / 0.2	no	0.4
σ /kHz	9.39	–	10.45	–
ΔE_{ZPC} /kJ mol^{-1}	–	0 / +0.2 / +0.3	–	+10.1

	$\mathcal{O}_{2w}$		$\mathcal{O}_{3w}$		$\mathcal{O}_{4w}$	
Constants	Experimental	Theory (M$_1$ / M$_2$ / TS)	Experimental	Theory (M$_1$)	Experimental	Theory (M$_1$)
A /MHz	960.88481(54)	966 / 966 / 963	846.3302(25)	842	715.902(49)	726
B /MHz	357.10552(30)	353 / 353 / 356	263.67614(17)	264	222.13527(20)	219
C /MHz	316.23806(26)	314 / 314 / 315	233.53832(17)	233	212.28583(19)	208
Δ_J /kHz	0.0213(12)	–	0.01265(35)	–	0.01088(40)	–
Δ_{JK} /kHz	–	–	0.0161(80)	–	–	–
δ_K / kHz	0.227(57)	–	–	–	0.144(75)	–
δ_J / kHz	0.00470(59)	–	–	–	–	–
χ_{aa} /MHz	-2.845(25)	-3.3 / -3.3 / -3.5	-3.097(80)	-4.0	–	-4.1
$\chi_{bb} - \chi_{cc}$ /MHz	-0.721(21)	-2.5 / -2.5 / -2.3	0.42(26)	-1.9	–	-2.1
μ_a /D	yes (51)	-2.4 / -2.4 / -1.9	yes (102)	-2.8	yes (155)	3.9
μ_b /D	yes (93)	0.9 / -0.9 / -1.1	yes (4)	0.9	no	-1.7
μ_c /D	no	-0.2 / 0.5 / -0.9	no	-0.3	no	-1.4
σ /kHz	10.45	–	9.63	–	11.63	–
ΔE_{ZPC} /kJ mol^{-1}	–	0 / +0.5 / +1.3	–	0	–	0

3.1 Singly-substituted ^{18}O isopotologues

Table S41. Experimental spectroscopic constants for the observed singly-substituted ^{18}O micro-hydrated species. The A,B,C parameters are the rotational constants; $\Delta_J, \Delta_{JK}, \Delta_K, \delta_J, \delta_K$, the quartic centrifugal distortion constants; and $\chi_{aa}, \chi_{bb}-\chi_{cc}$, the nuclear quadrupole coupling constants associated to the ^{14}N atom. The corresponding number of assigned lines is shown in parentheses. The standard error of each fit, σ is also shown.

Constants	$\mathscr{O}_{1w}$: O(29)	$\mathscr{C}_{1w}$: O(29)	$\mathscr{O}_{2w}$: O(29)	$\mathscr{O}_{2w}$: O(32)	$\mathscr{O}_{3w}$: O(29)	$\mathscr{O}_{3w}$: O(32)	$\mathscr{O}_{3w}$: O(35)
A /MHz	1117.2898(12)	868.64250(47)	950.6133(82)	958.4518(15)	844.616(23)	836.530(20)	838.497(35)
B /MHz	437.64699(26)	580.36365(60)	350.46963(43)	346.33702(34)	256.08774(17)	261.40269(27)	258.79107(34)
C /MHz	399.27458(27)	455.09543(52)	309.95019(34)	307.52759(32)	227.45535(18)	231.05314(26)	229.13396(37)
Δ_J /KHz	0.0090(18)	0.0932(39)	0.01368(77)	0.01369(64)	0.01509(40)	0.01222(87)	0.01221(68)
δ_J /KHz	–	0.0273(32)	0.00192(85)	0.00285(75)	–	–	–
χ_{aa} /MHz	-2.154(44)	-4.632(12)	-2.836(47)	-2.698(54)	-2.84(28)	[-2.91]	[-2.91]
$\chi_{bb}-\chi_{cc}$ /MHz	-0.652(45)	0.737(48)	-0.35(12)	-0.07(12)	2.18(51)	[0.86]	[0.86]
μ_a /D	yes (76)	yes (149)	yes (113)	yes (118)	yes (35)	yes (26)	yes (25)
μ_b /D	yes (26)	yes (60)	yes(3)	no	no	no	no
μ_c /D	no	no	no	yes (3)	no	no	no
σ /KHz	10.23	10.98	8.96	8.99	4.98	7.64	9.67
a /Å	4.7743(3)	3.5247(4)	3.6596(5)	4.7018(3)	5.3402(5)	2.8713(9)	4.253(9)
b /Å	0.368(4)	1.9275(8)	1.712(1)	0.83(2)	0.776(4)	1.861(2)	1.686(2)
c /Å	0.180(8)	0.03(5)	0.12(1)	0.14(1)	0.19(2)	0.320(9)	0.21(2)

3.2 Clusters with 1 water molecule

Table S42. Kraitchman Analysis for $\mathscr{O}_{1w}$ (top) and $\mathscr{C}_{1w}$ (bottom). The experimental coordinates obtained through the Kraitchman equations, a, b, c are represented in their absolute value. The theoretical coordinates predicted for the different energy minima (M), transition-state (TS) and midpoints are also presented as well as the corresponding absolute deviation from the experimental value. Theoretical coordinates are presented in the Principal Axis System.

Coordinate	$\mathscr{O}_{1w}$ Experimental	$\mathscr{O}_{1w}$ (M$_1$)		$\mathscr{O}_{1w}$ (M$_2$)		$\mathscr{O}_{1w}$ (TS)		$\mathscr{O}_{1w}$ Midpoint (M$_1$,M$_2$)	
	Module	Value	Deviation	Value	Deviation	Value	Deviation	Value	Deviation
a /Å	| 4.77431(3) |	4.79852	0.02421	4.81432	0.04001	4.75925	0.01506	4.80642	0.03211
b /Å	| 0.368(4) |	-0.3595	0.0083	-0.3666	0.0012	-0.3755	0.0076	-0.3631	0.0048
c /Å	| 0.180(8) |	-0.2622	0.082	-0.127	0.053	-0.177	0.0035	-0.194	0.014

Coordinate	$\mathscr{C}_{1w}$ Experimental	$\mathscr{C}_{1w}$ (M))	
	Module	Value	Deviation
a /Å	| 3.5247(4) |	3.5348	0.0101
b /Å	| 1.9275(8) |	1.9266	0.0009
c /Å	| 0.03(5) |	-0.03	0.00

3.2.1 Equilibrium and transition state geometries

Table S43. Equilibrium geometry of $\mathscr{O}_{1w}$ (M_1).

Atom	x /Å	y /Å	z /Å
C	-0.77705276762886	1.09084842380116	1.68549225850803
C	-1.60928593741979	-0.22326162713493	1.66350290525467
C	-1.54503312757628	-0.70610274428756	0.18381403893784
C	-0.10803273940775	-1.19792301782025	0.03171640810233
C	0.71246438832993	0.06625044748836	0.08083061719249
C	-0.26777617808304	1.19625232536920	0.22703673062931
C	-2.59363577725654	-1.72377169318514	-0.20561153923486
C	-1.46583931784709	0.64344570743569	-0.59691097132704
C	-1.13051810580489	0.46556299341979	-2.08082729006329
C	-2.72738183252308	1.49932955106258	-0.48641542987481
O	0.28168911876308	-2.33966271661829	-0.08563104253592
N	1.99167068230174	0.03811957188657	0.02967403691818
O	2.55002610535234	1.30729319225055	0.11631536416966
H	-1.39176268246247	1.95510488434446	1.94526238387153
H	0.05245833752134	1.05304118150491	2.39493260472701
H	-1.21412938100688	-0.98374488092501	2.34319232667688
H	-2.65089801381056	-0.05055596695381	1.94643182526460
H	0.11695610520325	2.17304256580040	-0.06681430903386
H	-2.49416584831862	-2.62273643540499	0.40814430397350
H	-3.60006908476447	-1.32094040099717	-0.06300428924436
H	-2.48344807116271	-2.02814952296681	-1.24950725521290
H	-0.24041105547009	-0.14790910856227	-2.24719451330450
H	-1.96397251869919	-0.00565193425950	-2.60956103854357
H	-0.95073554919888	1.44035989009987	-2.54468180476322
H	-3.07223077178612	1.63590584177725	0.53905046520266
H	-2.54102405856076	2.49084943210065	-0.91152477111699
H	-3.54393507980257	1.04537160758482	-1.05529543328488
H	3.50210845536079	1.09985137725008	0.04223876686947
O	4.79510459181739	-0.30376903875529	-0.27637398003042
H	4.01348234930464	-0.87773601802407	-0.25969186169545
H	5.41447776463620	-0.69628388728130	0.34794049296789

Table S44. Equilibrium geometry of $\mathscr{O}_{1w}$ (M_2).

Atom	x /Å	y /Å	z /Å
C	-0.73890350973799	1.07833262512672	1.66450883624878
C	-1.58624013910922	-0.22626867107608	1.65760863318202
C	-1.55763107058738	-0.70775276140037	0.17638641771489
C	-0.13013391863858	-1.21585911891729	-0.00739710389533
C	0.70641227982997	0.03819225326803	0.03277935745103
C	-0.25777257803721	1.17968787540931	0.19601927604584
C	-2.62588376973029	-1.71223657296377	-0.19338066687750
C	-1.47822764065164	0.64203378323320	-0.60424999457480
C	-1.17466502158485	0.46239765472161	-2.09478970218492
C	-2.72751721886048	1.51197620403429	-0.46781803831155
O	0.24308233353098	-2.36087953008941	-0.14454023496980
N	1.98471782181568	-0.00576380302150	-0.02824961020411
O	2.55888385943594	1.25711649009944	0.05069178345731
H	-1.33839192317286	1.94919146995586	1.93735872266305
H	0.10431585860190	1.03010860718876	2.35681532093792
H	-1.18583505573796	-0.99195877900669	2.32822896492597
H	-2.61987862251406	-0.04226436145414	1.96177628399473
H	0.13261563833669	2.15234217430920	-0.10395774602856
H	-2.52476293632293	-2.61331812334904	0.41694511475269
H	-3.62437188828409	-1.29779906666637	-0.02990275020060
H	-2.54037103253807	-2.01622096927705	-1.23970310611816
H	-0.29581344210334	-0.16211857893290	-2.27911043507606
H	-2.02405244479082	0.00236053749737	-2.60776349418666
H	-0.99200508731739	1.43569619054303	-2.56071433920619
H	-3.04871260048495	1.65291964855851	0.56465917266079
H	-2.53927108137544	2.50115138428276	-0.89753539220095
H	-3.56103469090481	1.06679600624764	-1.01873593510198
H	3.50838329458716	1.03700174989996	-0.02028718586167
O	4.79971526342737	-0.39949291356923	-0.16672038921715
H	4.00663347280327	-0.95782426046556	-0.17230285209050
H	5.28975585011547	-0.64625714418624	-0.95828890772861

Table S45. Transition-state geometry of $\mathscr{O}_{1w}$ (TS_1).

Atom	x /Å	y /Å	z /Å
C	-0.75879425867926	1.09093196829392	1.67460749483135
C	-1.58559675141041	-0.22669491524959	1.65873173412604
C	-1.53779705409298	-0.70485981707781	0.17684400095533
C	-0.10107020854278	-1.19055873355793	0.00486728437681
C	0.71530758308033	0.07626877900436	0.05101986902172
C	-0.26740785474145	1.20237840290095	0.21043950601736
C	-2.58748176656096	-1.72504524450477	-0.20292206202928
C	-1.47303292615532	0.64742226898823	-0.60071713124937
C	-1.15470314099794	0.47540959685166	-2.08905704903457
C	-2.73655331761578	1.49789111249500	-0.47255871269848
O	0.29138136033371	-2.32998544301161	-0.12560813582336
N	1.99408201922394	0.05094202709048	-0.00842544636012
O	2.54724028066074	1.32301439453887	0.07641804650231
H	-1.37414779753715	1.95180670815740	1.94388860218389
H	0.07931018466399	1.05471800156014	2.37387632610427
H	-1.17859032153307	-0.98742659535705	2.33107775331802
H	-2.62434877149520	-0.05948182584195	1.95515260277530
H	0.11005465948159	2.18147827789122	-0.08511239950821
H	-2.47734932213705	-2.62552215287810	0.40678446367239
H	-3.59349098781872	-1.32624151632938	-0.04670798267189
H	-2.48907841339303	-2.02583299752681	-1.24904107829866
H	-0.26326571043375	-0.13269681997097	-2.26761415372814
H	-1.99184021945781	0.00109636073838	-2.60914443697277
H	-0.98580623754056	1.45247915182622	-2.55224035529864
H	-3.07048516174881	1.62851353393724	0.55727238232985
H	-2.55867803694408	2.49204527922779	-0.89517115005612
H	-3.55762694963943	1.04333354405229	-1.03441719210519
H	3.49956366711873	1.12762176718470	0.00807437256807
O	4.75264983939997	-0.37158207512929	-0.19093460280178
H	3.97575402015438	-0.94952810581693	-0.19935979275615
H	5.52261761705820	-0.94102836378666	-0.27612715128995

Table S46. Equilibrium geometry of $\mathscr{C}_{1w}$.

Atom	x /Å	y /Å	z /Å
C	-0.28892851789767	0.89419277867113	1.74550751351903
C	-1.50082469644067	-0.07020159521538	1.60498704111304
C	-1.57084124594467	-0.39815967859012	0.08067106137270
C	-0.37019861791996	-1.29748339857597	-0.12923042029898
C	0.81754825961512	-0.39679559323566	0.01832867575588
C	0.25997428330005	0.97143434179611	0.30117531215475
C	-2.88424681749511	-0.97767910634078	-0.39561807796753
C	-1.03695244969241	0.92637142184485	-0.55988918392108
C	-0.75210665918825	0.80050978021656	-2.05981090660916
C	-1.95606801217213	2.12800018937480	-0.34408706320218
O	-0.34208912043473	-2.49922376993993	-0.35639282051177
N	2.06824258253470	-0.69201625059514	-0.06738324337617
O	2.37565381100141	-2.00965649063450	-0.32756363337304
H	-0.59642777075479	1.88086886653567	2.09642303215756
H	0.46951235141886	0.52299692401981	2.43767984012066
H	-1.39065038395849	-0.98115412267696	2.20051152941564
H	-2.43781005183316	0.40094425485289	1.91210243375595
H	0.95201684929458	1.79134277718478	0.10741656969964
H	-2.85688767927822	-1.20017681253455	-1.46533779921630
H	-3.10074397608327	-1.91207818752985	0.12875346624872
H	-3.70495625387480	-0.28143594493613	-0.20519048715551
H	-0.11282722814415	-0.05194948888569	-2.30747890508837
H	-1.68478664024834	0.69075343304417	-2.62041939259139
H	-0.24867498199034	1.70188342813415	-2.42168354425596
H	-2.25739346481970	2.26557450739261	0.69466445037053
H	-1.44864903503777	3.04187416115330	-0.66827913285372
H	-2.86416353498360	2.02377092225009	-0.94484204987551
H	1.53017011710988	-2.50535633429706	-0.39438021319312
O	3.47539219764595	1.88104950499187	0.38981306840677
H	4.43417690290776	1.92390875799548	0.46208599640617
H	3.27860786386396	0.94654460822944	0.21218398329274

3.2.2 Transition frequencies

Table S47. Transition frequencies for $\mathcal{O}_{1w}$. J is the rotational angular momentum quantum number, K_a and K_c are the projections of J onto the principal axes at the prolate and oblate symmetric top limits, and F is the total angular momentum quantum number, which includes the nuclear spin, I(^{14}N) = 1.

Observed Frequency /MHz	Calculated Frequency /MHz	Difference / MHz	J	K_a	K_c	F	J'	K_a'	K_c'	F'
2359.8050	2359.8085	-0.0035	2	1	2	1	1	0	1	0
2360.5427	2360.5442	-0.0015	2	1	2	3	1	0	1	2
2360.7099	2360.7238	-0.0139	2	1	2	2	1	0	1	1
2545.7185	2545.7211	-0.0026	3	1	3	3	2	1	2	2
2545.9387	2545.9287	0.0100	3	1	3	4	2	1	2	3
2601.4659	2601.4609	0.0050	3	0	3	2	2	0	2	1
2601.6085	2601.5976	0.0109	3	0	3	4	2	0	2	3
2609.1990	2609.2046	-0.0056	3	2	2	4	2	2	1	3
2609.6008	2609.6058	-0.0050	3	2	2	2	2	2	1	1
2615.9635	2615.9710	-0.0075	3	2	1	3	2	2	0	2
2616.6722	2616.6815	-0.0093	3	2	1	4	2	2	0	3
2617.0766	2617.0802	-0.0036	3	2	1	2	2	2	0	1
2669.7471	2669.7407	0.0064	3	1	2	3	2	1	1	2
2669.9305	2669.9387	-0.0082	3	1	2	4	2	1	1	3
3168.7713	3168.7743	-0.0030	3	1	3	2	2	0	2	1
3168.9094	3168.9338	-0.0244	3	1	3	4	2	0	2	3
3392.3547	3392.3554	-0.0007	4	1	4	4	3	1	3	3
3392.4572	3392.4512	0.0060	4	1	4	5	3	1	3	4
3460.2235	3460.2147	0.0088	4	0	4	5	3	0	3	4
3460.2276	3460.2147	0.0129	4	0	4	5	3	0	3	4
3477.0513	3477.0377	0.0136	4	2	3	4	3	2	2	3
3477.3413	3477.3457	-0.0044	4	2	3	5	3	2	2	4
3477.4423	3477.4248	0.0175	4	2	3	3	3	2	2	2
3495.5832	3495.5822	0.0010	4	2	2	4	3	2	1	3
3495.8692	3495.8753	-0.0061	4	2	2	5	3	2	1	4
3495.9711	3495.9522	0.0189	4	2	2	3	3	2	1	2
3557.4722	3557.4665	0.0057	4	1	3	4	3	1	2	3
3557.5614	3557.5566	0.0048	4	1	3	5	3	1	2	4
3768.2505	3768.2550	-0.0045	2	2	1	3	1	1	0	2
3811.4518	3811.4461	0.0057	2	2	0	3	1	1	1	2
3811.9630	3811.9625	0.0005	2	2	0	2	1	1	1	1
3959.7975	3959.7874	0.0101	4	1	4	5	3	0	3	4
4237.3247	4237.3125	0.0122	5	1	5	5	4	1	4	4
4237.3684	4237.3677	0.0007	5	1	5	6	4	1	4	5
4344.1199	4344.1183	0.0016	5	2	4	5	4	2	3	4
4344.2841	4344.2813	0.0028	5	2	4	6	4	2	3	5
4354.0642	4354.0512	0.0130	5	3	3	5	4	3	2	4
4354.3905	4354.3940	-0.0035	5	3	3	6	4	3	2	5
4354.4669	4354.4784	-0.0115	5	3	3	4	4	3	2	3
4355.1540	4355.1660	-0.0120	5	3	2	5	4	3	1	4
4380.5333	4380.5335	-0.0002	5	2	3	5	4	2	2	4
4380.6824	4380.6785	0.0039	5	2	3	6	4	2	2	5
4442.9898	4442.9860	0.0038	5	1	4	5	4	1	3	4
4443.0433	4443.0380	0.0053	5	1	4	6	4	1	3	5
4572.7084	4572.7164	-0.0080	7	1	6	8	6	2	5	7
4572.7084	4572.7112	-0.0028	7	1	6	6	6	2	5	5
4596.4825	4596.4823	0.0002	3	2	2	2	2	1	1	1

4596.6087	4596.6091	-0.0004	3	2	2	4	2	1	1	3
4596.8356	4596.8368	-0.0012	3	2	2	3	2	1	1	2
4729.6981	4729.6938	0.0043	3	2	1	2	2	1	2	1
4729.9723	4729.9747	-0.0024	3	2	1	4	2	1	2	3
4730.4945	4730.4869	0.0076	3	2	1	3	2	1	2	2
4731.3833	4731.3753	0.0080	6	0	6	7	5	1	5	6
4736.8823	4736.8863	-0.0040	5	1	5	5	4	0	4	4
4736.9486	4736.9404	0.0082	5	1	5	6	4	0	4	5
5080.3973	5080.4081	-0.0108	6	1	6	6	5	1	5	5
5080.4615	5080.4448	0.0167	6	1	6	7	5	1	5	6
5156.2743	5156.2747	-0.0004	6	0	6	6	5	0	5	5
5209.5745	5209.5793	-0.0048	6	2	5	6	5	2	4	5
5209.6835	5209.6780	0.0055	6	2	5	7	5	2	4	6
5226.8162	5226.8129	0.0033	6	3	4	6	5	3	3	5
5226.9965	5227.0134	-0.0169	6	3	4	7	5	3	3	6
5229.7643	5229.7700	-0.0057	6	3	3	6	5	3	2	5
5229.9465	5229.9685	-0.0220	6	3	3	7	5	3	2	6
5271.4201	5271.4228	-0.0027	6	2	4	7	5	2	3	6
5325.6428	5325.6323	0.0105	6	1	5	7	5	1	4	6
5404.0005	5404.0162	-0.0157	4	2	3	5	3	1	2	4
5404.1399	5404.1339	0.0060	4	2	3	4	3	1	2	3
5505.3685	5505.3731	-0.0046	6	1	6	7	5	0	5	6
5577.9995	5578.0067	-0.0072	8	1	7	7	7	2	6	6
5644.3121	5644.3049	0.0072	7	0	7	8	6	1	6	7
5652.0574	5652.0400	0.0174	4	2	3	5	3	1	3	4
5679.7437	5679.7463	-0.0026	4	2	2	3	3	1	3	2
5679.9271	5679.9212	0.0059	4	2	2	5	3	1	3	4
5680.3554	5680.3480	0.0074	4	2	2	4	3	1	3	3
5921.5445	5921.5579	-0.0134	7	1	7	8	6	1	6	7
5993.3543	5993.3573	-0.0030	7	0	7	6	6	0	6	5
5993.3600	5993.3745	-0.0145	7	0	7	8	6	0	6	7
6024.3293	6024.3249	0.0044	3	3	1	4	2	2	0	3
6026.2678	6026.2530	0.0148	3	3	0	4	2	2	1	3
6073.2121	6073.2314	-0.0193	7	2	6	8	6	2	5	7
6094.1257	6094.1247	0.0010	7	5	2	7	6	5	1	6
6094.1257	6094.1232	0.0025	7	5	3	7	6	5	2	6
6100.0728	6100.0738	-0.0010	7	3	5	7	6	3	4	6
6100.2091	6100.2012	0.0079	7	3	5	8	6	3	4	7
6106.6602	6106.6697	-0.0095	7	3	4	7	6	3	3	6
6106.7912	6106.7940	-0.0028	7	3	4	8	6	3	3	7
6167.4833	6167.4732	0.0101	7	2	5	8	6	2	4	7
6167.4834	6167.4732	0.0102	7	2	5	8	6	2	4	7
6204.4610	6204.4650	-0.0040	7	1	6	8	6	1	5	7
6270.6355	6270.6275	0.0080	7	1	7	8	6	0	6	7
6502.6046	6502.6106	-0.0060	10	0	10	10	9	1	8	9
6547.2368	6547.2341	0.0027	8	0	8	9	7	1	7	8
6668.0264	6668.0289	-0.0025	5	2	3	4	4	1	4	3
6668.1615	6668.1485	0.0130	5	2	3	6	4	1	4	5
6668.5061	6668.5262	-0.0201	5	2	3	5	4	1	4	4
6760.6814	6760.6879	-0.0065	8	1	8	9	7	1	7	8
6824.4775	6824.4871	-0.0096	8	0	8	9	7	0	7	8
6890.1163	6890.1207	-0.0044	4	3	2	5	3	2	1	4
6890.3804	6890.3760	0.0044	4	3	2	4	3	2	1	3
6899.8358	6899.8447	-0.0089	4	3	1	5	3	2	2	4
6900.1337	6900.1188	0.0149	4	3	1	4	3	2	2	3

6934.6728	6934.6606	0.0122	8	2	7	9	7	2	6	8
6957.3794	6957.3809	-0.0015	6	2	5	7	5	1	4	6
6966.3853	6966.3950	-0.0097	8	5	3	8	7	5	2	7
6966.3853	6966.3891	-0.0038	8	5	4	8	7	5	3	7
6966.6128	6966.6287	-0.0159	8	5	3	9	7	5	2	8
6966.6128	6966.6229	-0.0101	8	5	4	9	7	5	3	8
6970.2998	6970.2991	0.0007	8	4	5	8	7	4	4	7
6970.4677	6970.4494	0.0183	8	4	5	9	7	4	4	8
6970.4677	6970.4664	0.0013	8	4	5	7	7	4	4	6
6970.7207	6970.7096	0.0111	8	4	4	8	7	4	3	7
6970.8702	6970.8597	0.0105	8	4	4	9	7	4	3	8
6973.7238	6973.7319	-0.0081	8	3	6	9	7	3	5	8
6986.7288	6986.7425	-0.0137	8	3	5	9	7	3	4	8
7037.9540	7037.9409	0.0131	8	1	8	9	7	0	7	8
7067.1935	7067.1997	-0.0062	8	2	6	9	7	2	5	8
7078.5238	7078.5309	-0.0071	8	1	7	9	7	1	6	8
7438.0511	7438.0480	0.0031	9	0	9	10	8	1	8	9
7597.9092	7597.9107	-0.0015	9	1	9	10	8	1	8	9
7604.3215	7604.3257	-0.0042	10	1	9	11	9	2	8	10
7651.5009	7651.5018	-0.0009	9	0	9	10	8	0	8	9
7702.1973	7702.2035	-0.0062	6	2	4	7	5	1	5	6
7702.5599	7702.5579	0.0020	6	2	4	6	5	1	5	5
7704.9842	7704.9800	0.0042	7	2	6	8	6	1	5	7
7748.6220	7748.6395	-0.0175	5	3	3	6	4	2	2	5
7748.8417	7748.8449	-0.0032	5	3	3	5	4	2	2	4
7777.9846	7778.0068	-0.0222	5	3	2	6	4	2	3	5
7778.2487	7778.2471	0.0016	5	3	2	5	4	2	3	4
7793.7239	7793.7138	0.0101	9	2	8	10	8	2	7	9
7839.2509	7839.2634	-0.0125	9	5	4	9	8	5	3	8
7839.2509	7839.2444	0.0065	9	5	5	9	8	5	4	8
7839.4330	7839.4284	0.0046	9	5	4	10	8	5	3	9
7844.6059	7844.5943	0.0116	9	4	6	9	8	4	5	8
7844.7099	7844.7002	0.0097	9	4	6	10	8	4	5	9
7844.7099	7844.7084	0.0015	9	4	6	8	8	4	5	7
7845.5868	7845.5737	0.0131	9	4	5	9	8	4	4	8
7845.6878	7845.6791	0.0087	9	4	5	10	8	4	4	9
7845.6878	7845.6873	0.0005	9	4	5	8	8	4	4	7
7847.2837	7847.2900	-0.0063	9	3	7	10	8	3	6	9
7870.6774	7870.6712	0.0062	9	3	6	10	8	3	5	9
7870.6774	7870.6706	0.0068	9	3	6	8	8	3	5	7
7946.7238	7946.7280	-0.0042	9	1	8	10	8	1	7	9
7968.3315	7968.3371	-0.0056	9	2	7	10	8	2	6	9

Table S48. Transition frequencies for $\mathscr{O}_{1w}$: O(29). J is the rotational angular momentum quantum number, K_a and K_c are the projections of J onto the principal axes at the prolate and oblate symmetric top limits, and F is the total angular momentum quantum number, which includes the nuclear spin, I(^{14}N) = 1.

Observed Frequency /MHz	Calculated Frequency /MHz	Difference / MHz	J	K_a	K_c	F	J'	K'_a	K'_c	F'
2567.1802	2567.1783	0.0019	3	1	2	3	2	1	1	2
2567.3820	2567.3684	0.0136	3	1	2	4	2	1	1	3
2567.3820	2567.3850	-0.0030	3	1	2	2	2	1	1	1
3267.8472	3267.8434	0.0038	4	1	4	4	3	1	3	3
3267.9528	3267.9339	0.0189	4	1	4	5	3	1	3	4
3346.2186	3346.2388	-0.0202	4	2	3	4	3	2	2	3
3346.5394	3346.5338	0.0056	4	2	3	5	3	2	2	4
3361.8754	3361.8802	-0.0048	4	2	2	4	3	2	1	3
3421.0971	3421.0891	0.0080	4	1	3	4	3	1	2	3
3421.1896	3421.1750	0.0146	4	1	3	5	3	1	2	4
3627.2933	3627.3113	-0.0180	5	0	5	4	4	1	4	3
3751.0594	3751.0599	-0.0005	2	2	1	3	1	1	0	2
3790.9962	3790.9780	0.0182	2	2	0	3	1	1	1	2
3858.5199	3858.5366	-0.0167	4	1	4	5	3	0	3	4
4082.1855	4082.1994	-0.0139	5	1	5	6	4	1	4	5
4082.1855	4082.1704	0.0151	5	1	5	4	4	1	4	3
4180.9824	4180.9851	-0.0027	5	2	4	5	4	2	3	4
4181.1537	4181.1407	0.0130	5	2	4	6	4	2	3	5
4181.1537	4181.1566	-0.0029	5	2	4	4	4	2	3	3
4211.7940	4211.7955	-0.0015	5	2	3	5	4	2	2	4
4211.9360	4211.9383	-0.0023	5	2	3	6	4	2	2	5
4211.9360	4211.9528	-0.0168	5	2	3	4	4	2	2	3
4273.2169	4273.2071	0.0098	5	1	4	6	4	1	3	5
4514.1350	4514.1302	0.0048	6	0	6	7	5	1	5	6
4549.5147	4549.5040	0.0107	3	2	2	2	2	1	1	1
4549.6399	4549.6384	0.0015	3	2	2	4	2	1	1	3
4549.8814	4549.8798	0.0016	3	2	2	3	2	1	1	2
4608.6303	4608.6344	-0.0041	5	1	5	5	4	0	4	4
4608.6303	4608.6337	-0.0034	5	1	5	4	4	0	4	3
4894.8543	4894.8475	0.0068	6	1	6	6	5	1	5	5
4894.8543	4894.8593	-0.0050	6	1	6	5	5	1	5	4
4968.9670	4968.9647	0.0023	6	0	6	6	5	0	5	5
4968.9670	4968.9641	0.0029	6	0	6	5	5	0	5	4
5014.3455	5014.3597	-0.0142	6	2	5	6	5	2	4	5
5014.4634	5014.4534	0.0100	6	2	5	7	5	2	4	6
5014.4634	5014.4534	0.0100	6	2	5	5	5	2	4	4
5066.9528	5066.9646	-0.0118	6	2	4	7	5	2	3	6
5066.9528	5066.9635	-0.0107	6	2	4	5	5	2	3	4
5122.8033	5122.8207	-0.0174	6	1	5	6	5	1	4	5
5228.0942	5228.0838	0.0104	8	1	7	9	7	2	6	8
5228.0942	5228.0774	0.0168	8	1	7	7	7	2	6	6
5328.7969	5328.8038	-0.0069	4	2	3	5	3	1	2	4
5328.9381	5328.9403	-0.0022	4	2	3	4	3	1	2	3
5349.7557	5349.7386	0.0171	6	1	6	7	5	0	5	6
5396.8771	5396.8633	0.0138	7	0	7	8	6	1	6	7
5705.8222	5705.8271	-0.0049	7	1	7	7	6	1	6	6
5705.8222	5705.8346	-0.0124	7	1	7	6	6	1	6	5
5777.6035	5777.6143	-0.0108	7	0	7	8	6	0	6	7
5777.6035	5777.5925	0.0110	7	0	7	7	6	0	6	6

5777.6035	5777.5976	0.0059	7	0	7	6	6	0	6	5
5846.2010	5846.2120	-0.0110	7	2	6	8	6	2	5	7
5846.2010	5846.2076	-0.0066	7	2	6	6	6	2	5	5
5869.1644	5869.1651	-0.0007	7	3	5	8	6	3	4	7
5869.1644	5869.1764	-0.0120	7	3	5	6	6	3	4	5
5874.2127	5874.2145	-0.0018	7	3	4	8	6	3	3	7
5874.2127	5874.2255	-0.0128	7	3	4	6	6	3	3	5
5926.9461	5926.9390	0.0071	7	2	5	8	6	2	4	7
5926.9461	5926.9335	0.0126	7	2	5	6	6	2	4	5
5969.4023	5969.4061	-0.0038	7	1	6	8	6	1	5	7
5969.4023	5969.3949	0.0074	7	1	6	6	6	1	5	5
6086.6184	6086.6024	0.0160	7	1	7	8	6	0	6	7
6201.7907	6201.7957	-0.0050	9	1	8	10	8	2	7	9
6201.7907	6201.7878	0.0029	9	1	8	8	8	2	7	7
6515.0592	6515.0674	-0.0082	8	1	8	9	7	1	7	8
6515.0592	6515.0487	0.0105	8	1	8	8	7	1	7	7
6515.0592	6515.0542	0.0050	8	1	8	7	7	1	7	6
6580.5145	6580.5226	-0.0081	8	0	8	9	7	0	7	8
6580.5145	6580.5022	0.0123	8	0	8	8	7	0	7	7
6580.5145	6580.5100	0.0045	8	0	8	7	7	0	7	6
6676.1808	6676.1747	0.0061	8	2	7	9	7	2	6	8
6676.1808	6676.1691	0.0117	8	2	7	7	7	2	6	6
6709.5248	6709.5417	-0.0169	8	3	6	9	7	3	5	8
6709.5248	6709.5451	-0.0203	8	3	6	7	7	3	5	6
6790.7137	6790.6981	0.0156	8	2	6	8	7	2	5	7
6790.7137	6790.7205	-0.0068	8	2	6	7	7	2	5	6
6790.7143	6790.7268	-0.0125	8	2	6	9	7	2	5	8
6812.0437	6812.0523	-0.0086	8	1	7	9	7	1	6	8
6812.0437	6812.0320	0.0117	8	1	7	8	7	1	6	7
6812.0437	6812.0434	0.0003	8	1	7	7	7	1	6	6
6824.0643	6824.0555	0.0088	8	1	8	9	7	0	7	8
6830.0161	6830.0157	0.0004	6	2	5	7	5	1	4	6
7135.6261	7135.6248	0.0013	9	0	9	10	8	1	8	9
7135.6261	7135.6269	-0.0008	9	0	9	9	8	1	8	8
7135.6261	7135.6123	0.0138	9	0	9	8	8	1	8	7
7322.5531	7322.5665	-0.0134	9	1	9	10	8	1	8	9
7322.5531	7322.5514	0.0017	9	1	9	9	8	1	8	8
7322.5531	7322.5559	-0.0028	9	1	9	8	8	1	8	7
7379.1443	7379.1578	-0.0135	9	0	9	10	8	0	8	9
7379.1443	7379.1392	0.0051	9	0	9	9	8	0	8	8
7379.1443	7379.1478	-0.0035	9	0	9	8	8	0	8	7
7504.1183	7504.1204	-0.0021	9	2	8	10	8	2	7	9
7504.1183	7504.1149	0.0034	9	2	8	8	8	2	7	7
7568.0690	7568.0741	-0.0051	9	3	6	10	8	3	5	9
7568.0690	7568.0737	-0.0047	9	3	6	8	8	3	5	7
7649.8772	7649.8866	-0.0094	9	1	8	10	8	1	7	9
7649.8772	7649.8683	0.0089	9	1	8	9	8	1	7	8
7649.8772	7649.8795	-0.0023	9	1	8	8	8	1	7	7
7656.5423	7656.5549	-0.0126	9	2	7	10	8	2	6	9
7656.5423	7656.5361	0.0062	9	2	7	9	8	2	6	8
7656.5423	7656.5489	-0.0066	9	2	7	8	8	2	6	7
7988.2208	7988.2185	0.0023	10	0	10	11	9	1	9	10
7988.2208	7988.2192	0.0016	10	0	10	10	9	1	9	9
7988.2208	7988.2085	0.0123	10	0	10	9	9	1	9	8

Table S49. Transition frequencies for $\mathscr{C}_{1w}$. J is the rotational angular momentum quantum number, K_a and K_c are the projections of J onto the principal axes at the prolate and oblate symmetric top limits, and F is the total angular momentum quantum number, which includes the nuclear spin, I(^{14}N) = 1.

Observed Frequency /MHz	Calculated Frequency /MHz	Difference / MHz	J	K_a	K_c	F	J'	K_a'	K_c'	F'
2003.8006	2003.7969	0.0037	2	1	2	3	1	1	1	2
2097.4488	2097.4506	-0.0018	2	0	2	3	1	0	1	2
2097.4488	2097.4520	-0.0032	2	0	2	2	1	0	1	1
2260.6767	2260.6745	0.0022	2	1	1	2	1	1	0	1
2261.2506	2261.2504	0.0002	2	1	1	2	1	1	0	2
2262.0154	2262.0187	-0.0033	2	1	1	3	1	1	0	2
2263.3129	2263.3117	0.0012	2	1	1	1	1	1	0	0
2284.5707	2284.5700	0.0007	2	1	2	3	1	0	1	2
2285.2516	2285.2462	0.0054	2	1	2	2	1	0	1	1
2884.6396	2884.6321	0.0075	3	0	3	3	2	1	2	2
2885.2585	2885.2568	0.0017	3	0	3	4	2	1	2	3
2984.7492	2984.7343	0.0149	3	1	3	3	2	1	2	3
2985.3556	2985.3518	0.0038	3	1	3	3	2	1	2	2
2985.7120	2985.7091	0.0029	3	1	3	4	2	1	2	3
2985.7120	2985.7062	0.0058	3	1	3	2	2	1	2	1
3071.1357	3071.1340	0.0017	3	0	3	3	2	0	2	3
3072.0962	3072.0928	0.0034	3	0	3	2	2	0	2	1
3072.3948	3072.3762	0.0186	3	0	3	4	2	0	2	3
3074.1095	3074.1041	0.0054	3	0	3	2	2	0	2	2
3103.5288	3103.5203	0.0085	2	2	1	1	1	1	0	1
3104.8624	3104.8660	-0.0036	2	2	1	3	1	1	0	2
3105.6825	3105.6763	0.0062	2	2	1	2	1	1	0	1
3106.2524	3106.2522	0.0002	2	2	1	2	1	1	0	2
3172.8317	3172.8285	0.0032	3	1	3	4	2	0	2	3
3197.8815	3197.8853	-0.0038	3	2	2	3	2	2	1	2
3199.2688	3199.2713	-0.0025	3	2	2	4	2	2	1	3
3200.0337	3200.0422	-0.0085	3	2	2	2	2	2	1	1
3269.2839	3269.2837	0.0002	2	2	0	3	1	1	1	2
3269.6308	3269.6416	-0.0108	2	2	0	1	1	1	1	0
3269.8665	3269.8591	0.0074	2	2	0	2	1	1	1	1
3324.4173	3324.4222	-0.0049	3	2	1	3	2	2	0	2
3324.6756	3324.6868	-0.0112	3	2	1	2	2	2	0	2
3325.7125	3325.7144	-0.0019	3	2	1	3	2	2	0	3
3325.9062	3325.9096	-0.0034	3	2	1	4	2	2	0	3
3326.6914	3326.6966	-0.0052	3	2	1	2	2	2	0	1
3365.6035	3365.6127	-0.0092	3	1	2	3	2	1	1	3
3366.3797	3366.3811	-0.0014	3	1	2	3	2	1	1	2
3366.7385	3366.7271	0.0114	3	1	2	4	2	1	1	3
3367.8887	3367.8863	0.0024	3	1	2	2	2	1	1	2
3550.0678	3550.0569	0.0109	2	2	0	3	1	0	1	2
3552.6585	3552.6427	0.0158	2	2	0	2	1	0	1	1
3755.5696	3755.5756	-0.0060	4	1	3	4	3	2	2	3
3757.0972	3757.0967	0.0005	4	1	3	3	3	2	2	2
3905.9417	3905.9364	0.0053	4	0	4	4	3	1	3	3
3906.1981	3906.1764	0.0217	4	0	4	5	3	1	3	4
3950.3981	3950.4050	-0.0069	4	1	4	4	3	1	3	4

3951.3828	3951.3798	0.0030	4	1	4	4	3	1	3	3
3951.5110	3951.5210	-0.0100	4	1	4	5	3	1	3	4
3952.7904	3952.7836	0.0068	4	1	4	3	3	1	3	3
4005.4229	4005.4139	0.0090	4	0	4	4	3	0	3	4
4006.5171	4006.5061	0.0110	4	0	4	3	3	0	3	2
4006.6222	4006.6287	-0.0065	4	0	4	5	3	0	3	4
4008.1838	4008.1840	-0.0002	4	0	4	3	3	0	3	3
4041.6965	4041.6912	0.0053	3	2	2	2	2	1	1	1
4042.1244	4042.1185	0.0059	3	2	2	4	2	1	1	3
4042.8932	4042.8871	0.0061	3	2	2	3	2	1	1	2
4051.8396	4051.8254	0.0142	4	1	4	3	3	0	3	2
4051.9919	4051.9733	0.0186	4	1	4	5	3	0	3	4
4052.1076	4052.0995	0.0081	4	1	4	4	3	0	3	3
4236.9173	4236.9201	-0.0028	4	2	3	4	3	2	2	3
4237.4834	4237.4839	-0.0005	4	2	3	5	3	2	2	4
4237.6462	4237.6285	0.0177	4	2	3	3	3	2	2	2
4319.8826	4319.8839	-0.0013	4	3	2	4	3	3	1	3
4321.1937	4321.1949	-0.0012	4	3	2	5	3	3	1	4
4321.6995	4321.6971	0.0024	4	3	2	3	3	3	1	2
4355.5896	4355.5940	-0.0044	4	3	1	4	3	3	0	3
4356.9492	4356.9538	-0.0046	4	3	1	5	3	3	0	4
4357.4660	4357.4670	-0.0010	4	3	1	3	3	3	0	2
4430.9807	4430.9673	0.0134	4	1	3	4	3	1	2	4
4432.0869	4432.0816	0.0053	4	1	3	4	3	1	2	3
4432.1777	4432.1775	0.0002	4	1	3	5	3	1	2	4
4433.5974	4433.6036	-0.0062	4	1	3	3	3	1	2	3
4494.4264	4494.4181	0.0083	4	2	2	4	3	2	1	4
4494.6120	4494.6133	-0.0013	4	2	2	4	3	2	1	3
4495.2342	4495.2349	-0.0007	4	2	2	5	3	2	1	4
4495.3742	4495.3763	-0.0021	4	2	2	3	3	2	1	2
4495.6366	4495.6409	-0.0043	4	2	2	3	3	2	1	3
4591.1153	4591.1207	-0.0054	3	2	1	2	2	1	2	1
4591.3890	4591.3965	-0.0075	3	2	1	4	2	1	2	3
4591.8106	4591.8187	-0.0081	3	2	1	3	2	1	2	2
4777.8483	4777.8663	-0.0180	3	2	1	2	2	0	2	1
4778.5168	4778.5159	0.0009	3	2	1	4	2	0	2	3
4779.6132	4779.6129	0.0003	3	2	1	3	2	0	2	2
4886.0044	4885.9907	0.0137	5	0	5	5	4	1	4	4
4903.1700	4903.1787	-0.0087	5	1	5	5	4	1	4	5
4904.3430	4904.3629	-0.0199	5	1	5	6	4	1	4	5
4905.7292	4905.7207	0.0085	5	1	5	4	4	1	4	4
4911.5542	4911.5532	0.0010	3	3	1	4	2	2	0	3
4911.6503	4911.6508	-0.0005	3	3	1	2	2	2	0	1
4912.0331	4912.0330	0.0001	3	3	1	3	2	2	0	2
4912.8551	4912.8753	-0.0202	4	2	3	5	3	1	2	4
4913.4331	4913.4262	0.0069	4	2	3	4	3	1	2	3
4930.2225	4930.2194	0.0031	5	0	5	5	4	0	4	5
4931.4318	4931.4373	-0.0055	5	0	5	6	4	0	4	5
4931.4318	4931.4342	-0.0024	5	0	5	5	4	0	4	4
4932.8950	4932.9007	-0.0057	5	0	5	4	4	0	4	4
4953.1505	4953.1425	0.0080	3	3	0	4	2	2	1	3
4953.3027	4953.2983	0.0044	3	3	0	2	2	2	1	1
4953.5190	4953.5115	0.0075	3	3	0	3	2	2	1	2
4954.8965	4954.8977	-0.0012	3	3	0	3	2	2	1	3
4957.7993	4957.8040	-0.0047	5	1	4	5	4	2	3	4

4958.4452	4958.4411	0.0041	5	1	4	6	4	2	3	5
5252.9686	5252.9674	0.0012	5	2	4	5	4	2	3	5
5253.5231	5253.5310	-0.0079	5	2	4	5	4	2	3	4
5253.7988	5253.8024	-0.0036	5	2	4	6	4	2	3	5
5254.5309	5254.5366	-0.0057	5	2	4	4	4	2	3	4
5401.4167	5401.4225	-0.0058	5	3	3	5	4	3	2	4
5402.0855	5402.0903	-0.0048	5	3	3	6	4	3	2	5
5402.2491	5402.2508	-0.0017	5	3	3	4	4	3	2	3
5408.5301	5408.5425	-0.0124	5	4	2	5	4	4	1	4
5409.7369	5409.7414	-0.0045	5	4	2	6	4	4	1	5
5410.0793	5410.0947	-0.0154	5	4	2	4	4	4	1	3
5415.6845	5415.6928	-0.0083	5	4	1	5	4	4	0	4
5416.8980	5416.9025	-0.0045	5	4	1	6	4	4	0	5
5417.2503	5417.2579	-0.0076	5	4	1	4	4	4	0	3
5437.9512	5437.9384	0.0128	5	1	4	5	4	1	3	5
5439.1293	5439.1390	-0.0097	5	1	4	6	4	1	3	5
5439.1293	5439.1485	-0.0192	5	1	4	5	4	1	3	4
5440.5945	5440.5943	0.0002	5	1	4	4	4	1	3	4
5511.9518	5511.9497	0.0021	5	3	2	5	4	3	1	4
5512.6999	5512.6974	0.0025	5	3	2	6	4	3	1	5
5512.8737	5512.8707	0.0030	5	3	2	4	4	3	1	3
5586.3307	5586.3198	0.0109	6	2	4	6	5	3	3	5
5587.3345	5587.3224	0.0121	6	2	4	7	5	3	3	6
5645.7055	5645.7054	0.0001	5	2	3	5	4	2	2	5
5646.5252	5646.5222	0.0030	5	2	3	5	4	2	2	4
5646.8096	5646.8025	0.0071	5	2	3	6	4	2	2	5
5646.8096	5646.8157	-0.0061	5	2	3	4	4	2	2	3
5647.8630	5647.8433	0.0197	5	2	3	4	4	2	2	4
5734.5080	5734.5002	0.0078	5	2	4	6	4	1	3	5
5734.8741	5734.8755	-0.0014	5	2	4	5	4	1	3	4
5842.0528	5842.0588	-0.0060	6	0	6	6	5	1	5	5
5847.7442	5847.7402	0.0040	6	1	6	6	5	1	5	6
5848.9129	5848.9243	-0.0114	6	1	6	6	5	1	5	5
5848.9129	5848.9287	-0.0158	6	1	6	5	5	1	5	4
5859.1525	5859.1449	0.0076	6	0	6	6	5	0	5	6
5860.3932	5860.3791	0.0141	6	0	6	7	5	0	5	6
5867.2494	5867.2337	0.0157	6	1	6	7	5	0	5	6
5867.2494	5867.2282	0.0212	6	1	6	6	5	0	5	5
5906.6495	5906.6513	-0.0018	4	3	2	3	3	2	1	2
5906.8438	5906.8385	0.0053	4	3	2	5	3	2	1	4
5907.4968	5907.4947	0.0021	4	3	2	4	3	2	1	3
6090.7608	6090.7697	-0.0089	6	1	5	6	5	2	4	5
6091.0887	6091.0950	-0.0063	6	1	5	7	5	2	4	6
6100.9207	6100.9223	-0.0016	4	2	2	5	3	1	3	4
6101.0730	6101.0803	-0.0073	4	2	2	4	3	1	3	3
6110.7200	6110.7231	-0.0031	4	3	1	3	3	2	2	2
6110.8306	6110.8250	0.0056	4	3	1	5	3	2	2	4
6111.2276	6111.2202	0.0074	4	3	1	4	3	2	2	3
6177.0542	6177.0400	0.0142	3	3	1	4	2	1	2	3
6201.1412	6201.1497	-0.0085	4	2	2	3	3	0	3	2
6201.3667	6201.3746	-0.0079	4	2	2	5	3	0	3	4
6201.7905	6201.7999	-0.0094	4	2	2	4	3	0	3	3
6245.8908	6245.9021	-0.0113	6	2	5	6	5	2	4	6
6246.7253	6246.7370	-0.0117	6	2	5	6	5	2	4	5
6246.8670	6246.8787	-0.0117	6	2	5	7	5	2	4	6

6385.2886	6385.2960	-0.0074	6	1	5	6	5	1	4	6
6387.8506	6387.8533	-0.0027	6	1	5	5	5	1	4	5
6468.2132	6468.2258	-0.0126	6	3	4	6	5	3	3	6
6468.4230	6468.4326	-0.0096	6	3	4	6	5	3	3	5
6468.8128	6468.8069	0.0059	6	3	4	7	5	3	3	6
6469.1060	6469.1123	-0.0063	6	3	4	5	5	3	3	5
6488.9544	6488.9579	-0.0035	6	5	2	7	5	5	1	6
6490.1477	6490.1555	-0.0078	6	5	1	7	5	5	0	6
6508.2549	6508.2573	-0.0024	6	4	3	6	5	4	2	5
6508.9567	6508.9607	-0.0040	6	4	3	7	5	4	2	6
6509.1053	6509.1085	-0.0032	6	4	3	5	5	4	2	4
6538.3789	6538.3816	-0.0027	6	4	2	6	5	4	1	5
6539.1119	6539.1146	-0.0027	6	4	2	7	5	4	1	6
6539.2664	6539.2670	-0.0006	6	4	2	5	5	4	1	4
6542.2176	6542.2400	-0.0224	6	2	5	7	5	1	4	6
6542.4515	6542.4639	-0.0124	6	2	5	6	5	1	4	5
6686.7459	6686.7423	0.0036	4	4	1	5	3	3	0	4
6687.0191	6687.0213	-0.0022	4	4	1	4	3	3	0	3
6693.9770	6693.9708	0.0062	4	4	0	5	3	3	1	4
6694.0669	6694.0684	-0.0015	4	4	0	3	3	3	1	2
6694.2388	6694.2308	0.0080	4	4	0	4	3	3	1	3
6703.9151	6703.9004	0.0147	6	3	3	6	5	3	2	6
6704.2626	6704.2527	0.0099	6	3	3	6	5	3	2	5
6704.7370	6704.7065	0.0305	6	3	3	7	5	3	2	6
6753.0013	6753.0043	-0.0030	6	2	4	6	5	2	3	6
6754.1136	6754.1014	0.0122	6	2	4	6	5	2	3	5
6754.2148	6754.2138	0.0010	6	2	4	7	5	2	3	6
6754.2148	6754.1944	0.0204	6	2	4	5	5	2	3	4
6755.5149	6755.5155	-0.0006	6	2	4	5	5	2	3	5
6785.7342	6785.7247	0.0095	4	3	1	3	3	1	2	2
6786.2218	6786.2165	0.0053	4	3	1	5	3	1	2	4
6786.7697	6786.7913	-0.0216	7	0	7	8	6	1	6	7
6786.7697	6786.7616	0.0081	7	0	7	7	6	1	6	6
6786.7697	6786.7654	0.0043	7	0	7	6	6	1	6	5
6787.7325	6787.7262	0.0063	4	3	1	4	3	1	2	3
6788.0085	6787.9944	0.0141	7	1	7	7	6	1	6	7
6789.2255	6789.2440	-0.0185	7	1	7	8	6	1	6	7
6789.2255	6789.2177	0.0078	7	1	7	7	6	1	6	6
6789.2255	6789.2176	0.0079	7	1	7	6	6	1	6	5
6793.6353	6793.6459	-0.0106	7	0	7	8	6	0	6	7
6793.6353	6793.6271	0.0082	7	0	7	7	6	0	6	6
6793.6353	6793.6182	0.0171	7	0	7	6	6	0	6	5
6796.0952	6796.0985	-0.0033	7	1	7	8	6	0	6	7
6796.0952	6796.0832	0.0120	7	1	7	7	6	0	6	6
6796.0952	6796.0703	0.0249	7	1	7	6	6	0	6	5
6813.5225	6813.5258	-0.0033	5	3	3	4	4	2	2	3
6813.6890	6813.6938	-0.0048	5	3	3	6	4	2	2	5
6814.2993	6814.3038	-0.0045	5	3	3	5	4	2	2	4
7146.4657	7146.4873	-0.0216	7	1	6	7	6	2	5	6
7146.6339	7146.6464	-0.0125	7	1	6	8	6	2	5	7
7146.6339	7146.6452	-0.0113	7	1	6	6	6	2	5	5
7219.0140	7219.0413	-0.0273	7	2	6	6	6	2	5	5
7302.4465	7302.4301	0.0164	7	1	6	8	6	1	5	7
7303.7388	7303.7546	-0.0158	7	1	6	6	6	1	5	6
7374.8223	7374.8378	-0.0155	7	2	6	8	6	1	5	7

7374.9254	7374.9429	-0.0175	7	2	6	7	6	1	5	6
7386.0282	7386.0385	-0.0103	5	3	2	6	4	2	3	5
7386.2596	7386.2498	0.0098	5	3	2	5	4	2	3	4
7386.6794	7386.6745	0.0049	5	3	2	4	4	2	3	4
7513.6462	7513.6634	-0.0172	7	3	5	7	6	3	4	6
7513.8732	7513.8822	-0.0090	7	3	5	8	6	3	4	7
7513.8732	7513.8994	-0.0262	7	3	5	6	6	3	4	5
7590.9560	7590.9390	0.0170	7	5	3	7	6	5	2	6
7591.6316	7591.6359	-0.0043	7	5	3	8	6	5	2	7
7591.7596	7591.7642	-0.0046	7	5	3	6	6	5	2	5
7597.2340	7597.2334	0.0006	7	5	2	7	6	5	1	6
7597.9136	7597.9369	-0.0233	7	5	2	8	6	5	1	7
7606.7646	7606.7684	-0.0038	7	4	4	7	6	4	3	6
7607.2299	7607.2112	0.0187	7	4	4	8	6	4	3	7
7635.5571	7635.5728	-0.0157	6	3	4	5	5	2	3	4
7635.6839	7635.6982	-0.0143	6	3	4	7	5	2	3	6
7636.2039	7636.2142	-0.0103	6	3	4	6	5	2	3	5
7695.8712	7695.8535	0.0177	7	4	3	7	6	4	2	6
7726.7170	7726.7274	-0.0104	8	0	8	9	7	1	7	8
7726.7170	7726.7067	0.0103	8	0	8	8	7	1	7	7
7726.7170	7726.7071	0.0099	8	0	8	7	7	1	7	6
7727.5699	7727.5759	-0.0060	8	1	8	9	7	1	7	8
7727.5699	7727.5562	0.0137	8	1	8	8	7	1	7	7
7727.5699	7727.5554	0.0145	8	1	8	7	7	1	7	6
7729.1722	7729.1800	-0.0078	8	0	8	9	7	0	7	8
7730.0245	7730.0285	-0.0040	8	1	8	9	7	0	7	8
7730.0245	7730.0123	0.0122	8	1	8	8	7	0	7	7
7730.0245	7730.0075	0.0170	8	1	8	7	7	0	7	6
7739.5080	7739.5299	-0.0219	5	4	2	6	4	3	1	5
7739.9667	7739.9698	-0.0031	5	4	2	5	4	3	1	4
7740.3879	7740.3652	0.0227	5	4	2	5	4	3	1	5
7789.6118	7789.6292	-0.0174	5	4	1	4	4	3	2	3
7790.0434	7790.0397	0.0037	5	4	1	5	4	3	2	4
7796.1998	7796.2038	-0.0040	5	2	3	6	4	1	4	5
7799.0164	7799.0190	-0.0026	7	2	5	7	6	2	4	7
7800.2316	7800.2284	0.0032	7	2	5	7	6	2	4	6
7800.2316	7800.2163	0.0153	7	2	5	6	6	2	4	5
7801.6281	7801.6303	-0.0022	7	2	5	6	6	2	4	6
7841.6524	7841.6661	-0.0137	5	2	3	5	4	0	4	4
7866.5054	7866.4970	0.0084	5	3	2	4	4	1	3	3
7866.7450	7866.7364	0.0086	5	3	2	6	4	1	3	5
7867.6052	7867.5943	0.0109	5	3	2	5	4	1	3	4
7897.8218	7897.7944	0.0274	7	3	4	7	6	3	3	6
7898.0931	7898.0768	0.0163	7	3	4	6	6	3	3	5

Table S50. Transition frequencies for $\mathscr{C}_{1w}$: O(29). J is the rotational angular momentum quantum number, K_a and K_c are the projections of J onto the principal axes at the prolate and oblate symmetric top limits, and F is the total angular momentum quantum number, which includes the nuclear spin, I(^{14}N) = 1.

Observed Frequency /MHz	Calculated Frequency /MHz	Difference / MHz	J	K_a	K_c	F	J'	K'_a	K'_c	F'
2038.2124	2038.2179	-0.0055	2	0	2	3	1	0	1	2
2196.4773	2196.4719	0.0054	2	1	1	3	1	1	0	2
2899.8207	2899.8196	0.0011	3	1	3	3	2	1	2	2
2900.2008	2900.2001	0.0007	3	1	3	4	2	1	2	3
2987.4897	2987.4892	0.0005	3	0	3	2	2	0	2	1
2987.8140	2987.7962	0.0178	3	0	3	4	2	0	2	3
3105.2058	3105.2099	-0.0041	3	2	2	3	2	2	1	2
3106.6955	3106.6981	-0.0026	3	2	2	4	2	2	1	3
3107.5254	3107.5261	-0.0007	3	2	2	2	2	2	1	1
3218.8965	3218.8883	0.0082	2	2	0	3	1	1	1	2
3223.7129	3223.7157	-0.0028	3	2	1	3	2	2	0	2
3225.3187	3225.3243	-0.0056	3	2	1	4	2	2	0	3
3226.1596	3226.1722	-0.0126	3	2	1	2	2	2	0	1
3270.0429	3270.0475	-0.0046	3	1	2	3	2	1	1	2
3270.4094	3270.4189	-0.0095	3	1	2	4	2	1	1	3
3839.0383	3839.0435	-0.0052	4	1	4	4	3	1	3	3
3839.1808	3839.1913	-0.0105	4	1	4	5	3	1	3	4
3896.7492	3896.7360	0.0132	4	0	4	3	3	0	3	2
3896.8892	3896.8697	0.0195	4	0	4	5	3	0	3	4
3971.0062	3971.0130	-0.0068	3	2	2	4	2	1	1	3
4115.4432	4115.4446	-0.0014	4	2	3	4	3	2	2	3
4116.0475	4116.0486	-0.0011	4	2	3	5	3	2	2	4
4116.1884	4116.2046	-0.0162	4	2	3	3	3	2	2	3
4192.8989	4192.8943	0.0046	4	3	2	4	3	3	1	3
4194.8380	4194.8450	-0.0070	4	3	2	3	3	3	1	2
4224.7099	4224.7161	-0.0062	4	3	1	4	3	3	0	3
4226.1738	4226.1789	-0.0051	4	3	1	5	3	3	0	4
4226.7255	4226.7305	-0.0050	4	3	1	3	3	3	0	2
4308.2795	4308.2807	-0.0012	4	1	3	4	3	1	2	3
4308.3842	4308.3820	0.0022	4	1	3	5	3	1	2	4
4359.5590	4359.5614	-0.0024	4	2	2	4	3	2	1	3
4360.2407	4360.2419	-0.0012	4	2	2	5	3	2	1	4
4360.3932	4360.3953	-0.0021	4	2	2	3	3	2	1	2
4744.5192	4744.4984	0.0208	5	0	5	5	4	1	4	4
4744.6348	4744.6097	0.0251	5	0	5	6	4	1	4	5
4794.9521	4794.9517	0.0004	5	0	5	6	4	0	4	5
4816.3723	4816.3660	0.0063	4	2	3	3	3	1	2	2
4816.6431	4816.6427	0.0004	4	2	3	5	3	1	2	4
4817.2761	4817.2731	0.0030	4	2	3	4	3	1	2	3
4846.4784	4846.4734	0.0050	3	3	1	2	2	2	0	1
4846.8893	4846.8905	-0.0012	3	3	1	3	2	2	0	2
4882.6033	4882.6093	-0.0060	3	3	0	2	2	2	1	2
4884.7626	4884.7567	0.0059	3	3	0	4	2	2	1	3
4884.9310	4884.9245	0.0065	3	3	0	2	2	2	1	1
4885.1565	4885.1515	0.0050	3	3	0	3	2	2	1	2
5104.6511	5104.6569	-0.0058	5	2	4	5	4	2	3	4
5104.9389	5104.9460	-0.0071	5	2	4	6	4	2	3	5
5243.6310	5243.6351	-0.0041	5	3	3	5	4	3	2	4
5292.0588	5292.0701	-0.0113	5	1	4	6	4	1	3	5

5343.2499	5343.2498	0.0001	5	3	2	5	4	3	1	4
5344.0555	5344.0576	-0.0021	5	3	2	6	4	3	1	5
5344.2416	5344.2445	-0.0029	5	3	2	4	4	3	1	3
5480.8795	5480.8747	0.0048	5	2	3	5	4	2	2	4
5481.1919	5481.1855	0.0064	5	2	3	6	4	2	2	5
5683.5916	5683.6000	-0.0084	6	1	6	6	5	1	5	5
5683.5916	5683.6016	-0.0100	6	1	6	5	5	1	5	4
5696.4885	5696.4725	0.0160	6	0	6	7	5	0	5	6
5704.6018	5704.5829	0.0189	6	1	6	7	5	0	5	6
5815.1400	5815.1462	-0.0062	4	3	2	3	3	2	1	2
5815.3515	5815.3520	-0.0005	4	3	2	5	3	2	1	4
5816.0742	5816.0691	0.0051	4	3	2	4	3	2	1	3
6004.1272	6004.1289	-0.0017	4	3	1	3	3	2	2	2
6004.2420	6004.2374	0.0046	4	3	1	5	3	2	2	4
6004.6573	6004.6577	-0.0004	4	3	1	4	3	2	2	3
6071.6614	6071.6760	-0.0146	6	2	5	6	5	2	4	5
6071.8146	6071.8247	-0.0101	6	2	5	7	5	2	4	6
6217.7630	6217.7511	0.0119	6	1	5	7	5	1	4	6
6281.4638	6281.4736	-0.0098	6	3	4	6	5	3	3	5
6281.8863	6281.8773	0.0090	6	3	4	7	5	3	3	6
6315.8235	6315.8336	-0.0101	6	4	3	6	5	4	2	5
6316.5841	6316.5915	-0.0074	6	4	3	7	5	4	2	6
6316.7476	6316.7506	-0.0030	6	4	3	5	5	4	2	4
6341.5681	6341.5757	-0.0076	6	4	2	6	5	4	1	5
6342.3557	6342.3636	-0.0079	6	4	2	7	5	4	1	6
6342.5241	6342.5273	-0.0032	6	4	2	5	5	4	1	4
6497.8677	6497.8608	0.0069	6	3	3	6	5	3	2	5
6498.3815	6498.3587	0.0228	6	3	3	7	5	3	2	6
6562.1335	6562.1335	0.0000	6	2	4	6	5	2	3	5
6562.2618	6562.2601	0.0017	6	2	4	7	5	2	3	6
6597.2107	6597.2232	-0.0125	7	1	7	8	6	1	6	7
6602.3396	6602.3245	0.0151	7	0	7	7	6	0	6	6
6605.3411	6605.3336	0.0075	7	1	7	8	6	0	6	7
6606.4466	6606.4258	0.0208	4	4	0	5	3	3	1	4
6934.1090	6934.1200	-0.0110	7	1	6	8	6	2	5	7
7018.4191	7018.4412	-0.0221	7	2	6	6	6	2	5	5
7299.4227	7299.4395	-0.0168	7	3	5	7	6	3	4	6
7299.6655	7299.6752	-0.0097	7	3	5	8	6	3	4	7
7382.7100	7382.7188	-0.0088	7	4	4	7	6	4	3	6
7383.2108	7383.1973	0.0135	7	4	4	8	6	4	3	7
7459.7812	7459.7747	0.0065	7	4	3	7	6	4	2	6
7460.3275	7460.3124	0.0151	7	4	3	8	6	4	2	7
7507.6296	7507.6135	0.0161	8	0	8	8	7	1	7	7
7508.7032	7508.6835	0.0197	8	1	8	8	7	1	7	7
7510.6296	7510.6271	0.0025	8	0	8	9	7	0	7	8
7511.7010	7511.6956	0.0054	8	1	8	9	7	0	7	8
7586.9457	7586.9613	-0.0156	7	2	5	8	6	2	4	7
7623.8314	7623.8463	-0.0149	5	4	2	6	4	3	1	5
7624.3143	7624.3190	-0.0047	5	4	2	5	4	3	1	4
7658.9709	7658.9475	0.0234	7	3	4	7	6	3	3	6
7659.2738	7659.2449	0.0289	7	3	4	8	6	3	3	7
7668.1774	7668.1754	0.0020	5	4	1	6	4	3	2	5
7668.5565	7668.5654	-0.0089	5	4	1	5	4	3	2	4
7912.2273	7912.2487	-0.0214	8	1	7	7	7	2	6	6
7949.3078	7949.3214	-0.0136	8	2	7	9	7	2	6	8

7996.5784	7996.5938	-0.0154	8	1	7	9	7	1	6	8

3.3 Clusters with 2 water molecules

Table S51. Kraitchman Analysis for $\mathcal{O}_{2w}$. The experimental coordinates obtained through the Kraitchman equations, a, b, c are represented in their absolute value. The theoretical coordinates predicted for the different energy minima (M), transition-state (TS) and midpoints are also presented as well as the corresponding absolute deviation from the experimental value. Theoretical coordinates are presented in the Principal Axis System.

	Coordinate	Experimental Kraitchman	M_1		M_2		TS		Midpoint (M_1,M_2)	
			Value	Deviation	Value	Deviation	Value	Deviation	Value	Deviation
O(29)	|a| /Å	3.6596(5)	3.7296	0.0700	3.7437	0.0841	3.7069	0.0473	3.7367	0.0771
	|b| /Å	1.713(1)	-1.7001	0.0124	-1.6960	0.0166	-1.7189	0.0064	-1.6980	0.0145
	|c| /Å	0.12(1)	-0.2269	0.1056	0.0243	0.0970	0.0592	0.0621	-0.101	0.020
O(32)	|a| /Å	4.7018(3)	4.72782	0.02607	4.71604	0.0143	4.67576	0.02599	4.72193	0.02018
	|b| /Å	0.830(2)	0.8606	0.0319	0.8726	0.0439	0.8459	0.0172	0.8666	0.0379
	|c| /Å	0.14(1)	0.016	0.813	-0.239	0.104	-0.195	0.060	-0.111	0.024

3.3.1 Equilibrium and transition state geometries

Table S52. Equilibrium geometry of $\mathscr{O}_{2w}$ (M_1).

Atom	x /Å	y /Å	z /Å
C	-1.29223565797074	1.15231183648478	1.60661735071153
C	-1.97000266142166	-0.24723177125698	1.65700811959158
C	-1.83600069808222	-0.80487894320504	0.20762957357547
C	-0.35174759739900	-1.13433014285037	0.09384767412722
C	0.31733619305030	0.21143096423071	0.06980149620444
C	-0.78183849771428	1.23184359138603	0.14673183846569
C	-2.75899776353538	-1.95541395062687	-0.12584597314810
C	-1.90135695012951	0.49969553141572	-0.64927601762525
C	-1.53334193134964	0.27678335880285	-2.11933547989400
C	-3.25323679052148	1.21070629369292	-0.59304882709986
O	0.17591776815757	-2.22715155314974	0.05532450712080
N	1.59430497230122	0.30707172125183	0.02734600064980
O	2.04327315530997	1.60841709950423	0.04612454427476
H	-2.00390743007213	1.95405032505202	1.81379647132314
H	-0.47136857092656	1.24937113132735	2.32032213341473
H	-1.49938027440357	-0.91752455673581	2.38200090930807
H	-3.02773816052576	-0.17831262534151	1.92375877207396
H	-0.50528535160372	2.22723577956892	-0.20153795492531
H	-2.60443950653384	-2.30491779254306	-1.14980423855373
H	-2.56547283256408	-2.80029910456063	0.54010583076416
H	-3.80576677117543	-1.66148313820478	-0.01091315407823
H	-0.57736373886957	-0.23907331021594	-2.24713167727069
H	-2.30237954779227	-0.31619086413880	-2.62251275463932
H	-1.46168414083018	1.23766896199687	-2.63819993885542
H	-3.62090429617380	1.36721443076076	0.42151410268626
H	-3.17668839177188	2.19025663622608	-1.07580783369363
H	-4.00740527550703	0.63397036549499	-1.13621625040959
H	3.02419130598347	1.49316562112034	0.02827375299797
O	3.75343275762446	-1.64789087422957	-0.27550141738601
H	2.85762918060066	-1.26733158852998	-0.21076778028519
H	3.71495570300368	-2.47193934087737	0.22071841800942
O	4.68788900157801	0.92620014509875	0.06129792194108
H	4.58051675481162	-0.04631727602409	0.02216922836159
H	5.27552965845284	1.14561080517538	-0.66943046363732

Table S53. Equilibrium geometry of $\mathscr{O}_{2w}$ (M_2).

Atom	x /Å	y /Å	z /Å
C	-1.31442276110781	1.11240447339365	1.63239445549770
C	-1.98619637155562	-0.29095353506017	1.63820347831847
C	-1.85490746991416	-0.79979472893116	0.17089058137939
C	-0.37091714251181	-1.12142041902217	0.03821367473467
C	0.29575094259945	0.22560844174987	0.06795071514273
C	-0.80568171724039	1.24087414498928	0.17532400940125
C	-2.77703567716436	-1.93974151254597	-0.19914487780943
C	-1.92468115759874	0.53291679262792	-0.64241958545730
C	-1.55815737263345	0.36104991127555	-2.11962544569997
C	-3.27870146357449	1.23731351500040	-0.56033516127826
O	0.15807007126003	-2.20951725792042	-0.06353079116729
N	1.57252364503296	0.32467002492449	0.03013694468545
O	2.01920156002204	1.62626910142197	0.07691443744204
H	-2.02924119845837	1.90415260430933	1.86556466697311
H	-0.49354469662813	1.18959641759350	2.34853697504272
H	-1.51008744520979	-0.98271375647692	2.33904984027376
H	-3.04321635358449	-0.23551262173746	1.91081299977707
H	-0.53114380853849	2.24751630271953	-0.14067917797941
H	-2.62408552544657	-2.25443852803794	-1.23455837034153
H	-2.58110142976523	-2.80633186237418	0.43759352937807
H	-3.82396036379735	-1.65133512210175	-0.07234997077459
H	-0.59790068651091	-0.14179863512881	-2.26578925340174
H	-2.32318448429868	-0.22157025266443	-2.64070754886396
H	-1.49535424105337	1.33898667646352	-2.60671982586110
H	-3.64671715039286	1.35572357327464	0.45925805071290
H	-3.20531254560720	2.23389901323277	-1.00739873150434
H	-4.03113057643433	0.67818435614674	-1.12393601469929
H	2.99767730158786	1.51618679117352	-0.00213813116098
O	3.74750760705212	-1.63248163397873	0.02739783949564
H	2.84889161416306	-1.25404754160842	0.05631069896039
H	3.67098439678013	-2.42610831315758	-0.51215031981092
O	4.65523333919276	0.96263350349789	-0.20413795568042
H	4.54858655311370	-0.01038388235792	-0.23006328148856
H	5.29077460822250	1.12811395930944	0.50025154576370

Table S54. Transition-state geometry of $\mathscr{O}_{2w}$ (TS$_1$).

Atom	x /Å	y /Å	z /Å
C	-0.86759679080333	1.06211285893049	1.69879316220748
C	-1.52290346079085	-0.34890231254242	1.68252068899337
C	-1.37632474341603	-0.83759564343740	0.20977206606020
C	0.11195652779165	-1.14129920504447	0.08316449056931
C	0.76348922116286	0.21244470980649	0.13612738360345
C	-0.35016582755770	1.21407672861914	0.24708068502077
C	-2.28328319704208	-1.98275532630520	-0.18116558589200
C	-1.45535764054235	0.50426831615021	-0.58727916957375
C	-1.07667261192892	0.35507332078459	-2.06387256343683
C	-2.81769536826775	1.19248395105518	-0.50584344274684
O	0.65305057480918	-2.22239459204305	-0.02988468169647
N	2.03966719988605	0.32564504457968	0.11513074415767
O	2.46991214939744	1.63160076824270	0.18484378106189
H	-1.59336178572631	1.84263696981854	1.93607933107629
H	-0.05274025120033	1.14013699835772	2.42164108954333
H	-1.04288986436757	-1.04385842773026	2.37750607403783
H	-2.58221216261411	-0.30949310201255	1.94909532784458
H	-0.08471926183345	2.22763078899359	-0.05443745762027
H	-2.11984006582588	-2.28244315071737	-1.21944331009809
H	-2.08234959403644	-2.85531940855599	0.44577390783594
H	-3.33417769835095	-1.70737127071506	-0.05807933603530
H	-0.11000134667461	-0.13555019526216	-2.20934742345340
H	-1.83164470177348	-0.22909656152967	-2.59776158202322
H	-1.02117137043081	1.33976129428425	-2.53820338324744
H	-3.19508448243251	1.29134604602922	0.51241561053720
H	-2.75186376680685	2.19651902971459	-0.93721235467933
H	-3.55945545926491	0.63372355547982	-1.08380938423492
H	3.44685447244392	1.53310829450231	0.12438639246625
O	4.16441974252706	-1.71125459634768	0.20418358758696
H	3.29285975062191	-1.27389794831126	0.19523533524564
H	4.03843972729208	-2.52392272556535	-0.29600765416540
O	5.08649157937186	0.87539984448694	0.00259002523707
H	5.04462136643803	-0.09994389414279	-0.00759703067623
H	5.99974913994516	1.12712984042723	-0.15639532350572

Table S55. Equilibrium geometry of $\mathscr{C}_{2w}$.

Atom	x /Å	y /Å	z /Å
C	-0.64992040385882	0.45143448697203	2.04990102517603
C	-2.04309410838574	0.01937751767371	1.51071762042038
C	-1.87085641084098	-0.01667552295615	-0.03903992897178
C	-0.99632204186567	-1.23344778767441	-0.25583463566963
C	0.35555769295015	-0.83551434597304	0.25803011766632
C	0.20121504455165	0.57007517058340	0.76302410735405
C	-3.15813848909544	-0.01528649064718	-0.83312053340271
C	-0.82042967439531	1.12015819266167	-0.27607006978156
C	-0.26002128904614	1.14515385023618	-1.70195599045323
C	-1.32957835552652	2.52031335306796	0.06259270066433
O	-1.29149177189749	-2.31843565986480	-0.73802882272228
N	1.45072337062323	-1.51199992099310	0.23943484768479
O	1.38470924035786	-2.78618897364391	-0.28647413221388
H	-0.69880778737092	1.41007619033749	2.56943519762623
H	-0.22448445732962	-0.27630485181372	2.74464321682204
H	-2.36664384955277	-0.95141054735051	1.89757283919618
H	-2.82247212731039	0.74177101158614	1.76534803714500
H	1.12860277415789	1.13401954702881	0.85333502732833
H	-2.96144670011835	-0.05114822648153	-1.90758010279795
H	-3.76515492790846	-0.88837989953543	-0.57945068961313
H	-3.74276523939788	0.88226193402688	-0.61599204467482
H	-1.02320230424971	1.49076399382476	-2.40537303807514
H	0.59128668957877	1.82915015023959	-1.75008215861990
H	0.08399231938455	0.16448601220881	-2.04579508008844
H	-0.49387577876443	3.22493110567364	0.01970634650297
H	-2.07469614781082	2.84082407339757	-0.67128344462816
H	-1.78271363403452	2.59348923479646	1.05200219894857
H	0.45261458548673	-2.94890195891874	-0.55233457354543
O	3.97274803942013	-0.02987374664274	0.09520500984000
H	3.21494633512836	-0.62000240744576	0.26996682815640
H	4.50929869554871	-0.50531847910190	-0.54802551063450
O	2.75364658394473	2.49937649720556	-0.38897226456064
H	3.26278075371939	1.67482106309653	-0.27937712110831
H	3.32780400100784	3.18821156442569	-0.03896141977013

3.3.2 Transition frequencies

Table S56. Transition frequencies for $\mathscr{O}_{2w}$. J is the rotational angular momentum quantum number, K_a and K_c are the projections of J onto the principal axes at the prolate and oblate symmetric top limits, and F is the total angular momentum quantum number, which includes the nuclear spin, I(^{14}N) = 1.

Observed Frequency /MHz	Calculated Frequency /MHz	Difference / MHz	J	K_a	K_c	F	J'	K'_a	K'_c	F'
2079.8596	2079.8633	-0.0037	3	1	2	3	2	1	1	2
2080.1188	2080.1151	0.0037	3	1	2	4	2	1	1	3
2607.7347	2607.7329	0.0018	4	1	4	4	3	1	3	3
2607.8620	2607.8518	0.0102	4	1	4	5	3	1	3	4
2607.8645	2607.8518	0.0127	4	1	4	5	3	1	3	4
2673.5427	2673.5493	-0.0066	4	0	4	3	3	0	3	2
2691.5287	2691.5236	0.0051	4	2	3	4	3	2	2	3
2691.9093	2691.9131	-0.0038	4	2	3	5	3	2	2	4
2692.0231	2692.0130	0.0101	4	2	3	3	3	2	2	2
2711.2799	2711.2953	-0.0154	4	2	2	4	3	2	1	3
2711.6626	2711.6719	-0.0093	4	2	2	5	3	2	1	4
2770.8412	2770.8276	0.0136	4	1	3	4	3	1	2	3
2770.9614	2770.9414	0.0200	4	1	3	5	3	1	2	4
3118.1114	3118.1094	0.0020	4	1	4	3	3	0	3	2
3118.2064	3118.2021	0.0043	4	1	4	5	3	0	3	4
3118.2064	3118.2139	-0.0075	4	1	4	4	3	0	3	3
3198.7648	3198.7783	-0.0135	2	2	1	3	1	1	0	2
3256.3597	3256.3580	0.0017	5	1	5	5	4	1	4	4
3256.4303	3256.4255	0.0048	5	1	5	6	4	1	4	5
3362.1170	3362.1106	0.0064	5	2	4	5	4	2	3	4
3362.3279	3362.3160	0.0119	5	2	4	6	4	2	3	5
3400.8364	3400.8240	0.0124	5	2	3	5	4	2	2	4
3401.0065	3401.0139	-0.0074	5	2	3	6	4	2	2	5
3459.4424	3459.4389	0.0035	5	1	4	4	4	1	3	3
3701.0029	3700.9975	0.0054	5	1	5	6	4	0	4	5
3831.1098	3831.1020	0.0078	3	2	2	2	2	1	1	1
3831.3140	3831.2920	0.0220	3	2	2	4	2	1	1	3
3831.6412	3831.6332	0.0080	3	2	2	3	2	1	1	2
3903.0216	3903.0319	-0.0103	6	1	6	6	5	1	5	5
3963.5111	3963.5042	0.0069	3	2	1	2	2	1	2	1
3963.8385	3963.8284	0.0101	3	2	1	4	2	1	2	3
3964.4187	3964.4173	0.0014	3	2	1	3	2	1	2	2
3974.6220	3974.6320	-0.0100	6	0	6	6	5	0	5	5
3974.6220	3974.6290	-0.0070	6	0	6	5	5	0	5	4
4030.9638	4030.9564	0.0074	6	2	5	6	5	2	4	5
4031.0780	4031.0799	-0.0019	6	2	5	7	5	2	4	6
4049.2423	4049.2398	0.0025	6	3	4	6	5	3	3	5
4049.4785	4049.4948	-0.0163	6	3	4	7	5	3	3	6
4052.6479	4052.6509	-0.0030	6	3	3	6	5	3	2	5
4052.8818	4052.9041	-0.0223	6	3	3	7	5	3	2	6
4096.3068	4096.3112	-0.0044	6	2	4	6	5	2	3	5
4096.4231	4096.4175	0.0056	6	2	4	7	5	2	3	6
4312.7066	4312.7012	0.0054	7	0	7	7	6	1	6	6
4312.7066	4312.6975	0.0091	7	0	7	6	6	1	6	5
4443.2987	4443.2935	0.0052	4	2	3	4	3	1	2	3
4547.6651	4547.6624	0.0027	7	1	7	7	6	1	6	6
4547.6651	4547.6721	-0.0070	7	1	7	6	6	1	6	5

4614.0995	4614.0961	0.0034	7	0	7	7	6	0	6	6
4614.0995	4614.1000	-0.0005	7	0	7	6	6	0	6	5
4697.8640	4697.8815	-0.0175	7	2	6	8	6	2	5	7
4726.2908	4726.2816	0.0092	7	3	5	7	6	3	4	6
4726.4478	4726.4440	0.0038	7	3	5	8	6	3	4	7
4733.8831	4733.8787	0.0044	7	3	4	7	6	3	3	6
4734.0448	4734.0383	0.0065	7	3	4	8	6	3	3	7
4796.9455	4796.9565	-0.0110	7	2	5	8	6	2	4	7
4826.1452	4826.1483	-0.0031	7	1	6	8	6	1	5	7
4849.0711	4849.0951	-0.0240	7	1	7	8	6	0	6	7
5012.9851	5012.9743	0.0108	8	0	8	8	7	1	7	7
5012.9851	5012.9678	0.0173	8	0	8	7	7	1	7	6
5012.9874	5012.9864	0.0010	8	0	8	9	7	1	7	8
5034.4520	5034.4645	-0.0125	5	2	4	6	4	1	3	5
5190.2751	5190.2862	-0.0111	8	1	8	9	7	1	7	8
5247.9504	5247.9593	-0.0089	8	0	8	9	7	0	7	8
5362.4157	5362.4261	-0.0104	8	2	7	9	7	2	6	8
5400.4406	5400.4452	-0.0046	8	4	5	8	7	4	4	7
5400.6313	5400.6366	-0.0053	8	4	5	9	7	4	4	8
5400.9729	5400.9571	0.0158	8	4	4	8	7	4	3	7
5403.5543	5403.5586	-0.0043	8	3	6	8	7	3	5	7
5403.6733	5403.6687	0.0046	8	3	6	9	7	3	5	8
5418.5085	5418.5137	-0.0052	8	3	5	8	7	3	4	7
5418.6247	5418.6236	0.0011	8	3	5	7	7	3	4	6
5418.6249	5418.6195	0.0054	8	3	5	9	7	3	4	8
5502.2369	5502.2390	-0.0021	8	1	7	9	7	1	6	8
5511.0860	5511.0758	0.0102	5	2	3	6	4	1	4	5
5511.4777	5511.4774	0.0003	5	2	3	5	4	1	4	4
5700.9183	5700.9247	-0.0064	9	0	9	10	8	1	8	9
5700.9183	5700.9156	0.0027	9	0	9	9	8	1	8	8
5809.3905	5809.4080	-0.0175	4	3	2	5	3	2	1	4
5830.9505	5830.9611	-0.0106	9	1	9	10	8	1	8	9
5878.2170	5878.2245	-0.0075	9	0	9	10	8	0	8	9
6008.2618	6008.2609	0.0009	9	1	9	10	8	0	8	9
6008.2618	6008.2487	0.0131	9	1	9	8	8	0	8	7
6078.6920	6078.6851	0.0069	9	4	6	9	8	4	5	8
6078.8382	6078.8202	0.0180	9	4	6	10	8	4	5	9
6079.8955	6079.9054	-0.0099	9	4	5	9	8	4	4	8
6080.0383	6080.0401	-0.0018	9	4	5	10	8	4	4	9
6080.7866	6080.8051	-0.0185	9	3	7	10	8	3	6	9
6107.5657	6107.5741	-0.0084	9	3	6	10	8	3	5	9
6159.1224	6159.1078	0.0146	7	2	6	8	6	1	5	7
6159.1224	6159.1222	0.0002	7	2	6	7	6	1	5	6
6171.9321	6171.9367	-0.0046	9	1	8	10	8	1	7	9
6205.0588	6205.0464	0.0124	9	2	7	10	8	2	6	9
6351.0448	6351.0679	-0.0231	6	2	4	7	5	1	5	6
6351.4228	6351.4305	-0.0077	6	2	4	6	5	1	5	5
6376.7802	6376.7801	0.0001	10	0	10	11	9	1	9	10
6376.7802	6376.7719	0.0083	10	0	10	10	9	1	9	9
6376.7802	6376.7681	0.0121	10	0	10	9	9	1	9	8
6469.8991	6469.9032	-0.0041	10	1	10	11	9	1	9	10
6470.8252	6470.8274	-0.0022	5	3	3	6	4	2	2	5
6471.0824	6471.0969	-0.0145	5	3	3	5	4	2	2	4
6500.5683	6500.5748	-0.0065	5	3	3	6	4	2	3	5
6502.2873	6502.2911	-0.0038	5	3	2	6	4	2	3	5

6502.6045	6502.5909	0.0136	5	3	2	5	4	2	3	4
6506.8149	6506.8165	-0.0016	10	0	10	11	9	0	9	10
6599.9222	6599.9396	-0.0174	10	1	10	11	9	0	9	10
6599.9222	6599.9136	0.0086	10	1	10	10	9	0	9	9
6599.9222	6599.9295	-0.0073	10	1	10	9	9	0	9	8
6683.7626	6683.7598	0.0028	10	2	9	11	9	2	8	10
6695.3813	6695.3856	-0.0043	8	2	7	9	7	1	6	8
6695.3813	6695.3715	0.0098	8	2	7	7	7	1	6	6
6757.4141	6757.4170	-0.0029	10	3	8	11	9	3	7	10
6757.9193	6757.9202	-0.0009	10	4	7	11	9	4	6	10
6760.4267	6760.4401	-0.0134	10	4	6	10	9	4	5	9
6760.5353	6760.5383	-0.0030	10	4	6	11	9	4	5	10
6801.7200	6801.7164	0.0036	10	3	7	10	9	3	6	9
6801.7877	6801.7667	0.0210	10	3	7	11	9	3	6	10
6834.1055	6834.1089	-0.0034	10	1	9	11	9	1	8	10
6907.6883	6907.6794	0.0089	10	2	8	11	9	2	7	10
7041.9554	7041.9551	0.0003	11	0	11	12	10	1	10	11
7107.3418	7107.3451	-0.0033	11	1	11	12	10	1	10	11
7119.3251	7119.3083	0.0168	6	3	4	7	5	2	3	6
7135.0750	7135.0782	-0.0032	11	0	11	12	10	0	10	11
7192.8696	7192.8793	-0.0097	6	3	3	7	5	2	4	6
7193.1467	7193.1312	0.0155	6	3	3	6	5	2	4	5
7200.4771	7200.4682	0.0089	11	1	11	12	10	0	10	11
7200.4771	7200.4596	0.0175	11	1	11	10	10	0	10	9
7217.6019	7217.6022	-0.0003	9	2	8	10	8	1	7	9
7340.1791	7340.1903	-0.0112	11	2	10	12	10	2	9	11
7422.9610	7422.9700	-0.0090	11	6	5	11	10	6	4	10
7422.9610	7422.9666	-0.0056	11	6	6	11	10	6	5	10
7423.1309	7423.1363	-0.0054	11	6	5	12	10	6	4	11
7433.0376	7433.0263	0.0113	11	3	9	12	10	3	8	11
7437.8651	7437.8791	-0.0140	11	4	8	12	10	4	7	11
7437.8651	7437.8807	-0.0156	11	4	8	10	10	4	7	9
7443.0366	7443.0471	-0.0105	11	4	7	12	10	4	6	11
7487.9262	7487.9275	-0.0013	11	1	10	12	10	1	9	11
7501.7699	7501.7474	0.0225	11	3	8	12	10	3	7	11
7606.6258	7606.6106	0.0152	11	2	9	12	10	2	8	11
7698.3609	7698.3476	0.0133	12	0	12	12	11	1	11	11
7698.3609	7698.3472	0.0137	12	0	12	11	11	1	11	10
7743.5360	7743.5380	-0.0020	12	1	12	13	11	1	11	12
7763.7360	7763.7455	-0.0095	12	0	12	13	11	0	11	12
7808.9295	7808.9281	0.0014	12	1	12	13	11	0	11	12
7895.7870	7895.7888	-0.0018	7	3	4	6	6	2	5	5
7993.6610	7993.6727	-0.0117	12	2	11	13	11	2	10	12

Table S57. Transition frequencies for $\mathcal{O}_{2w}$:O(29). J is the rotational angular momentum quantum number, K_a and K_c are the projections of J onto the principal axes at the prolate and oblate symmetric top limits, and F is the total angular momentum quantum number, which includes the nuclear spin, I(^{14}N) = 1.

Observed Frequency /MHz	Calculated Frequency /MHz	Difference / MHz	J	K_a	K_c	F	J'	K_a'	K_c'	F'
2639.8348	2639.8484	-0.0136	4	2	3	4	3	2	2	3
2640.2531	2640.2357	0.0174	4	2	3	5	3	2	2	4
2640.3527	2640.3351	0.0176	4	2	3	3	3	2	2	2
2659.7852	2659.7844	0.0008	4	2	2	5	3	2	1	4
3192.6990	3192.6967	0.0023	5	1	5	5	4	1	4	4
3192.7582	3192.7608	-0.0026	5	1	5	6	4	1	4	5
3297.5471	3297.5437	0.0034	5	2	4	5	4	2	3	4
3297.7357	3297.7469	-0.0112	5	2	4	6	4	2	3	5
3335.8551	3335.8381	0.0170	5	2	3	5	4	2	2	4
3336.0250	3336.0355	-0.0105	5	2	3	6	4	2	2	5
3394.0673	3394.0564	0.0109	5	1	4	4	4	1	3	3
3826.6738	3826.6867	-0.0129	6	1	6	6	5	1	5	5
3897.7684	3897.7754	-0.0070	6	0	6	5	5	0	5	4
3953.5209	3953.5161	0.0048	6	2	5	6	5	2	4	5
3953.6426	3953.6373	0.0053	6	2	5	7	5	2	4	6
3953.6426	3953.6371	0.0055	6	2	5	5	5	2	4	4
4018.1835	4018.1780	0.0055	6	2	4	6	5	2	3	5
4018.2987	4018.2926	0.0061	6	2	4	7	5	2	3	6
4018.2987	4018.2919	0.0068	6	2	4	5	5	2	3	4
4458.6600	4458.6533	0.0067	7	1	7	7	6	1	6	6
4458.6600	4458.6609	-0.0009	7	1	7	6	6	1	6	5
4524.6758	4524.6837	-0.0079	7	0	7	8	6	0	6	7
4524.6758	4524.6684	0.0074	7	0	7	7	6	0	6	6
4524.6758	4524.6609	0.0149	7	0	7	6	6	0	6	5
4607.5732	4607.5804	-0.0072	7	2	6	6	6	2	5	5
4607.5763	4607.5866	-0.0103	7	2	6	8	6	2	5	7
4635.6793	4635.6894	-0.0101	7	3	5	7	6	3	4	6
4635.8513	4635.8525	-0.0012	7	3	5	8	6	3	4	7
4635.8513	4635.8673	-0.0160	7	3	5	6	6	3	4	5
4643.1772	4643.1870	-0.0098	7	3	4	7	6	3	3	6
4643.3497	4643.3492	0.0005	7	3	4	8	6	3	3	7
4643.3497	4643.3639	-0.0142	7	3	4	6	6	3	3	5
4705.6325	4705.6404	-0.0079	7	2	5	6	6	2	4	5
4705.6342	4705.6470	-0.0128	7	2	5	8	6	2	4	7
4734.7998	4734.7950	0.0048	7	1	6	6	6	1	5	5
4734.8029	4734.8123	-0.0094	7	1	6	8	6	1	5	7
4911.8708	4911.8848	-0.0140	8	0	8	9	7	1	7	8
4911.8708	4911.8563	0.0145	8	0	8	8	7	1	7	7
4911.8708	4911.8697	0.0011	8	0	8	7	7	1	7	6
5088.6141	5088.6292	-0.0151	8	1	8	9	7	1	7	8
5088.6141	5088.6086	0.0055	8	1	8	8	7	1	7	7
5088.6141	5088.6131	0.0010	8	1	8	7	7	1	7	6
5145.9645	5145.9740	-0.0095	8	0	8	9	7	0	7	8
5145.9645	5145.9604	0.0041	8	0	8	8	7	0	7	7
5259.2931	5259.3033	-0.0102	8	2	7	9	7	2	6	8
5259.2931	5259.2957	-0.0026	8	2	7	7	7	2	6	6
5300.0113	5300.0102	0.0011	8	3	6	8	7	3	5	7
5300.1317	5300.1209	0.0108	8	3	6	9	7	3	5	8
5300.1317	5300.1254	0.0063	8	3	6	7	7	3	5	6

5314.8906	5314.8849	0.0057	8	3	5	7	7	3	4	6
5314.8938	5314.8806	0.0132	8	3	5	9	7	3	4	8
5396.1720	5396.1624	0.0096	8	2	6	9	7	2	5	8
5397.9979	5398.0085	-0.0106	8	1	7	9	7	1	6	8
5397.9979	5397.9947	0.0032	8	1	7	7	7	1	6	6
5716.6673	5716.6769	-0.0096	9	1	9	10	8	1	8	9
5716.6673	5716.6611	0.0062	9	1	9	9	8	1	8	8
5716.6673	5716.6640	0.0033	9	1	9	8	8	1	8	7
5763.7015	5763.7077	-0.0062	9	0	9	10	8	0	8	9
5763.7015	5763.6956	0.0059	9	0	9	9	8	0	8	8
5763.7015	5763.6942	0.0073	9	0	9	8	8	0	8	7
5962.1967	5962.1880	0.0087	9	4	6	9	8	4	5	8
5962.3152	5962.3240	-0.0088	9	4	6	10	8	4	5	9
5962.3152	5962.3345	-0.0193	9	4	6	8	8	4	5	7
5964.3001	5964.3059	-0.0058	9	3	7	8	8	3	6	7
5964.3060	5964.3060	0.0000	9	3	7	10	8	3	6	9
5990.7317	5990.7361	-0.0044	9	3	6	10	8	3	5	9
6087.3675	6087.3657	0.0018	9	2	7	10	8	2	6	9
6087.3675	6087.3580	0.0095	9	2	7	8	8	2	6	7
6343.0018	6343.0066	-0.0048	10	1	10	11	9	1	9	10
6343.0018	6342.9939	0.0079	10	1	10	10	9	1	9	9
6343.0018	6342.9960	0.0058	10	1	10	9	9	1	9	8
6379.7642	6379.7670	-0.0028	10	0	10	11	9	0	9	10
6379.7642	6379.7564	0.0078	10	0	10	10	9	0	9	9
6379.7642	6379.7562	0.0080	10	0	10	9	9	0	9	8
6555.0698	6555.0628	0.0070	10	2	9	11	9	2	8	10
6555.0698	6555.0558	0.0140	10	2	9	9	9	2	8	8
6627.9693	6627.9748	-0.0055	10	3	8	9	9	3	7	8
6627.9709	6627.9769	-0.0060	10	3	8	11	9	3	7	10
6628.3375	6628.3455	-0.0080	10	4	7	10	9	4	6	9
6628.4417	6628.4501	-0.0084	10	4	7	9	9	4	6	8
6628.4418	6628.4454	-0.0036	10	4	7	11	9	4	6	10
6630.9325	6630.9248	0.0077	10	4	6	10	9	4	5	9
6631.0167	6631.0245	-0.0078	10	4	6	11	9	4	5	10
6631.0167	6631.0292	-0.0125	10	4	6	9	9	4	5	8
6671.7848	6671.7733	0.0115	10	3	7	11	9	3	6	10
6671.7848	6671.7710	0.0138	10	3	7	9	9	3	6	8
6776.8586	6776.8636	-0.0050	10	2	8	11	9	2	7	10
6776.8586	6776.8565	0.0021	10	2	8	9	9	2	7	8
6967.8473	6967.8479	-0.0006	11	1	11	12	10	1	10	11
6967.8473	6967.8374	0.0099	11	1	11	11	10	1	10	10
6967.8473	6967.8390	0.0083	11	1	11	10	10	1	10	9
6995.4802	6995.4876	-0.0074	11	0	11	12	10	0	10	11
6995.4802	6995.4782	0.0020	11	0	11	11	10	0	10	10
6995.4802	6995.4786	0.0016	11	0	11	10	10	0	10	9
7198.7389	7198.7495	-0.0106	11	2	10	12	10	2	9	11
7198.7389	7198.7254	0.0135	11	2	10	11	10	2	9	10
7198.7389	7198.7432	-0.0043	11	2	10	10	10	2	9	9
7345.4775	7345.4853	-0.0078	11	1	10	12	10	1	9	11
7345.4775	7345.4722	0.0053	11	1	10	11	10	1	9	10
7345.4775	7345.4778	-0.0003	11	1	10	10	10	1	9	9
7358.5510	7358.5419	0.0091	11	3	8	12	10	3	7	11
7358.5510	7358.5387	0.0123	11	3	8	10	10	3	7	9
7462.7115	7462.7161	-0.0046	11	2	9	12	10	2	8	11
7462.7115	7462.6974	0.0141	11	2	9	11	10	2	8	10

7462.7115	7462.7098	0.0017	11	2	9	10	10	2	8	9
7591.4383	7591.4494	-0.0111	12	1	12	13	11	1	11	12
7591.4383	7591.4406	-0.0023	12	1	12	12	11	1	11	11
7591.4383	7591.4419	-0.0036	12	1	12	11	11	1	11	10
7611.5990	7611.6039	-0.0049	12	0	12	13	11	0	11	12
7611.5990	7611.5956	0.0034	12	0	12	12	11	0	11	11
7611.5990	7611.5963	0.0027	12	0	12	11	11	0	11	10
7839.5156	7839.5170	-0.0014	12	2	11	13	11	2	10	12
7839.5156	7839.5115	0.0041	12	2	11	11	11	2	10	10
7963.1084	7963.1171	-0.0087	12	4	9	11	11	4	8	10
7963.1092	7963.1171	-0.0079	12	4	9	13	11	4	8	12
7972.4853	7972.4983	-0.0130	12	4	8	11	11	4	7	10
7978.1770	7978.1679	0.0091	12	1	11	13	11	1	10	12

Table S58. Transition frequencies for $\mathscr{O}_{2w}$:O(32). J is the rotational angular momentum quantum number, K_a and K_c are the projections of J onto the principal axes at the prolate and oblate symmetric top limits, and F is the total angular momentum quantum number, which includes the nuclear spin, I(^{14}N) = 1.

Observed Frequency /MHz	Calculated Frequency /MHz	Difference / MHz	J	K_a	K_c	F	J'	K'_a	K'_c	F'
2613.7847	2613.7944	-0.0097	4	2	3	4	3	2	2	3
2614.1612	2614.1621	-0.0009	4	2	3	5	3	2	2	4
2631.8076	2631.8115	-0.0039	4	2	2	5	3	2	1	4
3165.0161	3165.0218	-0.0057	5	1	5	6	4	1	4	5
3234.6284	3234.6277	0.0007	5	0	5	4	4	0	4	3
3300.0447	3300.0488	-0.0041	5	2	3	6	4	2	2	5
3357.9230	3357.9303	-0.0073	5	1	4	5	4	1	3	4
3357.9469	3357.9590	-0.0121	5	1	4	4	4	1	3	3
3357.9751	3357.9884	-0.0133	5	1	4	6	4	1	3	5
3793.8009	3793.8122	-0.0113	6	1	6	6	5	1	5	5
3864.3838	3864.3907	-0.0069	6	0	6	5	5	0	5	4
3915.0711	3915.0676	0.0035	6	2	5	6	5	2	4	5
3915.1794	3915.1814	-0.0020	6	2	5	7	5	2	4	6
3915.1794	3915.1811	-0.0017	6	2	5	5	5	2	4	4
3973.7896	3973.7868	0.0028	6	2	4	6	5	2	3	5
3973.9063	3973.9018	0.0045	6	2	4	7	5	2	3	6
3973.9063	3973.9015	0.0048	6	2	4	5	5	2	3	4
4420.7802	4420.7922	-0.0120	7	1	7	7	6	1	6	6
4420.7802	4420.7980	-0.0178	7	1	7	6	6	1	6	5
4487.3669	4487.3807	-0.0138	7	0	7	8	6	0	6	7
4487.3669	4487.3737	-0.0068	7	0	7	7	6	0	6	6
4487.3669	4487.3586	0.0083	7	0	7	6	6	0	6	5
4563.1967	4563.2078	-0.0111	7	2	6	8	6	2	5	7
4563.1967	4563.2018	-0.0051	7	2	6	6	6	2	5	5
4588.7094	4588.7146	-0.0052	7	3	5	7	6	3	4	6
4588.8853	4588.8707	0.0146	7	3	5	8	6	3	4	7
4588.8853	4588.8850	0.0003	7	3	5	6	6	3	4	5
4595.0868	4595.0876	-0.0008	7	3	4	7	6	3	3	6
4595.2587	4595.2440	0.0147	7	3	4	8	6	3	3	7
4595.2587	4595.2581	0.0006	7	3	4	6	6	3	3	5
4652.7097	4652.7216	-0.0119	7	2	5	8	6	2	4	7
4652.7097	4652.7155	-0.0058	7	2	5	6	6	2	4	5

4686.1019	4686.0999	0.0020	7	1	6	8	6	1	5	7
4686.1019	4686.0816	0.0203	7	1	6	6	6	1	5	5
5045.9029	5045.9123	-0.0094	8	1	8	9	7	1	7	8
5045.9029	5045.8977	0.0052	8	1	8	7	7	1	7	6
5104.7937	5104.7921	0.0016	8	0	8	9	7	0	7	8
5104.7937	5104.7863	0.0074	8	0	8	8	7	0	7	7
5104.7937	5104.7754	0.0183	8	0	8	7	7	0	7	6
5209.2163	5209.2141	0.0022	8	2	7	9	7	2	6	8
5209.2163	5209.2067	0.0096	8	2	7	7	7	2	6	6
5246.2483	5246.2568	-0.0085	8	3	6	8	7	3	5	7
5246.3702	5246.3627	0.0075	8	3	6	9	7	3	5	8
5246.3702	5246.3670	0.0032	8	3	6	7	7	3	5	6
5258.8228	5258.8270	-0.0042	8	3	5	8	7	3	4	7
5258.9377	5258.9336	0.0041	8	3	5	9	7	3	4	8
5258.9377	5258.9379	-0.0002	8	3	5	7	7	3	4	6
5334.9297	5334.9145	0.0152	8	2	6	9	7	2	5	8
5334.9297	5334.9071	0.0226	8	2	6	7	7	2	5	6
5343.7955	5343.7831	0.0124	8	1	7	8	7	1	6	7
5343.7955	5343.7854	0.0101	8	1	7	7	7	1	6	6
5718.4414	5718.4427	-0.0013	9	0	9	10	8	0	8	9
5718.4414	5718.4377	0.0037	9	0	9	9	8	0	8	8
5718.4414	5718.4298	0.0116	9	0	9	8	8	0	8	7
5852.9819	5852.9621	0.0198	9	2	8	10	8	2	7	9
5901.5386	5901.5449	-0.0063	9	4	6	10	8	4	5	9
5901.5386	5901.5549	-0.0163	9	4	6	8	8	4	5	7
5902.5177	5902.5057	0.0120	9	4	5	10	8	4	4	9
5902.5177	5902.5157	0.0020	9	4	5	8	8	4	4	7
5903.8469	5903.8618	-0.0149	9	3	7	10	8	3	6	9
5903.8469	5903.8617	-0.0148	9	3	7	8	8	3	6	7
5926.4203	5926.4367	-0.0164	9	3	6	10	8	3	5	9
5926.4203	5926.4367	-0.0164	9	3	6	8	8	3	5	7
5995.8853	5995.8876	-0.0023	9	1	8	10	8	1	7	9
5995.8853	5995.8761	0.0092	9	1	8	8	8	1	7	7
6018.3034	6018.3051	-0.0017	9	2	7	10	8	2	6	9
6018.3034	6018.2977	0.0057	9	2	7	8	8	2	6	7
6275.2558	6275.2593	-0.0035	7	2	5	6	6	1	5	5
6275.4196	6275.4064	0.0132	7	2	5	7	6	1	5	6
6290.8664	6290.8683	-0.0019	10	1	10	11	9	1	9	10
6330.1118	6330.1124	-0.0006	10	0	10	11	9	0	9	10
6330.1118	6330.1078	0.0040	10	0	10	10	9	0	9	9
6330.1118	6330.1021	0.0097	10	0	10	9	9	0	9	8
6413.5147	6413.5224	-0.0077	5	3	2	5	4	2	2	4
6494.2548	6494.2520	0.0028	10	2	9	11	9	2	8	10
6494.2548	6494.2453	0.0095	10	2	9	9	9	2	8	8
6560.9845	6560.9943	-0.0098	10	3	8	11	9	3	7	10
6560.9845	6560.9922	-0.0077	10	3	8	9	9	3	7	8
6562.5751	6562.5712	0.0039	10	4	6	10	9	4	5	9
6562.6585	6562.6673	-0.0088	10	4	6	11	9	4	5	10
6562.6585	6562.6718	-0.0133	10	4	6	9	9	4	5	8
6598.5659	6598.5583	0.0076	10	3	7	11	9	3	6	10
6641.3208	6641.3229	-0.0021	10	1	9	11	9	1	8	10
6641.3208	6641.3143	0.0065	10	1	9	10	9	1	8	9
6641.3208	6641.3134	0.0074	10	1	9	9	9	1	8	8
6700.6694	6700.6703	-0.0009	10	2	8	11	9	2	7	10
6911.0696	6911.0750	-0.0054	11	1	11	12	10	1	10	11

6911.0696	6911.0672	0.0024	11	1	11	11	10	1	10	10
6911.0696	6911.0669	0.0027	11	1	11	10	10	1	10	9
6941.1578	6941.1623	-0.0045	11	0	11	12	10	0	10	11
7217.3489	7217.3421	0.0068	11	3	9	12	10	3	8	11
7217.3489	7217.3392	0.0097	11	3	9	10	10	3	8	9
7224.5422	7224.5550	-0.0128	11	4	7	12	10	4	6	11
7224.5422	7224.5566	-0.0144	11	4	7	10	10	4	6	9
7275.8987	7275.9016	-0.0029	11	3	8	12	10	3	7	11
7275.8987	7275.8987	-0.0000	11	3	8	10	10	3	7	9
7279.2564	7279.2574	-0.0010	11	1	10	12	10	1	9	11
7279.2564	7279.2511	0.0053	11	1	10	11	10	1	9	10
7279.2564	7279.2495	0.0069	11	1	10	10	10	1	9	9
7380.1294	7380.1300	-0.0006	11	2	9	12	10	2	8	11
7380.1294	7380.1106	0.0188	11	2	9	11	10	2	8	10
7380.1294	7380.1238	0.0056	11	2	9	10	10	2	8	9
7530.0505	7530.0545	-0.0040	12	1	12	13	11	1	11	12
7530.0505	7530.0482	0.0023	12	1	12	12	11	1	11	11
7530.0505	7530.0477	0.0028	12	1	12	11	11	1	11	10
7552.4103	7552.4135	-0.0032	12	0	12	13	11	0	11	12
7552.4103	7552.4095	0.0008	12	0	12	12	11	0	11	11
7552.4103	7552.4064	0.0039	12	0	12	11	11	0	11	10
7768.9136	7768.9208	-0.0072	12	2	11	13	11	2	10	12
7768.9136	7768.9049	0.0087	12	2	11	12	11	2	10	11
7768.9136	7768.9154	-0.0018	12	2	11	11	11	2	10	10
7872.4750	7872.4700	0.0050	12	3	10	13	11	3	9	12
7872.4750	7872.4668	0.0082	12	3	10	11	11	3	9	10
7881.0634	7881.0623	0.0011	12	4	9	13	11	4	8	12
7881.0634	7881.0622	0.0012	12	4	9	11	11	4	8	10
7888.5904	7888.5982	-0.0078	12	4	8	13	11	4	7	12
7888.5904	7888.5982	-0.0078	12	4	8	11	11	4	7	10
7909.2837	7909.2931	-0.0094	12	1	11	13	11	1	10	12
7909.2837	7909.2883	-0.0046	12	1	11	12	11	1	10	11
7909.2837	7909.2863	-0.0026	12	1	11	11	11	1	10	10
7958.5999	7958.6069	-0.0070	12	3	9	13	11	3	8	12
7958.5999	7958.6037	-0.0038	12	3	9	11	11	3	8	10

3.4 Clusters with 3 water molecules

Table S59. Predicted spectroscopic constants for the equilibrium anda transition-state tri-hydrated structures. The A,B,C parameters are the rotational constants, and $\chi_{aa}, \chi_{bb} - \chi_{cc}$, the nuclear quadrupole coupling constants associated to the ^{14}N atom. The predicted dipole moment components, μ, for the a-, b-, and c-type transitions. The standard error of each fit, σ, and the predicted zero-point corrected relative energy, ΔE_{ZPC} are also shown. All theoretical parameters are expressed in the *principal axis system* (PAS).

Constants	$\mathscr{O}_{3w}$ (M$_1$)	$\mathscr{O}_{3w}$ (M$_2$)	$\mathscr{O}_{3w}$ (M$_3$)	$\mathscr{O}_{3w}$ (M$_4$)	$\mathscr{O}_{3w}$ (M$_5$)	$\mathscr{O}_{3w}$ (M$_6$)
A /MHz	842	841	831	841	823	838
B /MHz	264	264	267	267	271	270
C /MHz	233	233	238	237	243	240
χ_{aa}	-4.0	-4.0	-4.0	-4.0	-4.0	-3.9
$\chi_{bb} - \chi_{cc}$ /MHz	-1.9	-2.1	-2.1	-2.0	-1.9	-1.7
μ_a /D	-2.8	2.8	-3.4	-3.3	-2.3	-2.4
μ_b /D	0.9	1.0	-0.8	0.7	-0.1	0.1
μ_c /D	-0.3	0.5	-0.6	0.9	1.0	0.8
ΔE_{ZPC} /kJ mol^{-1}	0	+0.2	+0.2	+0.3	+2.0	+2.0

Constants	$\mathscr{O}_{3w}$ (TS$_1$)	$\mathscr{O}_{3w}$ (TS$_2$)	$\mathscr{O}_{3w}$ (TS$_3$)	$\mathscr{O}_{3w}$ (TS$_4$)	$\mathscr{O}_{3w}$ (TS$_5$)
A /MHz	817	832	841	845	830
B /MHz	272	271	263	263	272
C /MHz	242	240	233	232	241
χ_{aa}	-4.0	-3.9	-4.1	-4.1	-3.9
$\chi_{bb} - \chi_{cc}$ /MHz	-2.0	-1.9	-1.9	-1.9	-1.8
μ_a /D	-2.4	-2.4	-2.2	-2.3	-2.4
μ_b /D	-0.1	-0.1	-1.5	1.5	0.1
μ_c /D	-0.1	0.3	1.1	0.9	-0.3
ΔE_{ZPC} /kJ mol^{-1}	+0.9	+1.1	+1.3	+1.3	+1.4

Table S60. Kraitchman Analysis for $\mathscr{O}_{3w}$. The experimental coordinates obtained through the Kraitchman equations, a, b, c are represented in their absolute value. The theoretical coordinates predicted for the different energy minima (M) are also presented as well as the corresponding absolute deviation from the experimental value. Theoretical coordinates are presented in the Principal Axis System.

	Coordinate	Experimental Kraitchman	M_1 Value	M_1 Deviation	M_2 Value	M_2 Deviation	M_3 Value	M_3 Deviation	M_4 Value	M_4 Deviation
O(29)	|a| /Å	5.3402(5)	5.3463	0.0061	5.3414	0.0013	5.1942	0.1459	5.2354	0.1048
	|b| /Å	0.776(4)	-0.782	0.006	-0.820	0.044	-0.805	0.029	-0.811	0.035
	|c| /Å	0.19(2)	-0.229	0.041	0.046	0.143	-0.665	0.477	0.439	0.250
O(32)	|a| /Å	2.8713(9)	2.9121	0.0407	2.8739	0.0026	2.9349	0.0636	2.9101	0.0388
	|b| /Å	1.861(2)	-1.9008	0.0397	-1.9114	0.0503	-1.8691	0.0080	-1.8514	0.0097
	|c| /Å	0.320(9)	0.268	0.052	-0.331	0.011	0.434	0.114	-0.535	0.215
O(35)	|a| /Å	4.2528(9)	4.2520	0.0008	4.2833	0.0305	4.2756	0.0228	4.2684	0.0156
	|b| /Å	1.686(2)	1.7368	0.0509	1.7157	0.0298	1.6705	0.0155	1.6791	0.0068
	|c| /Å	0.21(2)	-0.191	0.017	0.016	0.192	0.093	0.114	-0.209	0.001

	Coordinate	M_5 Value	M_5 Deviation	M_6 Value	M_6 Deviation
O(29)	a /Å	4.9880	0.3521	5.0676	0.2726
	b /Å	-0.801	0.024	-0.786	0.010
	c /Å	-0.938	0.749	0.731	0.543
O(32)	a /Å	2.9392	0.0678	2.9283	0.0569
	b /Å	-1.8431	0.0179	-1.8244	0.0367
	c /Å	0.536	0.216	-0.610	0.291
O(35)	a /Å	4.2817	0.0289	4.2671	0.0143
	b /Å	1.6246	0.0613	1.6388	0.0471
	c /Å	0.180	0.028	-0.322	0.114

3.4.1 Equilibrium and transition state geometries

Table S61. Equilibrium geometry of $\mathcal{O}_{3w}$ (M_1).

Atom	x /Å	y /Å	z /Å
C	-1.66725173627127	1.19981495969125	1.59534083408737
C	-2.30186109295467	-0.21964792816533	1.64292622761886
C	-2.18547283553024	-0.75493470485831	0.18349335081248
C	-0.69732015285269	-1.04303553191260	0.02930396528785
C	-0.06290890547683	0.31994755034263	0.02060715591892
C	-1.18922854918120	1.30872575755611	0.12646062124583
C	-3.08458988125205	-1.92554160459972	-0.14537045920613
C	-2.30464608070748	0.55763076987758	-0.65516751396792
C	-1.96147255735527	0.36398372663165	-2.13530373227867
C	-3.67440640746591	1.22928693421939	-0.56142180992054
O	-0.15281326119543	-2.12580262354430	-0.05912170974542
N	1.21066893619052	0.46924709036459	-0.03557887800972
O	1.59764085796305	1.78591841993877	-0.00925529029765
H	-2.39794383815846	1.97793788798685	1.82504967805347
H	-0.83533265942780	1.31369705410928	2.29355161275677
H	-1.79464458522709	-0.88451174607660	2.34800259844859
H	-3.35469971800096	-0.18653855738572	1.93464117739838
H	-0.94455510999312	2.31459499290748	-0.21509056228538
H	-4.13585710726246	-1.66010194341676	-0.00545453103545
H	-2.94247400362553	-2.25849428002183	-1.17660505633959
H	-2.85606236347611	-2.77311719578683	0.50587537232708
H	-0.99186059000626	-0.11816713228347	-2.28963015082148
H	-2.72121610817244	-0.24787058289337	-2.62994116715101
H	-1.93229227732922	1.33287376344835	-2.64328480673904
H	-4.42228400946447	0.64137035442414	-1.10128256203400
H	-4.02726029678112	1.35716481112379	0.46235517949418
H	-3.63408385331143	2.21908143548181	-1.02739285990242
H	2.58048858384551	1.74077078064082	-0.13001466203231
O	5.34092191969795	-0.83970575304122	-0.26683201432685
H	5.94825221201422	-1.06114240325002	0.44652381230797
H	4.54171266139139	-1.38538312854726	-0.10369066077059
O	4.25990259279967	1.67979556291093	-0.43159856545433
H	4.54443730336737	2.01912041712025	-1.28667491669107
H	4.70305776623786	0.80625655330068	-0.34313495698425
O	2.90803734586092	-1.89670908609950	0.35529258692565
H	2.32057546185221	-2.61830510098330	0.10286010719431
H	2.34777650735894	-1.10566439411022	0.23659941371613

Table S62. Equilibrium geometry of $\mathscr{O}_{3w}$ (M_2).

Atom	x /Å	y /Å	z /Å
C	-1.68849871482691	1.29781599411125	1.60420556471629
C	-2.32652378413413	-0.11519849688323	1.73088600397149
C	-2.19259833754097	-0.73868535195461	0.30834413473687
C	-0.70232727522917	-1.03601061270392	0.19350708744111
C	-0.06719146500476	0.32384991130830	0.09917760456074
C	-1.19407707160338	1.31716885188585	0.13712494922417
C	-3.08800275886028	-1.92791959394565	0.04186187716818
C	-2.30101585420713	0.51950208514432	-0.61033796255022
C	-1.94067395673268	0.23483558040624	-2.07166447966503
C	-3.67093377946288	1.19641204725372	-0.57410942797891
O	-0.15663504107183	-2.12164460569969	0.20111574574763
N	1.20675613660189	0.47013245730117	0.04650497485528
O	1.59282051045160	1.78665678583427	-0.00102577981389
H	-2.42033640809553	2.08924145529105	1.77823364565867
H	-0.86423247418578	1.45281393356557	2.30366099042879
H	-1.82976972692990	-0.73645501348754	2.48171435150007
H	-3.38295807890310	-0.06269300126817	2.00633008410593
H	-0.94552533147951	2.30029836103007	-0.26289770559548
H	-2.93770431908757	-2.32060281520649	-0.96697612757574
H	-2.86377089653712	-2.73541131273497	0.74360145704892
H	-4.14056218299736	-1.65597110023960	0.15770954009591
H	-2.69731536112412	-0.40319389276400	-2.53713066554084
H	-1.90090116178954	1.17072744231950	-2.63741358098792
H	-0.97154334551429	-0.25953252741260	-2.18498789928791
H	-4.03501095004184	1.38867970619795	0.43562805090079
H	-3.62430985089322	2.15482217935009	-1.10105893765389
H	-4.41348768963350	0.57597583239536	-1.08412586737694
H	2.58258580475438	1.73890458287968	0.00611003954916
O	5.33291788122984	-0.85640672078171	-0.07797496505908
H	4.51807733025303	-1.39651054796125	-0.16469574930980
H	5.86156602370875	-1.07494587075395	-0.85250243337625
O	4.29033869976255	1.68068017363385	0.08283729270561
H	4.71536161678121	0.79602988224413	0.01507025000643
H	4.69468653569693	2.09630868279659	0.85153588493301
O	2.85286354175019	-1.90699030651633	-0.48692232296177
H	2.28184376313231	-2.62934690379930	-0.20130578607416
H	2.30545558276384	-1.11524511493594	-0.32290793284719

Table S63. Equilibrium geometry of $\mathscr{O}_{3w}$ (M_3).

Atom	x /Å	y /Å	z /Å
C	-1.64726382951178	1.42298698871451	1.55594133040391
C	-2.23639076429037	0.01167489321247	1.84032870117005
C	-2.14977063849866	-0.73640610977268	0.47538800751677
C	-0.65935969546323	-1.00758680114595	0.31322368663058
C	-0.06658259168020	0.35320183096898	0.07709838387703
C	-1.21822522226164	1.31863333708680	0.07179248624331
C	-3.02342011875824	-1.96529999377128	0.35981646236505
C	-2.33449279108722	0.42993703059957	-0.54736588267462
C	-2.03202236884261	0.02231395527330	-1.99255292014552
C	-3.72030275853796	1.07345613284341	-0.50781687827543
O	-0.08729024033980	-2.07851596185021	0.36930595399220
N	1.19949781331664	0.52634118279708	-0.04523688925092
O	1.54460860123559	1.84042207732224	-0.23601361919597
H	-2.39390686494735	2.20815646901411	1.69090535415860
H	-0.79811148490761	1.66291425849578	2.19936708606690
H	-1.68734581026081	-0.52510809850190	2.61935911856717
H	-3.28005536164926	0.06202512980615	2.16105436629474
H	-1.01357994187634	2.26609815335551	-0.42685534796147
H	-2.74889161235866	-2.69828483898130	1.12279468839121
H	-4.07710119295430	-1.70768782486920	0.49607072722210
H	-2.90465204273171	-2.44727594000979	-0.61389715580984
H	-2.78793121209587	-0.67659348323591	-2.36184339327348
H	-2.04787237931259	0.90318687147777	-2.64167713866074
H	-1.05344119465550	-0.45286754599655	-2.10657990402938
H	-3.72401486146262	1.98206477591021	-1.11841565394761
H	-4.46645914121735	0.39211218249095	-0.92665724882975
H	-4.04524621386601	1.34581480925776	0.49679777655817
H	2.52840451902113	1.80525784952850	-0.35316180946563
O	2.98357775849219	-1.79531015041993	0.21586733340724
H	2.34277304813015	-2.51568249104830	0.23105476098182
H	2.41679030652871	-1.00098374096934	0.18209961417998
O	4.21211758904349	1.71280530779922	-0.57535904615653
H	4.57368232160927	0.84255907611696	-0.85612285224706
H	4.79462770354165	2.01526529101562	0.12892782768443
O	5.08174422360934	-0.81114254652969	-1.22448924762890
H	4.41053540176570	-1.35491468977378	-0.75993928671960
H	5.07743066627389	-1.11604174201106	-2.13723571473881

Table S64. Equilibrium geometry of $\mathscr{O}_{3w}$ (M_4).

Atom	x /Å	y /Å	z /Å
C	-1.61921484036000	1.23941352769969	1.61430685788099
C	-2.24835748392086	-0.17943801569965	1.71793635133041
C	-2.17653995605344	-0.75106909033729	0.26925502043155
C	-0.69245755032751	-1.03897798311170	0.07791451197844
C	-0.06447490656124	0.32517974018231	0.00573738003404
C	-1.19032100930214	1.31283273261901	0.12841808736291
C	-3.08089280350656	-1.93285471474430	-0.00006934586764
C	-2.32791323105160	0.53905707699266	-0.59761609385060
C	-2.03256086132965	0.30829180135208	-2.08280222564986
C	-3.69613878507201	1.20945802786254	-0.47630997787146
O	-0.14638253003623	-2.12324391364220	0.02087854731377
N	1.20573519155885	0.47853148121916	-0.09706178515492
O	1.58756813220697	1.79649999247559	-0.10866978797931
H	-2.34405122393729	2.02154555708759	1.84892028838862
H	-0.76494476329804	1.37242186201879	2.28150456157537
H	-1.71781323298152	-0.82564195836803	2.42324272394673
H	-3.29166762315909	-0.14067385187693	2.04144677680533
H	-0.96099474003002	2.31051182629823	-0.24621290928104
H	-2.97383905585346	-2.28945339419659	-1.02764499164604
H	-2.82549086908081	-2.76386833882621	0.66245572161450
H	-4.12781212455633	-1.66821513788094	0.17065011653823
H	-1.06866443086412	-0.17866104408154	-2.25669192100773
H	-2.80809313651442	-0.31496822482132	-2.53703064970674
H	-2.01962612358994	1.26409650236281	-2.61558334647549
H	-4.01500510690560	1.36471435402722	0.55483276278703
H	-3.67489829816353	2.18608129658601	-0.97042272344786
H	-4.45961204501406	0.60492731978279	-0.97438733225039
H	2.57564393141219	1.74811179353486	-0.17405700145088
O	5.21859686192559	-0.86160458865554	0.44308628228004
H	5.41250416540975	-1.11779537300937	1.35019291802418
H	4.46585952964953	-1.42398680883662	0.16114111254030
O	4.27182468751648	1.62779909580053	-0.23599498108384
H	4.71955606022532	1.82757147969941	-1.06467960244041
H	4.65858529419651	0.77559303795052	0.06506853084196
O	2.88438769067352	-1.89520930685977	-0.51616350467079
H	2.35069561035883	-1.08526450255042	-0.40240417546412
H	2.24497263343606	-2.59702193675331	-0.34658087647530

Table S65. Equilibrium geometry of $\mathscr{O}_{3w}$ (M_5).

Atom	x /Å	y /Å	z /Å
C	-1.67558207356627	1.40801756494286	1.54734276858255
C	-2.29248754492868	0.00111976088793	1.79199610725033
C	-2.15169227491908	-0.73647867746239	0.42597773260870
C	-0.65897735530633	-1.02383247785620	0.32845469937648
C	-0.04031961143353	0.33261080901804	0.13350143171171
C	-1.17963904425286	1.31103883802131	0.08378069508484
C	-3.03327468003998	-1.95376466806878	0.25926796527598
C	-2.27587261278704	0.44042479512682	-0.59414039458506
C	-1.91111306372245	0.04158070215912	-2.02733646329522
C	-3.65479905084420	1.09943686297892	-0.61266578808557
O	-0.10334724755259	-2.10271034839453	0.39550462783795
N	1.23155809340452	0.49137315671606	0.06980019145322
O	1.60041756583902	1.80375027009941	-0.09524080628050
H	-2.41932704665775	2.20023804860772	1.65409829949247
H	-0.85478840582557	1.63354908389789	2.23150236856910
H	-1.78543253557379	-0.54829079032910	2.59046948623620
H	-3.34909417345665	0.06066182048444	2.06518544168601
H	-0.94173494305437	2.26009852176266	-0.39671228092002
H	-2.87252059066265	-2.43103239899514	-0.71068713338519
H	-2.80564874531719	-2.69492684821529	1.02975930592313
H	-4.08913460342152	-1.68379839298210	0.34546090102578
H	-2.65617152447578	-0.64642895767288	-2.43692284589333
H	-1.88844617649969	0.92792556136397	-2.66867070887557
H	-0.93302224501404	-0.44244097205650	-2.10105240817517
H	-4.02303706490135	1.36675328354843	0.37828982828038
H	-3.62002069511551	2.01321401547257	-1.21446258381315
H	-4.38812159637181	0.43036471683007	-1.07195699555075
H	2.58615571124533	1.75034498758955	-0.18297119469079
O	2.96091373080394	-1.87248831414139	0.29297709847211
H	2.26958759242442	-2.54512516731618	0.25602180874009
H	2.44169118257772	-1.04489713080731	0.29670405169130
O	4.27741762292722	1.56815513048755	-0.32605357916956
H	4.55581151175363	0.74612136313080	-0.78616187663956
H	4.76329032405785	1.56374548543273	0.50551791867753
O	4.85704584983030	-0.90274655743622	-1.41605498297503
H	5.70032263701076	-1.36441218337120	-1.37971789603628
H	4.22795830272596	-1.44589362905365	-0.89492365600506

Table S66. Equilibrium geometry of $\mathscr{O}_{3w}$ (M_6).

Atom	x /Å	y /Å	z /Å
C	-1.54952216583169	1.18902526599005	1.66222528294991
C	-2.17841574508107	-0.23118697720439	1.74644095675696
C	-2.15723917228001	-0.76105081079029	0.28023169058931
C	-0.68105778263297	-1.04472426213954	0.03109009967335
C	-0.05343645961089	0.32026780559343	-0.02421986515671
C	-1.17231586526152	1.30508323570965	0.16493289999565
C	-3.07244115428256	-1.93380924888962	0.00833771804677
C	-2.33577730609897	0.55357830303471	-0.54335678485318
C	-2.09228890407821	0.36492788573348	-2.04382782265967
C	-3.69740594021894	1.22200427024934	-0.35592801668671
O	-0.13936801759798	-2.12786963419982	-0.07155478354425
N	1.21332809352085	0.47486134116588	-0.15932746789457
O	1.59782499407062	1.79285980468316	-0.14132463910059
H	-2.26356764647551	1.96521924457039	1.94467930152180
H	-0.67211466072346	1.30081841863171	2.30265980590360
H	-1.62591524155802	-0.89810442146230	2.41452049985486
H	-3.21015992284378	-0.20007583135076	2.10583121481702
H	-0.95421026656435	2.31297417943379	-0.18834833924286
H	-2.99647128713741	-2.26510160038542	-1.03043063881718
H	-2.80034188020419	-2.78145668156585	0.64249747228875
H	-4.11312000669909	-1.67115407687217	0.21617190438839
H	-2.09384276954146	1.33559465040149	-2.54919531024739
H	-1.13677199856147	-0.11972516920609	-2.26423749361551
H	-2.88521256544397	-0.24291377495836	-2.48884350022072
H	-3.69135809161828	2.21152406563773	-0.82411450015672
H	-4.47938149461407	0.63212908131434	-0.84273115443407
H	-3.97916688034399	1.35002513680307	0.68962772510547
H	2.58466813053985	1.73917769710210	-0.21579050750342
O	5.04350908369497	-0.84130010185402	0.80602711008727
H	5.87008560362448	-1.31305101273947	0.66533267897115
H	4.33900119988629	-1.41149086306083	0.43060197121657
O	4.27757209741954	1.55505761404124	-0.33519687357722
H	4.63731243236535	0.76116025926835	0.11791370840545
H	4.62181595204139	1.50634452225338	-1.23339416621347
O	2.90005501894245	-1.90041281776339	-0.51232928835496
H	2.22801679359801	-2.56811215339115	-0.32716349679984
H	2.38320595460013	-1.07209671998376	-0.47361341369329

Table S67. Transition-state geometry of $\mathscr{O}_{3w}$ (TS_1).

Atom	x /Å	y /Å	z /Å
C	-1.30044334014028	1.40253773112602	1.61112701964956
C	-1.92408776067237	-0.00265506349250	1.84843645629761
C	-1.77153500796204	-0.73934852690935	0.48315603726675
C	-0.27848456107787	-1.03086954371182	0.40142008422176
C	0.34603507989379	0.32346380980938	0.21211122954900
C	-0.79045859323396	1.30509115171974	0.15249849615634
C	-2.65438111213665	-1.95445263659051	0.30685286907689
C	-1.88242040572777	0.43852768019277	-0.53692041081353
C	-1.50492450637504	0.03974209627229	-1.96684043232618
C	-3.25923009820152	1.10157532822072	-0.56854341240479
O	0.27254323061736	-2.11167603257359	0.47700618889731
N	1.61894809325676	0.48114429789947	0.15683350219384
O	1.98678838308959	1.79371768323879	-0.00482883944745
H	-2.04268020822322	2.19704601331713	1.71146119585600
H	-0.48556768973071	1.62478759586832	2.30339003700993
H	-1.42685967821004	-0.55436411537888	2.65149332505194
H	-2.98326726469893	0.05974981687631	2.11093992408009
H	-0.54505035731263	2.25385211446367	-0.32481803207865
H	-2.48672318430997	-2.43028568609909	-0.66267338274202
H	-2.43471240095507	-2.69753420961576	1.07779764504369
H	-3.71036684926479	-1.68237380405609	0.38475640997438
H	-2.24824439924800	-0.64535253900333	-2.38446610231680
H	-1.47281612726165	0.92655280841407	-2.60717646443553
H	-0.52776247645623	-0.44751671764484	-2.03103657953383
H	-3.63637522942340	1.36911920647026	0.41899667057725
H	-3.21596168645895	2.01580260015057	-1.16913686788708
H	-3.99002482138311	0.43502837703708	-1.03554168159135
H	2.97292273883888	1.74722959492844	-0.09197234908637
O	5.26285508384654	-0.88117427150938	-1.20389947019836
H	5.85834437526908	-1.44279164564445	-1.70617289775036
H	4.64521275604488	-1.46506526119304	-0.71695691304594
O	3.32999433900399	-1.91022769754986	0.41712794458014
H	2.63738179097509	-2.58047062315285	0.36798720652538
H	2.81322978488448	-1.08175283463320	0.41402311721094
O	4.66269353452061	1.63247561886607	-0.28570060383970
H	4.95921894120257	0.80392681507966	-0.71659086172629
H	5.20620962702068	1.70654086880775	0.50585994200545

Table S68. Transition-state geometry of $\mathscr{O}_{3w}$ (TS_2).

Atom	x /Å	y /Å	z /Å
C	-1.05031953579929	1.27353086976243	1.66120390234042
C	-1.63469861038353	-0.14698597454127	1.90676775354197
C	-1.71758659231111	-0.79100392573149	0.48939824685801
C	-0.25938234564018	-1.06608309631721	0.14259989804677
C	0.32750430138937	0.30135049394253	-0.06985827343390
C	-0.79749182586814	1.27688408566049	0.13365086583677
C	-2.62145226293413	-1.99900721745200	0.38507846243029
C	-1.99474110278122	0.45024807048339	-0.41639566113340
C	-1.86796587944023	0.14754536024475	-1.91258808022053
C	-3.35344094635755	1.10569829412636	-0.17042050106261
O	0.29793306502361	-2.14455065008989	0.08062455315271
N	1.57509390627091	0.47209338026211	-0.32030163110172
O	1.91919606705217	1.79584565329812	-0.43464257270762
H	-1.75851313335764	2.05634863408788	1.94020511925160
H	-0.12727187308939	1.45223136791043	2.21672251680530
H	-1.01398202907117	-0.74840309264709	2.57718689638645
H	-2.63482314262997	-0.10678498321729	2.34589077024147
H	-0.63352793249798	2.25736252259477	-0.31356388033790
H	-2.62161349352123	-2.40909506789746	-0.62803231328983
H	-2.27698054345695	-2.78900948231164	1.05735210462127
H	-3.64824145141384	-1.74106091469429	0.65786968923460
H	-0.92197172098838	-0.33629561931728	-2.17229782976579
H	-2.67939146200484	-0.50732066704188	-2.24276483303381
H	-1.93313372935072	1.07493211795900	-2.48991626096622
H	-3.55286961276605	1.31250806528481	0.88145887383266
H	-3.41069115116523	2.05402277850170	-0.71428262143493
H	-4.15694279601697	0.46359812574550	-0.54264805493920
H	2.89853443617327	1.76661475023100	-0.58387829702754
O	5.49065044478924	-0.66587897164654	0.28389753290669
H	6.21210010146117	-1.14652735958197	0.69716181561593
H	4.79776762086496	-1.31688006278402	0.04822924778079
O	4.58743457379138	1.68671124256537	-0.79905299134443
H	4.91726238298165	1.65006724317018	-1.70312474067821
H	5.01283697417873	0.92944219617773	-0.34547594182675
O	3.26535229623288	-1.92410184303834	-0.65756137869780
H	2.73451926112496	-1.10481705566406	-0.63802713376768
H	2.63084774151145	-2.58322926803482	-0.35046525211387

Table S69. Transition-state geometry of $\mathscr{O}_{3w}$ (TS$_3$).

Atom	x /Å	y /Å	z /Å
C	-1.23876729578328	1.31062190040845	1.65622441137691
C	-1.87101768396813	-0.09806884795657	1.84546441914880
C	-1.80499650678118	-0.75491130670573	0.43336567561501
C	-0.32330416924892	-1.05990662786646	0.25218927664056
C	0.31096904285096	0.29564912517974	0.10721003717309
C	-0.81109507248157	1.29327719747760	0.16819796592557
C	-2.71525184783501	-1.94624022465430	0.23569886442435
C	-1.95270764391080	0.48237331326384	-0.50881076726829
C	-1.66031932770694	0.16364533001132	-1.97818337140556
C	-3.31844912611342	1.16355573394583	-0.42531107085551
O	0.21585188770803	-2.14879856098708	0.23386477857543
N	1.58114711682312	0.43708923895003	-0.01106433073630
O	1.96561216236086	1.75099150805856	-0.11486464226152
H	-1.96157445344855	2.10724130137291	1.84380482455870
H	-0.38373351632918	1.48094871876033	2.31391463889361
H	-1.33948653161715	-0.70238546589786	2.58625457229028
H	-2.91313003838049	-0.03759742107095	2.16953929592012
H	-0.57765113270441	2.26552731697995	-0.26602339538388
H	-3.76109573234488	-1.66600760244711	0.38715983678084
H	-2.60774066766340	-2.36645158068544	-0.76747240831627
H	-2.46586516615099	-2.73600351453053	0.94904205164613
H	-0.69602045461234	-0.33132422137762	-2.12476606071372
H	-2.43608114522835	-0.48636548738499	-2.39284235629521
H	-1.64943209962453	1.08604987280487	-2.56689621433904
H	-4.08427135921159	0.53457130172770	-0.88825196577044
H	-3.63638439893566	1.37789082934897	0.59549034647458
H	-3.29389812228353	2.11055337793746	-0.97395648065391
H	2.93827120143675	1.69078877076974	-0.27866684328644
O	5.66103843208535	-1.03598572719654	-0.69951915413915
H	6.34247837447051	-1.30515333104053	-0.07496912532636
H	4.85649324814849	-1.52192915751735	-0.41624986236716
O	4.59950973798571	1.50951991096902	-0.66660065399288
H	5.28307370813066	2.18510014028681	-0.65228539067426
H	5.06051940606264	0.64307525186823	-0.69558559029505
O	3.29172231395322	-1.93479014282284	0.31383606729144
H	2.64361774227874	-2.63285089150747	0.16247620466316
H	2.75196911806929	-1.12370002847202	0.24858641668229

Table S70. Transition-state geometry of $\mathscr{O}_{3w}$ (TS_4).

Atom	x /Å	y /Å	z /Å
C	-2.05469774738028	-1.09884060643016	0.70830455034839
C	-2.17392773927620	-1.19616851458737	-0.83962058347082
C	-1.45551768651048	0.08053894885019	-1.37272082939033
C	0.01788099173920	-0.21138878594063	-1.11477642063038
C	0.14339665447186	-0.14914747746275	0.38223248097380
C	-1.22357435304629	0.19274764880691	0.90430844960068
C	-1.77671644006157	0.44736592990426	-2.80427488353716
C	-1.74199504758070	1.11352784049134	-0.23728791474096
C	-0.92751443774772	2.40364706676758	-0.37348328660050
C	-3.21749114878325	1.48702060995051	-0.09739746674332
O	0.88089463934527	-0.46161938404398	-1.93285845578524
N	1.25004215563672	-0.40948634119197	0.97785677758630
O	1.15026689366363	-0.33933655852649	2.34547410783265
H	-3.03300350129525	-1.00784714145684	1.18461533718118
H	-1.55402894708358	-1.96391073151537	1.14824489107815
H	-1.71455092244441	-2.10374726618162	-1.24170044245341
H	-3.21585240223551	-1.19089393196936	-1.16986412095442
H	-1.23327847652985	0.61000959078928	1.91134071192692
H	-1.23082591615942	1.34083544727883	-3.11777599021755
H	-1.48841102942858	-0.36449875625370	-3.47695337408931
H	-2.84741293598362	0.63055463854288	-2.92805937787757
H	-1.26332860146941	2.98088112875724	-1.23968202789981
H	-1.06240537661443	3.02818390622065	0.51498677533150
H	0.14501420782965	2.22233908745272	-0.48867569493172
H	-3.88202096996868	0.62398133435627	-0.04653552288945
H	-3.36522893318008	2.07693249123608	0.81288720502697
H	-3.53437176882867	2.10294653408675	-0.94396042340573
H	2.04752152455805	-0.61658175174796	2.65301539811437
O	5.43796132723010	-1.73272754535266	1.13618558737915
H	4.89530400096402	-1.45711600222623	0.36606571072601
H	6.24989019883879	-1.22114153966120	1.05904202825377
O	3.59123813898769	-1.20558680270643	3.11667619039422
H	4.30905600065004	-1.41187518749295	2.47927745681769
H	3.99954550918558	-1.15905659547772	3.98558608584957
O	3.68421597504310	-0.65542912556628	-0.65643626526699
H	3.29390625385183	-0.84555358189887	-1.51766756164516
H	2.90001990961242	-0.53955857580009	-0.08636910189196

Table S71. Transition-state geometry of $\mathscr{O}_{3w}$ (TS_5).

Atom	x /Å	y /Å	z /Å
C	-1.15834509318957	1.19113601804938	1.61851265166345
C	-1.77212246037360	-0.23391427439434	1.72792449476956
C	-1.75754106782745	-0.78481897336195	0.26886529169902
C	-0.28132539805164	-1.06447541605655	0.01550382385528
C	0.33935091307289	0.30291708667370	-0.05683736824790
C	-0.78563081598181	1.28424075494324	0.11908176724509
C	-2.66776677084010	-1.96673691388049	0.02066101827178
C	-1.94681144936573	0.51602237088963	-0.57352889365807
C	-1.70553750440296	0.30872421325934	-2.07194950038602
C	-3.31220362702913	1.17813339715501	-0.39062957182903
O	0.26288791889990	-2.14734465361986	-0.07821222508242
N	1.60634372449192	0.46726713461743	-0.18431496023918
O	1.97556187308954	1.78929225494905	-0.17570001502689
H	-1.88051939105775	1.96449252383570	1.88801720520656
H	-0.28111319591815	1.32380185820881	2.25515935628146
H	-1.20687347223779	-0.88544111679614	2.40073515996691
H	-2.80092065952134	-0.20865013283410	2.09622538208889
H	-0.57398619140045	2.28738984302968	-0.25128293811070
H	-2.62528965935077	-2.28902758589689	-1.02303681749664
H	-2.36163190288329	-2.81567655285854	0.63774809626009
H	-3.70360696666653	-1.71874985821807	0.26795375952685
H	-2.50273046457103	-0.29824841094486	-2.51048637121869
H	-1.70042914576650	1.27390398948468	-2.58786036237797
H	-0.75350029250950	-0.18488293095470	-2.28744821078355
H	-3.60038681825007	1.29809318280503	0.65395989769421
H	-3.30790629438735	2.17042920992362	-0.85250976547679
H	-4.08886369070130	0.58764969794467	-0.88455828541934
H	2.96427195171485	1.75119387065565	-0.23479680995003
O	5.39701194764609	-0.80047658075850	0.79702194398504
H	6.07989351155645	-1.33218896900777	1.21336054858287
H	4.72930776263941	-1.41478904026315	0.42737368360423
O	3.27532590050888	-1.95298664562411	-0.47063438451909
H	2.58744687825551	-2.59201903108908	-0.24590539894197
H	2.77811961104765	-1.11289662173596	-0.46460846413442
O	4.66735149123350	1.63983504117374	-0.22483560867724
H	5.00747848974422	0.84929337157891	0.24365342477626
H	5.10469035838295	1.61950788911788	-1.08262155390165

3.4.2 Transition frequencies

Table S72. Transition frequencies for $\mathscr{O}_{3w}$. J is the rotational angular momentum quantum number, K_a and K_c are the projections of J onto the principal axes at the prolate and oblate symmetric top limits, and F is the total angular momentum quantum number, which includes the nuclear spin, I(^{14}N) = 1.

Observed Frequency /MHz	Calculated Frequency /MHz	Difference / MHz	J	K_a	K_c	F	J'	K'_a	K'_c	F'
2406.0296	2406.0394	-0.0098	5	1	5	6	4	1	4	5
2556.1716	2556.1666	0.0050	5	1	4	6	4	1	3	5
2945.1114	2945.0956	0.0158	6	0	6	7	5	0	5	6
2978.0478	2978.0672	-0.0194	6	2	5	6	5	2	4	5
2978.2159	2978.1952	0.0207	6	2	5	5	5	2	4	4
2988.8930	2988.8905	0.0025	6	3	4	7	5	3	3	6
2990.1180	2990.1068	0.0112	6	3	3	6	5	3	2	5
2990.3906	2990.3895	0.0011	6	3	3	7	5	3	2	6
3016.2419	3016.2275	0.0144	6	2	4	6	5	2	3	5
3016.3720	3016.3691	0.0029	6	2	4	7	5	2	3	6
3016.3720	3016.3695	0.0025	6	2	4	5	5	2	3	4
3063.7844	3063.7864	-0.0020	6	1	5	6	5	1	4	5
3421.4407	3421.4283	0.0124	7	0	7	7	6	0	6	6
3488.1958	3488.2022	-0.0064	7	3	5	7	6	3	4	6
3488.4042	3488.3826	0.0216	7	3	5	8	6	3	4	7
3491.5548	3491.5530	0.0018	7	3	4	7	6	3	3	6
3491.7145	3491.7354	-0.0209	7	3	4	8	6	3	3	7
3530.5804	3530.5839	-0.0035	7	2	5	7	6	2	4	6
3530.6782	3530.6793	-0.0011	7	2	5	8	6	2	4	7
3569.2754	3569.2948	-0.0194	7	1	6	8	6	1	5	7
3837.5325	3837.5524	-0.0199	8	1	8	9	7	1	7	8
3893.2030	3893.2047	-0.0017	8	0	8	9	7	0	7	8
3963.8391	3963.8519	-0.0128	8	2	7	9	7	2	6	8
3988.0720	3988.0788	-0.0068	8	3	6	8	7	3	5	7
3988.1961	3988.2017	-0.0056	8	3	6	9	7	3	5	8
3988.1961	3988.2067	-0.0106	8	3	6	7	7	3	5	6
3994.7207	3994.7201	0.0006	8	3	5	8	7	3	4	7
3994.8463	3994.8459	0.0004	8	3	5	9	7	3	4	8
4048.1620	4048.1709	-0.0089	8	2	6	9	7	2	5	8
4071.9992	4071.9914	0.0078	8	1	7	7	7	1	6	6
4312.1096	4312.1153	-0.0057	9	1	9	10	8	1	8	9
4361.3714	4361.3731	-0.0017	9	0	9	10	8	0	8	9
4454.5730	4454.5661	0.0069	9	2	8	10	8	2	7	9
4485.7101	4485.7002	0.0099	9	4	6	10	8	4	5	9
4486.1195	4486.1144	0.0051	9	4	5	10	8	4	4	9
4488.0986	4488.1024	-0.0038	9	3	7	9	8	3	6	8
4488.1984	4488.1900	0.0084	9	3	7	10	8	3	6	9
4500.1160	4500.1145	0.0015	9	3	6	9	8	3	5	8
4500.2160	4500.2062	0.0098	9	3	6	10	8	3	5	9
4567.6880	4567.6778	0.0102	9	2	7	10	8	2	6	9
4571.3297	4571.3336	-0.0039	9	1	8	10	8	1	7	9
4785.4186	4785.4301	-0.0115	10	1	10	11	9	1	9	10
4827.1023	4827.0912	0.0111	10	0	10	9	9	0	9	8
4943.6824	4943.6686	0.0138	10	2	9	11	9	2	8	10
4986.2048	4986.1969	0.0079	10	4	7	10	9	4	6	9
4986.3100	4986.3085	0.0015	10	4	7	11	9	4	6	10
4987.2154	4987.2009	0.0145	10	4	6	11	9	4	5	10

4987.2154	4987.2062	0.0092	10	4	6	9	9	4	5	8
4988.1234	4988.1424	-0.0190	10	3	8	11	9	3	7	10
5008.3327	5008.3442	-0.0115	10	3	7	11	9	3	6	10
5008.3327	5008.3422	-0.0095	10	3	7	9	9	3	6	8
5066.5904	5066.5886	0.0018	10	1	9	11	9	1	8	10
5087.7571	5087.7635	-0.0064	10	2	8	9	9	2	7	8
5257.5954	5257.5956	-0.0002	11	1	11	12	10	1	10	11
5291.5127	5291.5154	-0.0027	11	0	11	12	10	0	10	11
5431.0391	5431.0352	0.0039	11	2	10	12	10	2	9	11
5487.5192	5487.5095	0.0097	11	4	8	12	10	4	7	11
5487.8233	5487.8169	0.0064	11	3	9	12	10	3	8	11
5489.2628	5489.2808	-0.0180	11	4	7	12	10	4	6	11
5519.7818	5519.7680	0.0138	11	3	8	12	10	3	7	11
5557.1141	5557.1146	-0.0005	11	1	10	12	10	1	9	11
5607.0430	5607.0436	-0.0006	11	2	9	12	10	2	8	11
5652.9766	5652.9933	-0.0167	12	0	12	13	11	1	11	12
5728.7349	5728.7378	-0.0029	12	1	12	13	11	1	11	12
5755.4747	5755.4721	0.0026	12	0	12	13	11	0	11	12
5831.2029	5831.2166	-0.0137	12	1	12	13	11	0	11	12
5916.5634	5916.5713	-0.0079	12	2	11	13	11	2	10	12
5986.9619	5986.9467	0.0152	12	3	10	13	11	3	9	12
5989.8092	5989.8085	0.0007	6	3	3	6	5	2	4	5
5992.5563	5992.5685	-0.0122	12	4	8	13	11	4	7	12
6034.8715	6034.8615	0.0100	12	3	9	13	11	3	8	12
6042.3720	6042.3716	0.0004	12	1	11	13	11	1	10	12
6124.3041	6124.2989	0.0052	12	2	10	13	11	2	9	12
6143.7842	6143.7763	0.0079	13	0	13	14	12	1	12	13
6199.0003	6198.9964	0.0039	13	1	13	14	12	1	12	13
6219.5260	6219.5208	0.0052	13	0	13	14	12	0	12	13
6400.2063	6400.2111	-0.0048	13	2	12	12	12	2	11	11
6485.2648	6485.2549	0.0099	13	3	11	14	12	3	10	13
6491.5541	6491.5649	-0.0108	13	4	10	14	12	4	9	13
6522.0790	6522.0744	0.0046	13	1	12	14	12	1	11	13
6553.7513	6553.7456	0.0057	13	3	10	14	12	3	9	13
6638.6006	6638.6026	-0.0020	13	2	11	14	12	2	10	13
6668.5036	6668.5139	-0.0103	14	1	14	15	13	1	13	14
6683.9471	6683.9390	0.0081	14	0	14	15	13	0	13	14
6881.9403	6881.9517	-0.0114	14	2	13	15	13	2	12	14
6982.4637	6982.4679	-0.0042	14	3	12	15	13	3	11	14
6994.2734	6994.2651	0.0083	14	4	11	15	13	4	10	14
6996.3294	6996.3277	0.0017	14	1	13	15	13	1	12	14
7076.1390	7076.1401	-0.0011	14	3	11	15	13	3	10	14
7137.4304	7137.4258	0.0046	15	1	15	16	14	1	14	15
7148.8207	7148.8204	0.0003	15	0	15	16	14	0	14	15
7149.2215	7149.2262	-0.0047	14	2	12	15	13	2	11	14
7361.7932	7361.7976	-0.0044	15	2	14	16	14	2	13	15
7465.6843	7465.6976	-0.0133	15	1	14	16	14	1	13	15
7478.3196	7478.3274	-0.0078	15	3	13	16	14	3	12	15
7487.4073	7487.3998	0.0075	15	5	11	15	14	5	10	14
7488.2781	7488.2891	-0.0110	15	5	10	16	14	5	9	15
7497.2067	7497.2072	-0.0005	15	4	12	15	14	4	11	14
7497.2471	7497.2425	0.0046	15	4	12	16	14	4	11	15
7601.2862	7601.2868	-0.0006	15	3	12	16	14	3	11	15
7605.8670	7605.8542	0.0128	16	1	16	17	15	1	15	16
7614.1647	7614.1539	0.0108	16	0	16	17	15	0	15	16

7655.5555	7655.5613	-0.0058	15	2	13	16	14	2	12	15
7839.8186	7839.8179	0.0007	16	2	15	17	15	2	14	16
7931.1686	7931.1618	0.0068	16	1	15	17	15	1	14	16
7972.6009	7972.6005	0.0004	16	3	14	17	15	3	13	16
7991.8459	7991.8533	-0.0074	16	5	11	17	15	5	10	16

Table S73. Transition frequencies for $\mathscr{O}_{3w}$: O(29). J is the rotational angular momentum quantum number, K_a and K_c are the projections of J onto the principal axes at the prolate and oblate symmetric top limits, and F is the total angular momentum quantum number, which includes the nuclear spin, I(^{14}N) = 1.

Observed Frequency /MHz	Calculated Frequency /MHz	Difference / MHz	J	K_a	K_c	F	J'	K'_a	K'_c	F'
2341.9019	2341.9022	-0.0003	5	1	5	4	4	1	4	3
3331.4883	3331.4837	0.0046	7	0	7	8	6	0	6	7
3736.0954	3736.0963	-0.0009	8	1	8	9	7	1	7	8
3791.7433	3791.7423	0.0010	8	0	8	9	7	0	7	8
3855.9145	3855.9212	-0.0067	8	2	7	9	7	2	6	8
3959.5442	3959.5417	0.0025	8	1	7	9	7	1	6	8
4198.4136	4198.4185	-0.0049	9	1	9	10	8	1	8	9
4248.4403	4248.4368	0.0035	9	0	9	10	8	0	8	9
4333.6322	4333.6333	-0.0011	9	2	8	10	8	2	7	9
4364.0191	4364.0190	0.0001	9	3	7	10	8	3	6	9
4374.1899	4374.1927	-0.0028	9	3	6	10	8	3	5	9
4436.7176	4436.7162	0.0014	9	2	7	10	8	2	6	9
4445.9322	4445.9348	-0.0026	9	1	8	10	8	1	7	9
4659.5786	4659.5712	0.0074	10	1	10	11	9	1	9	10
4702.5863	4702.5924	-0.0061	10	0	10	11	9	0	9	10
4928.7482	4928.7405	0.0077	10	1	9	11	9	1	8	10
5119.6270	5119.6340	-0.0070	11	1	11	12	10	1	10	11
5155.2424	5155.2492	-0.0068	11	0	11	12	10	0	10	11
5407.3648	5407.3559	0.0089	11	1	10	12	10	1	9	11
5578.7131	5578.7131	-0.0000	12	1	12	13	11	1	11	12
5607.2689	5607.2621	0.0068	12	0	12	13	11	0	11	12
5821.6526	5821.6507	0.0019	12	3	10	12	11	3	9	11
5881.2502	5881.2519	-0.0017	12	1	11	13	11	1	10	12
6036.9312	6036.9309	0.0003	13	1	13	14	12	1	12	13
6059.2097	6059.2145	-0.0048	13	0	13	14	12	0	12	13
6228.9007	6228.9056	-0.0049	13	2	12	14	12	2	11	13
6350.0767	6350.0812	-0.0045	13	1	12	14	12	1	11	13
6451.0171	6451.0116	0.0055	13	2	11	14	12	2	10	13
6494.4214	6494.4148	0.0066	14	1	14	15	13	1	13	14
6698.4438	6698.4436	0.0002	14	2	13	15	13	2	12	14
6799.6621	6799.6685	-0.0064	14	4	11	14	13	4	10	13
6951.2961	6951.2891	0.0070	15	1	15	16	14	1	14	15
6964.0601	6964.0601	-0.0000	15	0	15	16	14	0	14	15
7442.7057	7442.7066	-0.0009	15	2	13	16	14	2	12	15
7754.7494	7754.7497	-0.0003	16	3	14	16	15	3	13	15
7754.7550	7754.7579	-0.0029	16	3	14	17	15	3	13	16

Table S74. Transition frequencies for $\mathscr{O}_{3w}$: O(32). J is the rotational angular momentum quantum number, K_a and K_c are the projections of J onto the principal axes at the prolate and oblate symmetric top limits, and F is the total angular momentum quantum number, which includes the nuclear spin, I(^{14}N) = 1.

Observed Frequency /MHz	Calculated Frequency /MHz	Difference / MHz	J	K_a	K_c	F	J'	K'_a	K'_c	F'
3498.5478	3498.5457	0.0021	7	2	5	8	6	2	4	7
3798.1697	3798.1693	0.0004	8	1	8	9	7	1	7	8
3853.2429	3853.2389	0.0040	8	0	8	9	7	0	7	8
3925.3994	3925.4136	-0.0142	8	2	7	9	7	2	6	8
4034.0152	4034.0141	0.0011	8	1	7	9	7	1	6	8
4267.7102	4267.7096	0.0006	9	1	9	10	8	1	8	9
4316.2006	4316.1979	0.0027	9	0	9	10	8	0	8	9
4528.2855	4528.2856	-0.0001	9	1	8	10	8	1	7	9
4528.2855	4528.2850	0.0005	9	1	8	9	8	1	7	8
4528.2855	4528.2701	0.0154	9	1	8	8	8	1	7	7
4735.9911	4735.9881	0.0030	10	1	10	11	9	1	9	10
4776.7853	4776.7838	0.0015	10	0	10	11	9	0	9	10
4940.8165	4940.8215	-0.0050	10	3	8	11	9	3	7	10
4940.8165	4940.8192	-0.0027	10	3	8	9	9	3	7	8
4961.9210	4961.9271	-0.0061	10	3	7	11	9	3	6	10
4961.9210	4961.9258	-0.0048	10	3	7	9	9	3	6	8
5018.3558	5018.3591	-0.0033	10	1	9	11	9	1	8	10
5203.1036	5203.1093	-0.0057	11	1	11	12	10	1	10	11
5203.1036	5203.1082	-0.0046	11	1	11	11	10	1	10	10
5503.5511	5503.5636	-0.0125	11	1	10	12	10	1	9	11
5556.6406	5556.6292	0.0114	11	2	9	12	10	2	8	11
5983.3660	5983.3647	0.0013	12	1	11	13	11	1	10	12
6134.4297	6134.4188	0.0109	13	1	13	14	12	1	12	13
6154.1780	6154.1778	0.0002	13	0	13	14	12	0	12	13
6457.5220	6457.5087	0.0133	13	1	12	14	12	1	11	13
7389.9466	7389.9575	-0.0109	15	1	14	16	14	1	13	15
7849.9569	7849.9561	0.0008	16	1	15	17	15	1	14	16

Table S75. Transition frequencies for $\mathscr{O}_{3w}$: O(35). J is the rotational angular momentum quantum number, K_a and K_c are the projections of J onto the principal axes at the prolate and oblate symmetric top limits, and F is the total angular momentum quantum number, which includes the nuclear spin, I(^{14}N) = 1.

Observed Frequency /MHz	Calculated Frequency /MHz	Difference / MHz	J	K_a	K_c	F	J'	K'_a	K'_c	F'
2830.4102	2830.4131	-0.0029	6	1	6	7	5	1	5	6
3503.0365	3503.0451	-0.0086	7	1	6	8	6	1	5	7
3765.6351	3765.6465	-0.0114	8	1	8	9	7	1	7	8
3820.9329	3820.9298	0.0031	8	0	8	9	7	0	7	8
3972.1015	3972.1155	-0.0140	8	2	6	8	7	2	5	7
3972.2038	3972.1885	0.0153	8	2	6	9	7	2	5	8
3996.4974	3996.5004	-0.0030	8	1	7	9	7	1	6	8
4280.3973	4280.4081	-0.0108	9	0	9	10	8	0	8	9
4486.6673	4486.6643	0.0030	9	1	8	10	8	1	7	9
4695.8014	4695.8047	-0.0033	10	1	10	11	9	1	9	10
4737.4360	4737.4444	-0.0084	10	0	10	11	9	0	9	10

4851.5132	4851.5204	-0.0072	10	2	9	11	9	2	8	10
4972.8876	4972.8774	0.0102	10	1	9	11	9	1	8	10
5159.1577	5159.1405	0.0172	11	1	11	12	10	1	10	11
5193.1332	5193.1375	-0.0043	11	0	11	12	10	0	10	11
5621.4769	5621.4669	0.0100	12	1	12	13	11	1	11	12
5648.3396	5648.3445	-0.0049	12	0	12	13	11	0	11	12
5806.3996	5806.3949	0.0047	12	2	11	13	11	2	10	12
5930.9867	5930.9792	0.0075	12	1	11	13	11	1	10	12
6103.6190	6103.6162	0.0028	13	0	13	14	12	0	12	13
6281.0943	6281.0911	0.0032	13	2	12	14	12	2	11	13
6543.6306	6543.6379	-0.0073	14	1	14	15	13	1	13	14
6559.2526	6559.2377	0.0149	14	0	14	15	13	0	13	14
7471.8324	7471.8284	0.0040	16	0	16	17	15	0	15	16
7694.0594	7694.0734	-0.0140	16	2	15	17	15	2	14	16
7922.6401	7922.6435	-0.0034	17	1	17	18	16	1	16	17

3.5 Clusters with 4 water molecules

Table S76. Predicted spectroscopic constants for the equilibrium and transition-state tetra-hydrated structures. The A,B,C parameters are the rotational constants, and $\chi_{aa}, \chi_{bb} - \chi_{cc}$, the nuclear quadrupole coupling constants associated to the ^{14}N atom. The predicted dipole moment components, μ, for the a-, b-, and c-type transitions and the predicted zero-point corrected relative energy, ΔE_{ZPC} are also shown. All theoretical parameters are expressed in the *principal axis system* (PAS).

Constants	$\mathcal{O}_{4w}$ (M$_1$)	$\mathcal{O}_{4w}$ (M$_2$)	$\mathcal{O}_{4w}$ (M$_3$)	$\mathcal{O}_{4w}$ (M$_4$)	$\mathcal{O}_{4w}$ (TS$_1$)	$\mathcal{O}_{4w}$ (TS$_2$)	$\mathcal{O}_{4w}$ (TS$_3$)	$\mathcal{O}_{4w}$ (TS$_4$)
A /MHz	726	723	713	710	715	714	714	713
B /MHz	219	219	223	221	222	222	221	221
C /MHz	208	209	213	213	212	213	212	212
χ_{aa}	-4.1	-4.1	-4.1	-4.0	-4.1	-4.1	-4.0	-4.0
$\chi_{bb} - \chi_{cc}$ /MHz	-2.1	-2.2	-2.2	-2.3	-2.2	-2.2	-2.3	-2.3
μ_a /D	3.9	-3.8	-2.1	-6.0	-2.6	-2.5	-4.9	4.9
μ_b /D	-1.7	-1.7	-2.4	-1.1	-2.4	2.4	-1.6	1.6
μ_c /D	-1.4	-1.7	-0.1	-0.2	0.9	1.1	0.3	-0.1
ΔE_{ZPC} /kJ mol^{-1}	0	+0.5	+1.4	+1.8	+0.2	+ 0.3	+0.8	+0.88

3.5.1 Equilibrium and transition state geometries

Table S77. Equilibrium geometry of $\mathscr{O}_{4w}$ (M_1).

Atom	x /Å	y /Å	z /Å
C	-2.02768754034153	1.18719460558863	1.63538447101975
C	-2.60199544328074	-0.25777687452584	1.67985235210372
C	-2.45973201711249	-0.78666669605808	0.21962560141941
C	-0.96078152037951	-1.01149914855464	0.07215920552908
C	-0.38523858033893	0.37520543611250	0.06367189033895
C	-1.55039271588934	1.31729380547577	0.16805738415249
C	-3.30876504567277	-1.99333299368714	-0.11172424283112
C	-2.63169935533184	0.52111320866904	-0.61757374982432
C	-2.27765753790580	0.34381047749218	-2.09720459862058
C	-4.02867612992810	1.13443670928431	-0.52643878500504
O	-0.37057486505406	-2.07247809439184	-0.00381254530685
N	0.88208952502726	0.57520246694759	0.01951683120023
O	1.23337738387320	1.89492278970021	0.05005145354405
H	-2.79136143131054	1.93339773333353	1.86342427913302
H	-1.20341761524756	1.33566452359492	2.33606724966847
H	-2.06943760219852	-0.90149548146079	2.38583628405036
H	-3.65618783429821	-0.26958992201931	1.96806151865901
H	-1.34513794240075	2.33237313145231	-0.17219656328433
H	-3.15297008395652	-2.31831980149312	-1.14356874073713
H	-3.04576572627949	-2.83166122274611	0.53847411996098
H	-4.37010866127154	-1.77178876673059	0.02807020562519
H	-3.01064338783146	-0.29786461793754	-2.59440250445252
H	-2.28650615445605	1.31374743848457	-2.60377129819751
H	-1.28876037108141	-0.09818966415881	-2.24970561968704
H	-4.02852121523026	2.12571742819314	-0.99082773810172
H	-4.74987942498666	0.51651350269258	-1.06905280309017
H	-4.38913339443016	1.24592484960847	0.49655394263717
H	2.23302195674159	1.85897070032764	0.02033950009145
O	4.50538821002181	-0.51578716334402	1.75057421569524
H	3.93021281926696	-1.12882245593962	1.25911936017314
H	5.39223840661865	-0.88311560904256	1.67164579679216
O	4.48481467416557	-0.45294641438194	-1.88998955076578
H	3.88005839889734	-1.07202665014400	-1.44162170306083
H	4.28994211360250	-0.53414159526311	-2.82901967524149
O	2.65918007271984	-1.65057662051606	-0.10714410970654
H	2.07361855146000	-0.85988614067999	-0.06471342060673
H	2.02332116572316	-2.37806827312545	-0.09236629493275
O	3.86196467465063	1.63136841134819	-0.02423081825691
H	4.15996937838885	1.07834961322735	0.72189091385434
H	4.14627528735685	1.12213492676763	-0.80448487553883

Table S78. Equilibrium geometry of $\mathscr{O}_{4w}$ (M_2).

Atom	x /Å	y /Å	z /Å
C	-2.07495595306845	1.19633847459013	1.63469196863161
C	-2.65839008608014	-0.24543971951595	1.66432777646424
C	-2.47368544160504	-0.77802799198062	0.21045576618851
C	-0.97209637855802	-1.01057248988819	0.10957233071826
C	-0.38942051155773	0.37341380661902	0.11144874341989
C	-1.55235890975901	1.32124265402031	0.18269003030448
C	-3.31795555529835	-1.98137677679429	-0.14486283053528
C	-2.61319278106710	0.52888195214690	-0.63439257131237
C	-2.21510966363884	0.34678484991593	-2.10223521293220
C	-4.00912906063022	1.14971524666521	-0.58675697459176
O	-0.38375491758982	-2.07406686131636	0.05646095382113
N	0.87966660057631	0.56579031604733	0.08231963964716
O	1.23919318544117	1.88306525699785	0.11418273796540
H	-2.84108532278650	1.94716661072224	1.83814102197145
H	-1.27186602539573	1.34195834918429	2.36031575187859
H	-2.15192182977875	-0.89092085803891	2.38784462178844
H	-3.72108380795329	-0.25079689821012	1.91976955544679
H	-1.33182532836645	2.33459945588000	-0.15336104757946
H	-3.08136598550892	-2.81918408835638	0.51613999221441
H	-4.38215718886401	-1.75371167948143	-0.04099989071710
H	-3.12963761402241	-2.31036952552641	-1.16994372609422
H	-1.22415166783809	-0.09968947392706	-2.22496268779220
H	-2.93601246279959	-0.29241204640446	-2.61987743282748
H	-2.20415619836949	1.31558580997088	-2.61083262591949
H	-4.40054503400941	1.26489079073405	0.42446120597792
H	-3.98940950240286	2.14009232106739	-1.05262410645865
H	-4.71653887650746	0.53475633576829	-1.15047937025221
H	2.23664948133530	1.84234933856453	0.04525468896879
O	4.60674525514002	-0.57279118223583	1.64780621814055
H	3.95515133543389	-1.16145259832779	1.22499156759864
H	4.50695535491811	-0.70927042933262	2.59519858871680
O	3.86078592435851	1.60232347409137	-0.06007827487858
H	4.09712474036936	1.09439825341102	-0.85854975294737
H	4.20625423516686	1.05038992705103	0.66443405068890
O	2.65074883529114	-1.66696503506621	-0.05894833106825
H	2.05899141263474	-0.88104106557420	-0.01596007378958
H	2.02204743509492	-2.40050951227452	-0.07460317188902
O	4.33869856144396	-0.43537347482004	-2.00705670703865
H	5.22518236242306	-0.81190082165448	-2.01506495453924
H	3.79810138382842	-1.06616069472191	-1.49885746738881

Table S79. Equilibrium geometry of $\mathscr{O}_{4w}$ (M$_3$).

Atom	x /Å	y /Å	z /Å
C	-2.01635684837593	1.19030066076609	1.62725505242247
C	-2.59651827834049	-0.25249368512282	1.66578675413562
C	-2.43744211532414	-0.78321238387357	0.20796150693482
C	-0.93765942050183	-1.01397779549680	0.08020126805826
C	-0.35682995206280	0.37036639092448	0.07629947850424
C	-1.51969819304358	1.31684028204067	0.16624661839947
C	-3.28659614196915	-1.98707351258446	-0.13311773655577
C	-2.59369523929194	0.52421943989584	-0.63277532438383
C	-2.22105085205716	0.34439966256502	-2.10754948753827
C	-3.98943338377275	1.14279272727099	-0.55987638363845
O	-0.35212412467134	-2.07845732864205	0.01478130307225
N	0.91127116330416	0.56792914522439	0.04068190680015
O	1.26380127646140	1.88690817886800	0.07362443507810
H	-2.78028467458653	1.93946467506176	1.84467604854782
H	-1.20066856051794	1.33644175868280	2.33841704132312
H	-2.07535392554932	-0.89750824730380	2.37909994933709
H	-3.65437469189093	-0.26023790455814	1.94049149739492
H	-1.30570606692424	2.33055793148030	-0.17264922445908
H	-3.03493994621908	-2.82590348363614	0.52094214033699
H	-4.34879399473232	-1.76141963952712	-0.00689430600316
H	-3.11894709195576	-2.31325628356911	-1.16270322914756
H	-1.23111496309469	-0.09919703539178	-2.24776664786392
H	-2.94917260728360	-0.29618879771014	-2.61325242213602
H	-2.22143947545358	1.31377799396474	-2.61517347008983
H	-4.36313563947029	1.25543178208725	0.45827375281933
H	-3.97925823915415	2.13411322358999	-1.02403342903505
H	-4.70564521957632	0.52774457998996	-1.11229546977206
H	2.26249937423426	1.84989124280137	0.03321831812334
O	4.37259137633288	-0.53311101931784	1.81991752228399
H	3.83653810243872	-1.16409028838860	1.30645308284382
H	5.19624704344486	-0.98840175122317	2.02045186555699
O	3.89246743594816	1.59613781968745	-0.02877035711577
H	4.13344534223242	1.08444296711171	-0.82229305558692
H	4.17744849840790	1.02803174837070	0.70995302324855
O	2.65456588704566	-1.67959621661152	-0.11304253353426
H	2.09191603309883	-0.87166319054103	-0.06504515853812
H	1.98908139474360	-2.38112829840263	-0.11662793187033
O	4.28080015490116	-0.39963493397223	-2.04192172353695
H	5.09678705492110	-0.83563114705651	-2.30646039180211
H	3.76951209800486	-1.06606514275402	-1.54849504931383

Table S80. Equilibrium geometry of $\mathscr{O}_{4w}$ (M_4).

Atom	x /Å	y /Å	z /Å
C	-2.02107494622529	1.18328335551568	1.64042407449339
C	-2.60931342833975	-0.25662315696209	1.66979069281366
C	-2.44757353112113	-0.78134555026671	0.21028100809108
C	-0.94858723419745	-1.01852481677090	0.08605091190788
C	-0.36054120350960	0.36258628173966	0.09006058516289
C	-1.51843942786289	1.31486673154058	0.18187576866375
C	-3.30168055400241	-1.97928789803727	-0.14002494632739
C	-2.59410494417438	0.53150285293128	-0.62437616653551
C	-2.21838389237020	0.35674342180820	-2.09898337703640
C	-3.98695132569294	1.15711070215099	-0.55320171630693
O	-0.36323098598399	-2.08292782227362	0.01645993091299
N	0.90995844910243	0.54812681493426	0.05474834071620
O	1.27686646980027	1.86256172591008	0.09254146344454
H	-2.78144092858065	1.93566296951976	1.85916862712284
H	-1.20723083098702	1.32184230593449	2.35535697904343
H	-2.09490694929391	-0.90828623582485	2.38207320929475
H	-3.66818135949431	-0.25977108589515	1.94065456281990
H	-1.29907828254630	2.32962135584824	-0.15076644168957
H	-3.05870622772522	-2.82193106125868	0.51249763988722
H	-4.36346115976842	-1.74853919322469	-0.01949387567458
H	-3.12935452255365	-2.30288252299367	-1.16968128770110
H	-1.23127323199759	-0.09371359980836	-2.23780527084111
H	-2.94936029892255	-0.27627744693277	-2.61008011059248
H	-2.21054220389960	1.32862259329794	-2.60189652999474
H	-4.36326264677704	1.26761845635421	0.46423842469351
H	-3.97032319886027	2.15029707768066	-1.01327532171476
H	-4.70472789254951	0.54806777483068	-1.11023163223507
H	2.27748251353973	1.81839426686695	0.04570069295487
O	4.45526871629614	-0.53551701029906	1.78862822258552
H	4.14323681141486	-0.62033501566458	2.69517462747078
H	3.91044222300008	-1.15166708748904	1.26720302492913
O	3.89669349155591	1.60396594496653	-0.02331357205289
H	4.15894966602286	1.10923912504774	-0.82142686768202
H	4.20975897130440	1.04329064758427	0.71024861992475
O	2.68548905041392	-1.68727767973690	-0.12669692051294
H	2.07952743206595	-0.91437651378661	-0.06510096757609
H	2.07587841016319	-2.43654472885360	-0.13617433488265
O	4.34230086969079	-0.37600777142503	-2.03455605400661
H	3.98712028128449	-0.38823899472281	-2.92901868199275
H	3.82237196558120	-1.03334719953582	-1.53840563067744

Table S81. Transition-state geometry of $\mathscr{O}_{4w}$ (TS_1).

Atom	x /Å	y /Å	z /Å
C	-1.67048708576008	1.18730830218392	1.71188475990717
C	-2.24257544414641	-0.25876339847546	1.75009677327976
C	-2.09504385635438	-0.78261277997602	0.28867858676785
C	-0.59534298000678	-1.00407472234815	0.14442238684166
C	-0.02239791355716	0.38330671746975	0.14074231630611
C	-1.18970238848763	1.32283741546703	0.24630759777137
C	-2.94060007032313	-1.99007498813723	-0.04906737478700
C	-2.26725969498940	0.52746118725538	-0.54496513786528
C	-1.90879555877510	0.35563548742825	-2.02421209002772
C	-3.66575741137788	1.13771349448597	-0.45553732752189
O	-0.00324374302896	-2.06427156355216	0.06741608076688
N	1.24470158141045	0.58698653902163	0.09518760422867
O	1.59124958826130	1.90700375936332	0.13026163204533
H	-2.43588894067615	1.93154301442890	1.94079763057446
H	-0.84802801740964	1.33460560975655	2.41499454057415
H	-1.71107086090240	-0.90392678749763	2.45561784125718
H	-3.29766795848627	-0.27329996089344	2.03509683880239
H	-0.98527854875291	2.33933443718655	-0.09030490066223
H	-2.67970582511237	-2.82926635866771	0.60091985502394
H	-4.00303957072495	-1.77050427658353	0.08569953437275
H	-2.77886530697862	-2.31280420033970	-1.08070520423350
H	-0.91825179212382	-0.08281082963478	-2.17596687486806
H	-2.63904446339441	-0.28627447935896	-2.52522839197171
H	-1.91884335449161	1.32711919221012	-2.52777680587950
H	-4.02888060093410	1.24614399606325	0.56689015911086
H	-3.66641310344103	2.13008394422694	-0.91763522404165
H	-4.38452073600683	0.51976639217830	-1.00141271483380
H	2.59008809948017	1.87669430834807	0.08620127810993
O	4.77727626630404	-0.48775188962273	1.81726481577060
H	4.23575881150996	-1.10947555464706	1.29786802916205
H	5.64231966435380	-0.90243602006205	1.89699548001278
O	4.22309281099405	1.65892998788053	0.02153666265495
H	4.49391482573739	1.19632901347699	-0.78979643940264
H	4.53119597386383	1.07177036845974	0.73696366756632
O	3.00720347246109	-1.64684935869842	-0.07234120119239
H	2.43035662375229	-0.84962096898493	-0.02070471194649
H	2.35936263366496	-2.36391570662511	-0.05000188639749
O	4.61217751559710	-0.33397052932831	-1.97994843343635
H	5.05733012010537	-0.76934920093298	-2.71216599620828
H	4.10067723874624	-1.01851959252482	-1.51407335563114

Table S82. Transition-state geometry of $\mathscr{O}_{4w}$ (TS_2).

Atom	x /Å	y /Å	z /Å
C	-1.71212219009350	1.21776766612796	1.64953162832374
C	-2.29253205654843	-0.22493412159478	1.69025894158751
C	-2.10642980497855	-0.76849055676880	0.24055431224210
C	-0.60443135136855	-0.99894162131834	0.14166198264562
C	-0.02444337125461	0.38560338394677	0.13495153607937
C	-1.18936137916138	1.33190681720550	0.19666680397686
C	-2.94828376844593	-1.97618134193871	-0.10547479393817
C	-2.24827537529865	0.53141803325541	-0.61455475558372
C	-1.84904832839074	0.33873540702442	-2.08074369968465
C	-3.64553269839896	1.14962457416973	-0.57242941619036
O	-0.01583595269216	-2.06285895921092	0.09550432617674
N	1.24428208276122	0.58263571832233	0.11360010665855
O	1.59662261771930	1.90155044237882	0.13731951906746
H	-2.47992681321276	1.96851115915129	1.84696258107731
H	-0.90925830163578	1.37042618459665	2.37386996050999
H	-1.78460651752522	-0.86349005007295	2.41883834388896
H	-3.35529460621170	-0.23064470807599	1.94548047232499
H	-0.97025418343186	2.34289499483430	-0.14721981276465
H	-2.71104304512996	-2.80784349005837	0.56301480624263
H	-4.01299592001743	-1.74942865385367	-0.00478777541545
H	-2.75821610056444	-2.31360539697705	-1.12750724779740
H	-0.85676477596440	-0.10594142675086	-2.19928213854023
H	-2.56802847844457	-0.30635005662571	-2.59381235007514
H	-1.84024560368760	1.30359425806869	-2.59684913622478
H	-4.03763880895529	1.27156437369627	0.43771742732343
H	-3.62746932727454	2.13659865173805	-1.04557871400175
H	-4.35139951235413	0.52910272355957	-1.13200984578970
H	2.59524793608599	1.86645085564570	0.09382704551548
O	4.77514631198781	-0.47766999918736	1.83195331638687
H	4.21856534960313	-1.12961962276335	1.37103016129612
H	5.27857805625303	-0.95985766178120	2.49368551366065
O	4.22645952500713	1.64093236008298	0.00343036549876
H	4.46643132776839	1.10059842025128	-0.77242566132273
H	4.56350061846284	1.12641972190766	0.75657434598567
O	2.99801563850298	-1.66126640851227	-0.00648520412980
H	2.42537910129149	-0.86088615625912	0.04311546384362
H	2.34474816030156	-2.37360381671626	-0.02072948477267
O	4.60441357550729	-0.38713673338335	-1.96693620800199
H	5.45544158511022	-0.79768594623173	-2.15076990380621
H	4.10660638467878	-1.03989901788260	-1.44195281227304

Table S83. Transition-state geometry of $\mathscr{O}_{4w}$ (TS$_3$).

Atom	x /Å	y /Å	z /Å
C	-1.65548017689907	1.18717611816608	1.67489725270670
C	-2.24021530092441	-0.25397428251461	1.70913561618662
C	-2.08162462371862	-0.78145236117300	0.25024311664144
C	-0.58226982937305	-1.01568177513456	0.12187064226060
C	0.00246037444895	0.36665405372376	0.12294915936162
C	-1.15775649868563	1.31642471287876	0.21438750815068
C	-2.93398261772364	-1.98204736569364	-0.09480857313637
C	-2.23390275630429	0.52898549395099	-0.58686421613458
C	-1.86183468577069	0.35178934353897	-2.06209509925041
C	-3.62796793245565	1.15139627592164	-0.51309613561796
O	0.00281071017009	-2.08010623134297	0.05110644177778
N	1.27211005449872	0.55751899889560	0.09154577663641
O	1.63208982128068	1.87371637266110	0.12706299206531
H	-2.41693016239700	1.93820695878788	1.89453633642184
H	-0.83939163884041	1.32884772099068	2.38656568961617
H	-1.72188107654166	-0.90268003357645	2.42121085281139
H	-3.29830474391019	-0.25903219372367	1.98306776865315
H	-0.94135778479470	2.33077265341480	-0.12127115631845
H	-2.68713463028441	-2.82260079327380	0.55888714743865
H	-3.99586677909596	-1.75329269746575	0.02846811646466
H	-2.76420499417345	-2.30767598286371	-1.12423305105709
H	-0.87428246630619	-0.09713182140278	-2.20245588479505
H	-2.59297209515252	-0.28359696917211	-2.57001931118065
H	-1.85700341154581	1.32266224767349	-2.56694732032840
H	-4.00084922621691	1.26460880651015	0.50527080577852
H	-3.61530792623051	2.14299605531104	-0.97666220924990
H	-4.34629142813693	0.53875466500926	-1.06545467716503
H	2.63284843479779	1.83473779574806	0.09471376036060
O	4.82105251235498	-0.49382350921465	1.82229315187428
H	4.27080221382183	-1.14581335039592	1.35502111266864
H	5.07201139889184	-0.89225910560859	2.66015004623905
O	4.25665765962681	1.63515110891087	0.03314623920048
H	4.53053782583106	1.10961312140854	-0.74126382002125
H	4.58452788629405	1.12013771466563	0.79071023724746
O	3.04442490016923	-1.68046822617637	-0.03854379241811
H	2.45206155960046	-0.89625253306418	0.01671526062850
H	2.41668783195593	-2.41478660242456	-0.04856250906364
O	4.73002516101662	-0.38463014071223	-1.92772022305912
H	4.40964642373414	-0.41621278241723	-2.83472680748035
H	4.19605801698857	-1.03663146081657	-1.43923024491417

Table S84. Transition-state geometry of $\mathscr{O}_{4w}$ (TS$_4$).

Atom	x /Å	y /Å	z /Å
C	-1.69357923472596	1.20924370397521	1.65528874264784
C	-2.28358089946656	-0.22975661800157	1.69023717202128
C	-2.08890537139882	-0.77362426977539	0.24182053064641
C	-0.58742972060705	-1.01321716668543	0.15515631443003
C	0.00113960266203	0.36758182863567	0.15327122725290
C	-1.15814176596306	1.32128030163011	0.20688412523295
C	-2.93551242814216	-1.97577327779121	-0.11184776766128
C	-2.21575888501535	0.52789588854806	-0.61326365268580
C	-1.80632958050211	0.33361056995340	-2.07642049871871
C	-3.60937894730751	1.15490478142744	-0.58200798446622
O	-0.00374344122263	-2.07995898953772	0.11411953907418
N	1.27159921815833	0.55476113358996	0.14018209326666
O	1.63473097691322	1.87042265983319	0.16819352699647
H	-2.45804675424310	1.96476180532173	1.84728732497800
H	-0.89577869335713	1.35645208742271	2.38636569666232
H	-1.78614675636976	-0.87224384647749	2.42261168818713
H	-3.34841436289631	-0.22861145861256	1.93666616605921
H	-0.93021684353702	2.33124709714756	-0.13432469342169
H	-2.70790083551087	-2.80976356550999	0.55707421424052
H	-3.99955767580750	-1.74296032330669	-0.01842504060598
H	-2.74024823627377	-2.31299699101110	-1.13298050917050
H	-0.81659791233891	-0.11876240003524	-2.18697174331718
H	-2.52605572071331	-0.30552038008469	-2.59582173271567
H	-1.78603691341623	1.29885830812264	-2.59149014184711
H	-4.00793962584978	1.28066485195881	0.42511623428249
H	-3.58184009692724	2.14111608050365	-1.05626326801734
H	-4.31513892603873	0.53802690978730	-1.14571663323141
H	2.63510059807083	1.82903645017261	0.12990094334247
O	4.86681594126331	-0.52166545346843	1.84512148483694
H	4.61812433508319	-0.61531574224383	2.77013359709808
H	4.29364312759668	-1.13948339426332	1.35663550087476
O	4.25865206127529	1.62492455280419	0.06345739992477
H	4.52721757234513	1.16111076548549	-0.74859024047161
H	4.58588767368226	1.04909439139553	0.77913248534535
O	3.03764750849316	-1.68805113126789	0.00655565948224
H	2.44727670501406	-0.90141740357918	0.04680311148871
H	2.40795800277669	-2.42075762418537	0.01152082958788
O	4.67539793412542	-0.38026753483903	-1.90018352819807
H	4.86163936436650	-0.72266067969701	-2.77869836732419
H	4.15944900580479	-1.06218591734209	-1.43652980610681

3.5.2 Transition frequencies

Table S85. Transition frequencies for $\mathcal{O}_{4w}$. J is the rotational angular momentum quantum number, K_a and K_c are the projections of J onto the principal axes at the prolate and oblate symmetric top limits, and F is the total angular momentum quantum number, which includes the nuclear spin, I(^{14}N) = 1.

Observed Frequency /MHz	Calculated Frequency /MHz	Difference / MHz	J	K_a	K_c	F	J'	K'_a	K'_c	F'
2169.1768	2169.1926	-0.0158	5	0	5	6	4	0	4	5
2575.8486	2575.8415	0.0071	6	1	6	7	5	1	5	6
2601.4610	2601.4474	0.0136	6	0	6	7	5	0	5	6
3032.8599	3032.8681	-0.0082	7	0	7	8	6	0	6	7
3039.8154	3039.7998	0.0156	7	2	6	8	6	2	5	7
3073.3803	3073.4041	-0.0238	7	1	6	8	6	1	5	7
3073.3803	3073.4041	-0.0238	7	1	6	7	6	1	5	6
3073.3803	3073.4041	-0.0238	7	1	6	6	6	1	5	5
3433.1952	3433.2016	-0.0064	8	1	8	9	7	1	7	8
3463.3452	3463.3550	-0.0098	8	0	8	9	7	0	7	8
3473.5513	3473.5668	-0.0155	8	2	7	9	7	2	6	8
3473.5513	3473.5668	-0.0155	8	2	7	8	7	2	6	7
3473.5513	3473.5668	-0.0155	8	2	7	7	7	2	6	6
3485.5435	3485.5550	-0.0115	8	2	6	9	7	2	5	8
3511.6347	3511.6534	-0.0187	8	1	7	9	7	1	6	8
3861.5202	3861.5318	-0.0116	9	1	9	10	8	1	8	9
3892.8262	3892.8390	-0.0128	9	0	9	10	8	0	8	9
3907.1442	3907.1385	0.0057	9	2	8	10	8	2	7	9
3907.1442	3907.1385	0.0057	9	2	8	9	8	2	7	8
3907.1442	3907.1385	0.0057	9	2	8	8	8	2	7	7
3911.9056	3911.8899	0.0157	9	3	7	10	8	3	6	9
3911.9056	3911.8899	0.0157	9	3	7	9	8	3	6	8
3911.9056	3911.8899	0.0157	9	3	7	8	8	3	6	7
3912.4900	3912.5103	-0.0203	9	3	6	10	8	3	5	9
3912.4900	3912.5103	-0.0203	9	3	6	9	8	3	5	8
3912.4900	3912.5103	-0.0203	9	3	6	8	8	3	5	7
3924.0568	3924.0495	0.0073	9	2	7	10	8	2	6	9
3924.0568	3924.0495	0.0073	9	2	7	9	8	2	6	8
3924.0568	3924.0495	0.0073	9	2	7	8	8	2	6	7
3949.5374	3949.5511	-0.0137	9	1	8	10	8	1	7	9
4289.5980	4289.6077	-0.0097	10	1	10	11	9	1	9	10
4321.2821	4321.2909	-0.0088	10	0	10	11	9	0	9	10
4340.5101	4340.4913	0.0188	10	2	9	11	9	2	8	10
4340.5101	4340.4913	0.0188	10	2	9	10	9	2	8	9
4340.5101	4340.4913	0.0188	10	2	9	9	9	2	8	8
4346.9305	4346.9346	-0.0041	10	3	8	11	9	3	7	10
4346.9434	4346.9346	0.0088	10	3	8	10	9	3	7	9
4346.9434	4346.9346	0.0088	10	3	8	9	9	3	7	8
4347.9800	4347.9951	-0.0151	10	3	7	11	9	3	6	10
4347.9800	4347.9951	-0.0151	10	3	7	10	9	3	6	9
4347.9800	4347.9951	-0.0151	10	3	7	9	9	3	6	8
4363.3470	4363.3501	-0.0031	10	2	8	11	9	2	7	10
4387.0256	4387.0356	-0.0100	10	1	9	11	9	1	8	10
4717.4077	4717.4191	-0.0114	11	1	11	12	10	1	10	11
4748.7198	4748.7272	-0.0074	11	0	11	12	10	0	10	11
4773.5979	4773.6019	-0.0040	11	2	10	12	10	2	9	11
4781.0298	4781.0216	0.0082	11	4	7	12	10	4	6	11

4782.0574	4782.0564	0.0010	11	3	9	12	10	3	8	11
4783.7968	4783.7733	0.0235	11	3	8	12	10	3	7	11
4783.7968	4783.7733	0.0235	11	3	8	11	10	3	7	10
4783.7968	4783.7733	0.0235	11	3	8	10	10	3	7	9
4803.4158	4803.4134	0.0024	11	2	9	10	10	2	8	9
4824.0299	4824.0393	-0.0094	11	1	10	11	10	1	9	10
5144.9546	5144.9606	-0.0060	12	1	12	12	11	1	11	11
5175.2100	5175.2119	-0.0019	12	0	12	13	11	0	11	12
5206.4460	5206.4481	-0.0021	12	2	11	13	11	2	10	12
5217.2607	5217.2421	0.0186	12	3	10	13	11	3	9	12
5217.2607	5217.2421	0.0186	12	3	10	12	11	3	9	11
5217.2607	5217.2421	0.0186	12	3	10	11	11	3	9	10
5219.9270	5219.8995	0.0275	12	3	9	13	11	3	8	12
5219.9293	5219.8995	0.0298	12	3	9	11	11	3	8	10
5244.1299	5244.1479	-0.0180	12	2	10	13	11	2	9	12
5260.4775	5260.4887	-0.0112	12	1	11	13	11	1	10	12
5572.2250	5572.2311	-0.0061	13	1	13	14	12	1	12	13
5600.8481	5600.8509	-0.0028	13	0	13	14	12	0	12	13
5639.0036	5639.0082	-0.0046	13	2	12	14	12	2	11	13
5652.4829	5652.4739	0.0090	13	3	11	14	12	3	10	13
5652.4829	5652.4739	0.0090	13	3	11	13	12	3	10	12
5652.4829	5652.4739	0.0090	13	3	11	12	12	3	10	11
5656.4497	5656.4342	0.0155	13	3	10	14	12	3	9	13
5656.4497	5656.4342	0.0155	13	3	10	13	12	3	9	12
5656.4497	5656.4342	0.0155	13	3	10	12	12	3	9	11
5685.4149	5685.4183	-0.0034	13	2	11	14	12	2	10	13
5696.2947	5696.3043	-0.0096	13	1	12	14	12	1	11	13
5999.2231	5999.2340	-0.0109	14	1	14	15	13	1	13	14
6025.7808	6025.7813	-0.0005	14	0	14	15	13	0	13	14
6071.2431	6071.2619	-0.0188	14	2	13	15	13	2	12	14
6084.9007	6084.9057	-0.0050	14	5	9	15	13	5	8	14
6084.9007	6084.9022	-0.0015	14	5	10	15	13	5	9	14
6084.9007	6084.9057	-0.0050	14	5	9	14	13	5	8	13
6084.9007	6084.9022	-0.0015	14	5	10	14	13	5	9	13
6084.9007	6084.9057	-0.0050	14	5	9	13	13	5	8	12
6084.9007	6084.9022	-0.0015	14	5	10	13	13	5	9	12
6087.7545	6087.7298	0.0247	14	3	12	15	13	3	11	14
6087.7545	6087.7298	0.0247	14	3	12	14	13	3	11	13
6087.7545	6087.7298	0.0247	14	3	12	13	13	3	11	12
6093.4375	6093.4426	-0.0051	14	3	11	15	13	3	10	14
6093.4375	6093.4426	-0.0051	14	3	11	14	13	3	10	13
6093.4375	6093.4426	-0.0051	14	3	11	13	13	3	10	12
6127.0584	6127.0569	0.0015	14	2	12	15	13	2	11	14
6131.4024	6131.4019	0.0005	14	1	13	15	13	1	12	14
6425.9688	6425.9764	-0.0076	15	1	15	16	14	1	14	15
6450.1668	6450.1560	0.0108	15	0	15	16	14	0	14	15
6503.1747	6503.1901	-0.0154	15	2	14	16	14	2	13	15
6518.8886	6518.8852	0.0034	15	6	9	16	14	6	8	15
6518.8886	6518.8851	0.0035	15	6	10	16	14	6	9	15
6518.8886	6518.8852	0.0034	15	6	9	15	14	6	8	14
6518.8886	6518.8851	0.0035	15	6	10	15	14	6	9	14
6518.8886	6518.8852	0.0034	15	6	9	14	14	6	8	13
6518.8886	6518.8851	0.0035	15	6	10	14	14	6	9	13
6519.9680	6519.9680	0.0000	15	5	10	16	14	5	9	15
6519.9680	6519.9614	0.0066	15	5	11	16	14	5	10	15

6519.9680	6519.9680	0.0000	15	5	10	15	14	5	9	14
6519.9680	6519.9614	0.0066	15	5	11	15	14	5	10	14
6519.9680	6519.9680	0.0000	15	5	10	14	14	5	9	13
6519.9680	6519.9614	0.0066	15	5	11	14	14	5	10	13
6522.9760	6522.9840	-0.0080	15	3	13	16	14	3	12	15
6530.9970	6530.9928	0.0042	15	3	12	16	14	3	11	15
6565.6979	6565.6940	0.0039	15	1	14	16	14	1	13	15
6568.8563	6568.8780	-0.0217	15	2	13	16	14	2	12	15
6852.4612	6852.4688	-0.0076	16	1	16	17	15	1	15	16
6852.4612	6852.4688	-0.0076	16	1	16	16	15	1	15	15
6852.4612	6852.4688	-0.0076	16	1	16	15	15	1	15	14
6874.1310	6874.1278	0.0032	16	0	16	17	15	0	15	16
6874.1310	6874.1278	0.0032	16	0	16	16	15	0	15	15
6874.1310	6874.1278	0.0032	16	0	16	15	15	0	15	14
6934.7756	6934.7757	-0.0001	16	2	15	17	15	2	14	16
6957.3784	6957.3803	-0.0019	16	4	13	17	15	4	12	16
6957.3784	6957.3803	-0.0019	16	4	13	16	15	4	12	15
6957.3784	6957.3803	-0.0019	16	4	13	15	15	4	12	14
6969.1502	6969.1529	-0.0027	16	3	13	17	15	3	12	16
6999.0880	6999.0931	-0.0051	16	1	15	17	15	1	14	16
7278.7214	7278.7247	-0.0033	17	1	17	18	16	1	16	17
7278.7214	7278.7247	-0.0033	17	1	17	17	16	1	16	16
7278.7214	7278.7247	-0.0033	17	1	17	16	16	1	16	15
7297.8478	7297.8359	0.0119	17	0	17	18	16	0	16	17
7365.9909	7366.0035	-0.0126	17	2	16	18	16	2	15	17
7393.0143	7393.0022	0.0121	17	4	14	18	16	4	13	17
7393.3577	7393.3678	-0.0101	17	3	15	18	16	3	14	17
7393.7839	7393.7890	-0.0051	17	4	13	18	16	4	12	17
7407.9791	7407.9866	-0.0075	17	3	14	18	16	3	13	17
7407.9791	7407.9866	-0.0075	17	3	14	17	16	3	13	16
7407.9791	7407.9866	-0.0075	17	3	14	16	16	3	13	15
7431.5222	7431.5157	0.0065	17	1	16	18	16	1	15	17
7431.5222	7431.5157	0.0065	17	1	16	17	16	1	15	16
7431.5222	7431.5157	0.0065	17	1	16	16	16	1	15	15
7452.3096	7452.3254	-0.0158	17	2	15	18	16	2	14	17
7704.7519	7704.7593	-0.0074	18	1	18	19	17	1	17	18
7721.4025	7721.3975	0.0050	18	0	18	19	17	0	17	18
7796.8611	7796.8605	0.0006	18	2	17	19	17	2	16	18
7828.4347	7828.4318	0.0029	18	3	16	19	17	3	15	18
7828.4347	7828.4318	0.0029	18	3	16	18	17	3	15	17
7828.4347	7828.4318	0.0029	18	3	16	17	17	3	15	16
7828.7420	7828.7381	0.0039	18	4	15	19	17	4	14	18
7828.7420	7828.7381	0.0039	18	4	15	18	17	4	14	17
7828.7420	7828.7381	0.0039	18	4	15	17	17	4	14	16
7829.9151	7829.9119	0.0032	18	4	14	19	17	4	13	18
7829.9151	7829.9119	0.0032	18	4	14	18	17	4	13	17
7829.9151	7829.9119	0.0032	18	4	14	17	17	4	13	16
7847.5424	7847.5475	-0.0051	18	3	15	19	17	3	14	18
7847.5424	7847.5475	-0.0051	18	3	15	18	17	3	14	17
7847.5424	7847.5475	-0.0051	18	3	15	17	17	3	14	16
7862.8826	7862.8878	-0.0052	18	1	17	19	17	1	16	18
7893.6121	7893.6146	-0.0025	18	2	16	19	17	2	15	18
7893.6121	7893.6146	-0.0025	18	2	16	18	17	2	15	17
7893.6121	7893.6146	-0.0025	18	2	16	17	17	2	15	16

3.6 Benchmark – B3LYP calculations for water clusters

Table S86. Summary table of benchmarking studies. The A,B,C parameters are the rotational constants, and $\chi_{aa}, \chi_{bb} - \chi_{cc}$, the nuclear quadrupole coupling constants associated to the ^{14}N atom. The predicted dipole moment components, μ, for the a-, b-, and c-type transitions, and the predicted zero-point corrected relative energy, ΔE_{ZPC} is also shown. All theoretical parameters are expressed in the *principal axis system* (PAS).

Species	A MHz	B MHz	C MHz	μ_a D	μ_b D	μ_c D	χ_{aa} MHz	$\chi_{bb} - \chi_{cc}$ MHz	ΔE_{ZPC} kJ/mol
$\mathscr{O}_{1w}$									
M_1	1118	459	418	-1.8	1.7	1.4	-2.5	-1.3	0
M_2	1123	459	417	-1.9	-1.8	-0.9	-2.5	-1.3	+ 0.1
TS	1117	462	419	-1.6	1.3	0.2	-2.6	-1.2	+ 0.9
$\mathscr{C}_{1w}$	885	604	473	-3.1	1.2	0.1	-4.6	0.0	+ 5.7
$\mathscr{O}_{2w}$									
M_1	974	356	317	-2.4	1.3	-0.2	-2.9	-1.3	0
M_2	975	357	317	-2.4	-1.3	0.6	-2.9	-1.3	+ 0.0
TS	972	359	319	-1.8	-1.4	-0.8	-3.0	-1.1	+ 2.4
$\mathscr{O}_{3w}$									
M_4	847	273	242	-3.4	1.1	1.0	-3.6	-0.7	0
M_2	849	270	239	2.7	1.6	0.7	-3.6	-0.8	+ 0.3
M_1	841	271	240	2.7	-1.5	0.5	-3.6	-0.8	+ 0.4
M_6	842	278	248	-2.4	0.6	-0.7	-3.5	-0.5	+ 1.6
M_5	823	279	251	-2.4	-0.4	0.8	-3.6	-0.7	+ 1.7
M_3	825	278	248	-3.6	-2.2	-2.3	-3.7	-0.7	+ 3.0
$\mathscr{O}_{4w}$									
M_1	728	225	216	4.2	-1.1	-1.3	-3.6	-0.9	0
M_2	731	224	214	-4.1	-1.2	-1.7	-3.7	-0.9	+ 0.0
M_3	720	228	219	2.3	-2.0	0.0	-3.7	-0.9	+ 1.1
M_4	717	227	219	-6.3	-0.5	-0.2	-3.6	-1.0	+ 1.9

4 Large water clusters

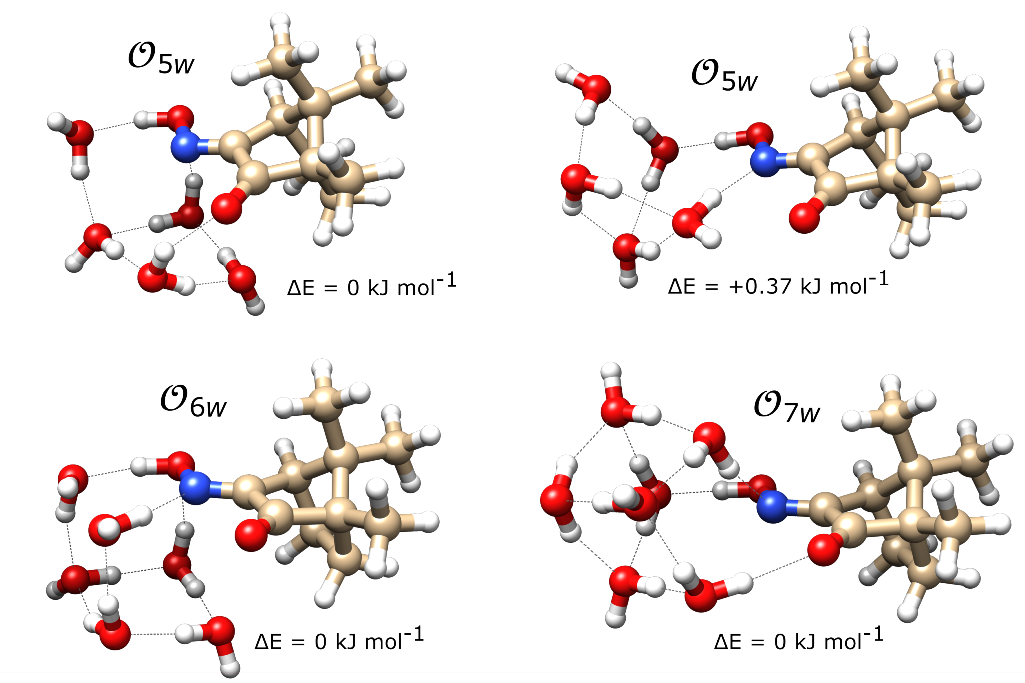

Figure S1. Predicted minima for the higher solvation orders around camphorquinone oxime: only the micro-hydrated structures of the open switch form with five, $\mathscr{O}_{5w}$, six, $\mathscr{O}_{6w}$, and seven water molecules, $\mathscr{O}_{7w}$, below 1 kJ mol^{-1} are shown.

Table S87. Predicted spectroscopic constants for the equilibrium structures of $\mathscr{O}_{5w}$, $\mathscr{O}_{6w}$ and $\mathscr{O}_{7w}$. The A,B,C parameters are the rotational constants, and $\chi_{aa}, \chi_{bb} - \chi_{cc}$, the nuclear quadrupole coupling constants associated to the ^{14}N atom. The predicted dipole moment components, μ, for the a-, b-, and c-type transitions, and the predicted zero-point corrected relative energy, ΔE_{ZPC} are also shown. All theoretical parameters are expressed in the *principal axis system*.

Constants	$\mathscr{O}_{5w}$ (M$_1$)	$\mathscr{O}_{5w}$ (M$_2$)	$\mathscr{O}_{6w}$ (M$_1$)	$\mathscr{O}_{7w}$ (M$_1$)
A /MHz	547	618	506	457
B /MHz	249	175	201	155
C /MHz	228	166	199	145
χ_{aa}	-2.8	-4.2	-1.9	-5.5
$\chi_{bb} - \chi_{cc}$ /MHz	3.8	-2.2	-3.5	-1.1
μ_a /D	-1.4	-3.1	2.5	3.0
μ_b /D	0.1	0.9	-1.4	0.9
μ_c /D	0.5	1.7	-0.2	1.2
ΔE_{ZPC} /kJ mol^{-1}	0	+0.4	0	0

Table S88. Equilibrium geometry of $\mathscr{O}_{5w}$ (M_1).

Atom	x /Å	y /Å	z /Å
C	-1.35146594181989	1.04219959055626	1.60982176028873
C	-1.89484669155067	-0.40401870946336	1.77894123674949
C	-2.24361623181933	-0.86626462095687	0.33083472450075
C	-0.87674105709159	-1.07803402159805	-0.30203268222465
C	-0.32727137872339	0.31199303971764	-0.46837431606897
C	-1.37687661493761	1.24094041121405	0.07504954334626
C	-3.16533758762894	-2.06199624454976	0.24248842470851
C	-2.66574427908318	0.47542400471148	-0.34761961927828
C	-2.82067945115411	0.36478154524251	-1.86754679878014
C	-3.94745222616371	1.08156410110224	0.22372679391063
O	-0.34610350727682	-2.13308261851048	-0.59207327270844
N	0.80926008650594	0.50855925244517	-1.02789929628079
O	1.13857666303338	1.85994420214686	-1.07928002093017
H	-1.98930175203570	1.77802705521113	2.10403872010199
H	-0.34458250301357	1.15174080433628	2.01558650580109
H	-1.15858529114670	-1.06613375048984	2.24032114991246
H	-2.79895875631618	-0.43287895654270	2.39289311294927
H	-1.29137713439650	2.27148077261273	-0.27074697657849
H	-3.35830009111761	-2.34046866959746	-0.79678141878534
H	-2.71085231993949	-2.92586753809313	0.73415271844613
H	-4.12062344118343	-1.85268783969537	0.73127365453844
H	-1.94569710754249	-0.07369334027591	-2.35585515873239
H	-3.68812558153459	-0.25130059828037	-2.12105236348887
H	-2.97887788686515	1.35677049669094	-2.30197446786015
H	-4.08804834038947	2.09212598016442	-0.17318719971096
H	-4.81432560890346	0.48604894867751	-0.07705578914592
H	-3.94987842776039	1.14688686126369	1.31199459511112
H	1.96795662395744	1.84289623960445	-1.62043474370799
O	3.49379245223095	1.54005694554272	-2.36576486719691
H	3.86389658287226	0.85870689029586	-1.76717056135363
H	3.43958361746080	1.11786682417677	-3.22993529881058
O	2.73415218642417	1.37257321149571	1.47617256974543
H	2.16298412839525	1.82438396189177	0.83718485358753
H	3.40441119038303	0.94178069265279	0.91601653498415
O	2.49956357325983	-2.23471809280848	0.14860306169687
H	2.17711089518442	-1.97954123183483	1.03387971242037
H	1.68882297062357	-2.25231079973219	-0.38554918300881
O	4.22469222463264	-0.26694983101406	-0.39012102183712
H	3.58749137377990	-1.02383604293321	-0.27782871168360
H	5.10241986950517	-0.64000715853540	-0.25401556728746
O	1.53877315110517	-0.91952708596757	2.46313294276562
H	1.76359247507611	-1.04426867885988	3.39135315372622
H	1.92827518506393	-0.05285731811403	2.21227784186857

Table S89. Equilibrium geometry of $\mathscr{O}_{5w}$ (M_2).

Atom	x /Å	y /Å	z /Å
C	-2.66284235456925	1.20881412910354	1.44679863429079
C	-3.17423722722022	-0.25528685537243	1.56682789911579
C	-2.81867738573796	-0.91226923013910	0.19789242574171
C	-1.30468901525174	-1.06237009125773	0.26538185978550
C	-0.79506193858879	0.34683142928852	0.17562871594402
C	-2.00693256405847	1.22506221076941	0.04455840790279
C	-3.56035685959672	-2.19356234815754	-0.11068246081888
C	-2.94058692644275	0.29635725363826	-0.78382399098238
C	-2.39112707713607	-0.00175107838212	-2.18220630439712
C	-4.36381263912637	0.83420762848610	-0.92974342532680
O	-0.66655051401191	-2.09102058754419	0.39100903099305
N	0.45850006386726	0.62057706588721	0.24146053561431
O	0.72813191742438	1.95759125604936	0.18162425106703
H	-3.48143084468819	1.92920103498152	1.50194261919326
H	-1.94277145002133	1.46983276654202	2.22527873943135
H	-2.71152437820264	-0.79783802278168	2.39637158760698
H	-4.25566106916187	-0.29932683113417	1.71910503935357
H	-1.80568420723075	2.21300940525265	-0.36982917687935
H	-3.25539173917382	-2.60725604926884	-1.07524760905236
H	-3.34725091928567	-2.94814199577289	0.65087759523338
H	-4.63992678161503	-2.02236241205492	-0.12981778150003
H	-3.02699060975560	-0.72631034873082	-2.69887266698136
H	-2.37438121609289	0.91341913356711	-2.78182801860889
H	-1.37338159392634	-0.40211227680228	-2.16619930543103
H	-4.34830280733637	1.77270251443956	-1.49299566273458
H	-4.98132420971474	0.12512881831019	-1.48858355783671
H	-4.85721961678944	1.02606244336372	0.02355406787675
H	1.72705268105838	1.99224120902790	0.22184717047751
O	4.35792474571272	-0.32252790024186	1.81380814434024
H	4.52526363629381	-0.38324429795275	2.75987958679213
H	3.58044404310768	-0.88715837312501	1.63334849003156
O	4.52047590500542	1.14655934727164	-2.12509821981458
H	5.32785840654181	1.44998711401244	-2.55131773695509
H	4.68773552504530	0.21784022948333	-1.86231821213006
O	2.28638601877068	-1.56634798230253	0.50422031726405
H	1.73162219447767	-0.77048793036109	0.34379375808237
H	1.61695040967957	-2.25788418499061	0.59838599840063
O	3.36352412311437	1.97508443787149	0.28157644042481
H	3.78571827808582	1.33472777914296	0.87766444219023
H	3.78059944368822	1.82535777274090	-0.59050214650015
O	4.80315612721662	-1.35972527792357	-0.94675471444961
H	3.89488204651096	-1.62102935311079	-0.71954185567034
H	5.17552830863431	-1.14035667912287	-0.08085182478447

Table S90. Equilibrium geometry of $\mathscr{O}_{6w}$ (M_1).

Atom	x /Å	y /Å	z /Å
C	1.49699228674293	1.92011540817335	-0.41142125335980
C	2.18849506137154	1.03402139546087	-1.48462208986786
C	2.62640892435798	-0.25079571464427	-0.71863129043088
C	1.31701776455167	-0.98355544754064	-0.46861777999151
C	0.60519479919548	-0.12051678729478	0.54187796786188
C	1.53006660958787	1.02116064188975	0.84906015194436
C	3.68581876503742	-1.08278196309474	-1.40591100528374
C	2.90662268331771	0.30292256240004	0.71696297537466
C	3.10503653709221	-0.79555885126369	1.76594917815178
C	4.09906745349670	1.25653983857099	0.78695953354949
O	0.92686219394733	-2.02847961573240	-0.94677592161931
N	-0.55704444860063	-0.45346779113226	0.96021211850780
O	-1.09166427201826	0.42692196923764	1.88690427376440
H	2.04619200356052	2.84668408570032	-0.23225225718801
H	0.47887118613773	2.18598392561885	-0.69508672592061
H	1.51365762874294	0.79065802571683	-2.30890637289938
H	3.07030506895082	1.51946803719277	-1.91108882878956
H	1.33431159498859	1.52601166533005	1.79581129595743
H	4.59799608895511	-0.50058993501599	-1.56123744559841
H	3.93401108773879	-1.97271932572058	-0.82189322346501
H	3.32570760776798	-1.42115453293409	-2.38091197624848
H	4.03795099203857	-1.33656502066421	1.58402313194764
H	3.16641636110830	-0.35303683575425	2.76499325125521
H	2.29246938095884	-1.52770802837797	1.77959300606315
H	4.07496931056664	2.03899866778250	0.02821085232492
H	4.12970791349121	1.74098551846775	1.76821565267541
H	5.03343562478896	0.70129422542807	0.66448768159794
H	-1.96987173213460	-0.00063476640104	2.08456268518007
O	-2.23105648912077	-2.62363266674310	0.08927435717874
H	-1.79860314050388	-3.45152871751920	-0.14933307403063
H	-1.49821169311679	-2.00948115578789	0.30527832564392
O	-3.26993965688743	-0.87900939174694	-1.94546257780452
H	-2.97343642129453	-1.60984955501908	-1.37459117598908
H	-3.94066396684488	-0.41662094873066	-1.41693821583375
O	-1.31472177839759	1.00471387754671	-2.28302360764758
H	-1.95256667529583	0.25402748781747	-2.22466227513492
H	-1.38501334163667	1.32144391305731	-3.19018524248990
O	-3.45013316208627	-0.84178951249770	2.07306943471265
H	-3.27965960893366	-1.68733954562312	1.62869825467554
H	-4.05135006543086	-0.36729437038710	1.46960244036048
O	-4.66058587685296	0.74739317524798	0.04508194256722
H	-5.48703752144049	1.24141339264534	0.06357707213327
H	-3.93554841146595	1.41900824690007	0.01881554658934
O	-2.43511941399140	2.29603778290861	-0.05666558958267
H	-1.98927898765688	1.97777758810652	-0.86803053504229
H	-1.88673215968356	1.96048699222589	0.66884309370064

Table S91. Equilibrium geometry of $\mathscr{O}_{7w}$ (M_1).

Atom	x /Å	y /Å	z /Å
C	3.23356327615525	-0.93224145269851	-1.57917844466772
C	3.69279655269895	-1.15334549818599	-0.10944202007510
C	3.09676011232242	0.05502997773292	0.67702363874138
C	1.61048705088608	-0.26532678397221	0.71134343579775
C	1.15347165060962	-0.05364750203926	-0.69827661072582
C	2.37245778132468	0.34986219709038	-1.47820424063260
C	3.71688836554853	0.30719985451950	2.03321151574227
C	3.12136722931504	1.18643976356242	-0.39927439125782
C	2.34745445731867	2.43931125157107	0.02367575622743
C	4.52839106549002	1.60738761674798	-0.82277256687524
O	0.95632670692034	-0.65109103023959	1.66405389981396
N	-0.07886257973228	-0.20868620875567	-1.02660475317516
O	-0.31245059614328	0.01253039214999	-2.35665545949511
H	4.08205102754797	-0.78052499618256	-2.24937976565073
H	2.65320335628291	-1.77093931468670	-1.96967352493225
H	3.34456455946603	-2.10415764994986	0.30420630929582
H	4.78170660236860	-1.14251202790927	-0.01565212705842
H	2.15836767139857	0.85093105891595	-2.42231300331427
H	3.24576956404234	1.15728123728595	2.53323642222512
H	3.58674997006324	-0.56590837131313	2.67776937189924
H	4.78761123953013	0.50689654551919	1.93938052471435
H	2.86608953299498	2.95342599025132	0.83801650863561
H	2.27553881245451	3.13647470526552	-0.81680831268756
H	1.32886170556727	2.22436917758859	0.35988965499465
H	4.46865086513236	2.28282603226803	-1.68209816229860
H	5.02198839382522	2.14993189152100	-0.01128542121885
H	5.16866168662348	0.76983998183634	-1.10142888101499
H	-1.29159141023621	-0.16496508980600	-2.43991538754468
O	-3.39336597594555	-2.35574081811543	-0.31498290157789
H	-3.50207252869341	-3.30373435005921	-0.44641187890843
H	-2.67263287583317	-2.26075805725110	0.35579773085641
O	-5.14775033061794	-0.44188205435398	0.94890666230389
H	-4.77327657354419	-1.20519368866446	0.47560937315465
H	-5.05805159163320	0.29349428601732	0.31879145412767
O	-4.12210299087933	1.57343065153527	-0.84095120870194
H	-3.30249754110240	1.65320404180149	-0.29436292144297
H	-4.37916542716003	2.46942824331286	-1.08191361059472
O	-1.91579301285192	1.40905078340732	0.71958686688034
H	-1.34746902496220	0.72825440857579	0.31994261082060
H	-2.21558873690104	1.03368624196545	1.56596916117389
O	-3.26948854490532	0.01262877209808	2.84409343012030
H	-4.05906296586077	-0.14963732348916	2.27131763804348
H	-3.60198946886472	0.25958832578611	3.71286134335534
O	-1.63981235392315	-2.04149773501448	1.72302200993839
H	-0.75367769884563	-1.65175519889202	1.64353667112477
H	-2.11314189808525	-1.45453116400757	2.33733608543506
O	-2.91089555887079	-0.49834049950987	-2.42682562630074
H	-3.43226089983164	0.24996648347121	-2.08875753891595
H	-3.07603799906380	-1.21913094310099	-1.79084611605481

5 Camphorquinone

Table S92. Experimental and theoretical spectroscopic constants for the camphorquinone oxime. The A,B,C parameters are the rotational constants. The predicted dipole moment components, μ, for the a-, b-, and c-type transitions, and the corresponding number of assigned lines is shown in parentheses. The standard error of the fit, σ is also shown. The theoretical parameters are expressed in the *principal axis system* (PAS).

Constants	Experimental	B3LYP-D3BJ
A /MHz	1241.4540(56)	1243
B /MHz	1014.85006(50)	1016
C /MHz	889.87546(41)	891
μ_a /D	yes(14)	4.9
μ_b /D	yes(2)	-1.3
μ_c /D	no	-0.9
σ /KHz	3.93	–

Table S93. Transition frequencies for Camphorquinone. J is the rotational angular momentum quantum number, K_a and K_c are the projections of J onto the principal axes at the prolate and oblate symmetric top limits, and F is the total angular momentum quantum number, which includes the nuclear spin, I(^{14}N) = 1.

Observed Frequency /MHz	Calculated Frequency /MHz	Difference / MHz	J	K_a	K_c	J'	K'_a	K'_c
3684.4701	3684.4764	-0.0064	2	1	2	1	1	1
3770.2625	3770.2593	0.0032	2	0	2	1	0	1
3934.4305	3934.4256	0.0048	2	1	1	1	1	0
5505.1041	5505.0963	0.0078	3	1	3	2	1	2
5577.5100	5577.5118	-0.0018	3	0	3	2	0	2
5714.1785	5714.1765	0.0020	3	2	2	2	2	1
5850.8451	5850.8413	0.0039	3	2	1	2	2	0
5871.8711	5871.8708	0.0003	3	1	2	2	1	1
7281.4400	7281.4383	0.0017	4	0	4	3	1	3
7309.0910	7309.0915	-0.0005	4	1	4	3	1	3
7349.8411	7349.8438	-0.0028	4	0	4	3	0	3
7377.4980	7377.4971	0.0009	4	1	4	3	0	3
7587.6643	7587.6677	-0.0034	4	2	3	3	2	2
7678.7624	7678.7634	-0.0010	4	3	2	3	3	1
7724.0832	7724.0875	-0.0044	4	3	1	3	3	0
7855.3318	7855.3324	-0.0006	4	2	2	3	2	1

Table S94. Equilibrium geometry of Camphorquinone.

Atom	*x* /Å	*y* /Å	*z* /Å
C	-2.69871963979076	-0.26126250220689	-0.30808118082560
C	-1.44476167062220	-0.50579749981242	0.58333736451610
C	-1.49854531342279	0.62182431097883	1.64894354742843
C	-2.78584588957563	1.40565556266770	1.27992534732495
C	-3.92092661572710	0.48423767775457	1.67305987461187
C	-3.85982701608543	-0.65388873577662	0.59670268680314
C	-2.68528984389148	-0.96228699015676	-1.64602654909252
C	-2.82832323241780	1.30362832641346	-0.27241785295651
C	-1.67929536947719	2.03540712537290	-0.96275043078844
C	-4.15068957679079	1.80825630692830	-0.85768129818349
H	-2.86280051573614	2.40146486928478	1.71107093591734
H	-1.82117491852883	-0.65961676691429	-2.23973526094781
H	-3.59002214160304	-0.74719807723396	-2.21619520291319
H	-2.64100685523576	-2.04336339769991	-1.50674587245097
H	-0.69585161963942	1.71231629480558	-0.62657127258861
H	-1.75764355275881	3.10888957172690	-0.77742028783942
H	-1.72763912919406	1.88424148009995	-2.04259715323728
H	-5.02634558859400	1.32603339736031	-0.41727673165423
H	-4.18790523844097	1.63247334250719	-1.93401493316166
H	-4.25296086863636	2.88247198805501	-0.69207612248913
O	-4.57572704813528	-1.61323370960015	0.53596219492267
O	-4.69488080866439	0.55748756011362	2.58519662995450
H	-0.54693866229301	-0.44239177965575	-0.03117365097168
H	-1.45984578614348	-1.50279291464505	1.02433225197716
H	-0.63169035622209	1.27823044919402	1.58873053889105
H	-1.53983274237318	0.23590411043868	2.66729242775334

References

1. Neese, F. Software update: The ORCA program system—Version 5.0. *Wiley Interdiscip. Rev. Comput. Mol. Sci.* **12**, e1606, DOI: 10.1002/wcms.1606 (2022).
2. Becke, A. D. Density-functional thermochemistry. iii. the role of exact exchange. *The J. chemical physics* **98**, 5648–5652, DOI: 10.1063/1.464913 (1993).
3. Lee, C., Yang, W. & Parr, R. G. Development of the colle-salvetti correlation-energy formula into a functional of the electron density. *Phys. review B* **37**, 785, DOI: 10.1103/PhysRevB.37.785 (1988).
4. Vosko, S. H., Wilk, L. & Nusair, M. Accurate spin-dependent electron liquid correlation energies for local spin density calculations: a critical analysis. *Can. J. physics* **58**, 1200–1211, DOI: 10.1139/p80-159 (1980).
5. Stephens, P. J., Devlin, F. J., Chabalowski, C. F. & Frisch, M. J. Ab initio calculation of vibrational absorption and circular dichroism spectra using density functional force fields. *The J. physical chemistry* **98**, 11623–11627, DOI: 10.1021/j100096a001 (1994).
6. Tao, J., Perdew, J. P., Staroverov, V. N. & Scuseria, G. E. Climbing the density functional ladder: Nonempirical meta–generalized gradient approximation designed for molecules and solids. *Phys. review letters* **91**, 146401, DOI: 10.1103/PhysRevLett.91.146401 (2003).
7. Staroverov, V. N., Scuseria, G. E., Tao, J. & Perdew, J. P. Comparative assessment of a new nonempirical density functional: Molecules and hydrogen-bonded complexes. *The J. chemical physics* **119**, 12129–12137, DOI: 10.1063/1.1626543 (2003).
8. Perdew, J. P., Burke, K. & Ernzerhof, M. Generalized gradient approximation made simple. *Phys. review letters* **77**, 3865, DOI: 10.1103/PhysRevLett.77.3865 (1996).
9. Grimme, S., Brandenburg, J. G., Bannwarth, C. & Hansen, A. Consistent structures and interactions by density functional theory with small atomic orbital basis sets. *The J. chemical physics* **143**, DOI: 10.1063/1.4927476 (2015).
10. Zhao, Y. & Truhlar, D. G. The m06 suite of density functionals for main group thermochemistry, thermochemical kinetics, noncovalent interactions, excited states, and transition elements: two new functionals and systematic testing of four m06-class functionals and 12 other functionals. *Theor. chemistry accounts* **120**, 215–241, DOI: 10.1007/s00214-007-0310-x (2008).
11. Møller, C. & Plesset, M. S. Note on an approximation treatment for many-electron systems. *Phys. review* **46**, 618, DOI: 10.1103/PhysRev.46.618 (1934).
12. Grimme, S., Goerigk, L. & Fink, R. F. Spin-component-scaled electron correlation methods. *Wiley Interdiscip. Rev. Comput. Mol. Sci.* **2**, 886–906, DOI: 10.1002/wcms.1110 (2012).
13. Scuseria, G. E., Scheiner, A. C., Lee, T. J., Rice, J. E. & Schaefer, H. F. The closed-shell coupled cluster single and double excitation (ccsd) model for the description of electron correlation. a comparison with configuration interaction (cisd) results. *The J. chemical physics* **86**, 2881–2890, DOI: 10.1063/1.452039 (1987).
14. Grimme, S. Exploration of chemical compound, conformer, and reaction space with meta-dynamics simulations based on tight-binding quantum chemical calculations. *J. chemical theory computation* **15**, 2847–2862, DOI: 10.1021/acs.jctc.9b00143 (2019).
15. Johnson, E. R. *et al.* Revealing noncovalent interactions. *J. Am. Chem. Soc.* **132**, 6498–6506, DOI: 10.1021/ja100936w (2010).
16. Lu, T. & Chen, Q. Visualization analysis of weak interactions in chemical systems. *Compr. Comput. Chem.* **2**, 240–264, DOI: 10.1016/B978-0-12-821978-2.00076-3 (2024).
17. Lu, T. & Chen, F. Multiwfn: A multifunctional wavefunction analyzer. *J. computational chemistry* **33**, 580–592, DOI: 10.1002/jcc.22885 (2012).
18. Lu, T. A comprehensive electron wavefunction analysis toolbox for chemists, Multiwfn. *The J. Chem. Phys.* **161**, DOI: 10.1063/5.0216272 (2024).
19. Pettersen, E. F. *et al.* UCSF Chimera—a visualization system for exploratory research and analysis. *J. computational chemistry* **25**, 1605–1612, DOI: 10.1002/jcc.20084 (2004).
20. Watson, J. K. G. Determination of Centrifugal Distortion Coefficients of Asymmetric-Top Molecules. *The J. Chem. Phys.* **46**, 1935–1949, DOI: 10.1063/1.1840957 (1967).
21. Western, C. PGOPHER, a Program for Simulating Rotational, Vibrational and Electronic Structure. *J. Quant. Spectrosc. & Radiat. Transf.* **186**, 221–242, DOI: 10.1016/j.jqsrt.2016.04.010 (2016).

22. Kisiel, Z. The PROSPE database of programs for rotational spectroscopy. (2004).
